# Fibrinogen-Like Protein 1 Is a Novel Biomarker for Predicting Disease Activity and Prognosis of Rheumatoid Arthritis

**DOI:** 10.3389/fimmu.2020.579228

**Published:** 2020-10-06

**Authors:** Shijia Liu, Yunke Guo, Lu Lu, Jiawei Lu, Mengying Ke, Tingting Xu, Yan Lu, Wenjun Chen, Jue Wang, Deshun Kong, Qiuxiang Shen, Youjuan Zhu, WenFeng Tan, Wei Ji, Wei Zhou

**Affiliations:** ^1^Department of Rheumatology and Immunology, Affiliated Hospital of Nanjing University of Chinese Medicine, Nanjing, China; ^2^State Key Laboratory of Natural Medicines, School of Traditional Chinese Pharmacy, China Pharmaceutical University, Nanjing, China; ^3^Jiangsu Collaborative Innovation Center of Chinese Medicinal Resources Industrialization, College of Pharmacy, Nanjing University of Chinese Medicine, Nanjing, China; ^4^Department of Rheumatology and Immunology, The First Affiliated Hospital With Nanjing Medical University, Nanjing, China

**Keywords:** rheumatoid arthritis, disease activity, TMT-labeled proteomics, biomarker, fibrinogen-like protein 1

## Abstract

Rheumatoid arthritis (RA), afflicting over 1% of the population, is an inflammatory joint disease leading to cartilage damage and ultimately impaired joint function. Disease-modifying anti-rheumatic drugs are considered as the first-line treatment to inhibit the progression of RA, and the treatment depends on the disease status assessment. The disease activity score 28 as clinical gold standard is extensively used for RA assessment, but it has the limitations of delayed assessment and the need for specialized expertise. It is necessary to discover biomarkers that can precisely monitor disease activity, and provide optimized treatment for RA patients. A total of 1,244 participants from two independent centers were divided into five cohorts. Cohorts 1–4 constituted sera samples of moderate to high active RA, low active RA, RA in remission and healthy subjects. Cohort 5 consisted of sera of RA, osteoarthritis (OA), ankylosing spondylitis (AS), systemic lupus erythematosus (SLE), primary Sjogren's syndrome (pSS) and healthy subjects. Biomarkers were found from cohorts 1–2 (screening sets), cohort 3 (discovery and external validation sets), cohort 4 (drug intervention set) and cohort 5 (biomarker-specific evaluation set). We found 68 upregulated and 74 downregulated proteins by TMT-labeled proteomics in cohort 1, and fibrinogen-like protein 1 (FGL1) had the highest area under the receiver operating characteristic curve (AUC) values in cohort 2. In cohort 3, in cross-comparison among moderate/high active RA, low active RA, RA in remission and healthy subjects, FGL1 had AUC values of approximately 0.9000 and predictive values of 90%. Additionally, FGL1 had a predictive value of 91.46% for moderate/high active RA vs. remission/low active RA and 80.77% for RA in remission vs. low active RA in cohort 4. Importantly, FGL1 levels had no significant difference in OA and AS compared with healthy persons. The concentrations in SLE and pSS were improved, but approximately 3-fold lower than that in active RA in cohort 5. In summary, FGL1 is a novel and specific biomarker that could be clinically useful for predicting progression of RA.

## Introduction

Rheumatoid arthritis (RA) is a chronic autoimmune disease that affects approximately 1% of the global population (1). It is characterized by inflammation of the synovial joints, which causes joint destruction, functional impairment, disability and premature mortality (2). Presently, RA is diagnosed by a total score between six and 10 based on the sum of the individual scores in the four domains in the 2010 Rheumatoid Arthritis Classification Criteria of the American College of Rheumatology/European League Against Rheumatism collaborative initiative (3). It has been reported that the cumulative disease activity score correlates well with RA progression and complications, and the existing target-to-treat strategy with the use of disease-modifying anti-rheumatic drugs (DMARDs) aggressively targets disease symptoms in hopes of achieving clinical remission (4). The treatment relies on the assessment of disease status (5, 6). For example, the American College of Rheumatology recommends different treatments for early (disease duration ≤ 6 months) and established (disease duration ≥ 6 months) RA with moderate/high disease activity, with low disease activity and in remission. Owing to the heterogeneous nature of the disease, patients show varied pharmacological responses to DMARDs. It would be ideal to monitor RA activity on a daily basis to optimize the treatment for RA (7). The erythrocyte sedimentation rate (ESR) and C-reactive protein (CRP) are the most common blood markers for the disease activity of RA, while they are neither highly sensitive nor specific to inherent changes in disease activity levels (8, 9). Rheumatoid factor (RF) and anticyclic citrullinated peptide antibody (ACPA) are representative serological markers for RA diagnosis and prognosis, including radiographic progression, but they do not usually correlate with disease progression (10). Urine soluble CD14 was reported to be a biomarker of high disease activity of RA, but the area under the receiver operating characteristic (ROC) curve was only 0.71 (11). The disease activity score 28 (DAS28), which is based on the number of tender joint, the number of swollen joint, and the ESR or CRP level, has been widely used for clinical assessment of RA. The level of disease activity can be interpreted as low (2.6<DAS ≤ 3.2), moderate (3.2<DAS≤5.1), or high (DAS >5.1). A DAS ≤2.6 represents remission according to the criteria of the European League Against Rheumatism (12, 13). However, the method has the limitations of delayed assessment and the need for specialized expertise. There are no precise universal or easy-to-use assessment methods that can facilitate the evaluation of disease activity and the prediction of disease severity. It is crucial to discover novel biomarkers to precisely monitor disease activity and severity of RA and provide personalized therapies by gaining insight into patients' pathogenic processes.

**Figure 1:**
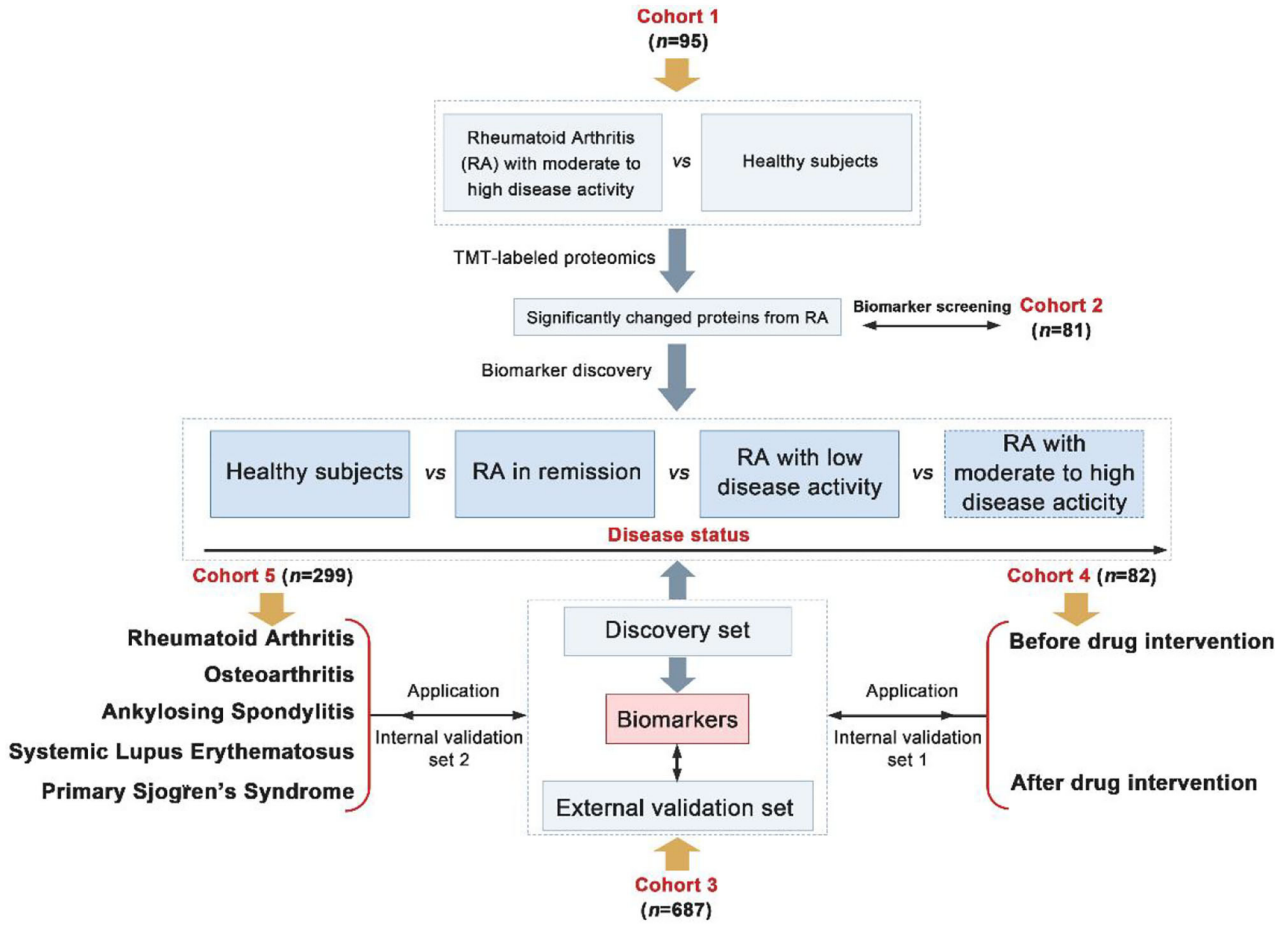
Overall strategy of biomarkers discovery for the assessment of disease activity and prognosis of Rheumatoid arthritis (RA) (Differentially expressed proteins were screened from fold change > 1.2 and *p* < 0.05).

Herein, a total of 1,244 participants recruited from two independent centers were divided into five cohorts. Cohorts 1–4 consisted of serum samples from persons with moderate/high active RA, low active RA, RA in remission and healthy subjects. Cohort 5 constituted samples from persons with RA, osteoarthritis (OA), ankylosing spondylitis (AS), systemic lupus erythematosus (SLE), primary Sjogren's syndrome (pSS) and healthy persons. Biomarkers were found in cohorts 1–2 using TMT-based quantitative proteomics as screening sets, cohort 3 as discovery and external validation sets, cohort 4 as drug intervention set and cohort 5 as biomarker-specific assessment set. The overall goal was to identify novel biomarkers that could evaluate the disease activity and prognosis of RA (Figure 1).

## Materials and Methods

### Subjects and Sample Collection

We recruited a total of 1,244 participants from two centers and divided them into five cohorts. Information on patients' clinical parameters was retrospectively collected from medical records. All procedures were approved by the medical ethics committee of the Affiliated Hospital of Nanjing University of Chinese Medicine and followed the tenets of the Declaration of Helsinki (2018NL-106-02). In cohort 1, there were 35 RA patients with moderate to high disease activity and 60 healthy individuals from the Affiliated Hospital of Nanjing University of Chinese Medicine (Table 1). Cohort 2 consisted of 38 RA patients with moderate to high disease activity, 15 RA patients in remission/with low disease activity and 28 healthy subjects from the Affiliated Hospital of Nanjing University of Chinese Medicine (Table 2). Cohort 3 consisted of 221 RA patients with moderate to high disease activity, 50 RA patients with low disease activity, 74 RA patients in remission and 182 healthy subjects from the Affiliated Hospital of Nanjing University of Chinese Medicine; and an additional 47 RA patients with moderate to high disease activity, 34 RA patients with low disease activity, 28 RA patients in remission and 51 healthy subjects from the First Affiliated Hospital with Nanjing Medical University (Tables 3, 4). Cohort 4 was composed of 82 RA patients with moderate to high disease activity before DMARD treatment and 23 RA patients with moderate to high disease activity, 26 RA patients with low disease activity and 33 RA patients in remission after DMARD intervention from the Affiliated Hospital of Nanjing University of Chinese Medicine (Table 5). In cohort 5, there were 35 healthy subjects, 47 RA patients (23 RA patients in remission and 24 RA patients with low to high disease activity), 50 OA patients (12 OA patients with Kellgren-Lawrence grade II and 38 OA patients with Kellgren-Lawrence grade III), 60 AS patients (27 patients with stable AS and 33 patients with active AS), 65 SLE patients (27 patients with inactive SLE and 38 patients with active SLE) and 42 pSS patients (32 patients with stable pSS to pSS with low disease activity and 10 patients with pSS with moderate to high disease activity) from the Affiliated Hospital of Nanjing University of Chinese Medicine (Table 6). The disease activity status of RA was defined according to the DAS28 score using a formula that included the ESR (12). The following subjects were excluded: current smokers and those with rheumatoid vasculitis, proven amyloidosis, infection, known thyroid disease, liver disease, pregnancy and malignancy. Radiographs of both feet and hands were analyzed by board-certified rheumatologists for all patients with RA. Each patient had suffered from RA for at least 6 months. All blood samples were centrifuged immediately at 2,000 × g for 10 min, and sera were transferred into clean Eppendorf tubes, and stored at −80°C before analysis.

**Table 1:** Baseline characteristics of rheumatoid arthritis (RA) patients with moderate to high disease activity and healthy subjects from cohort 1.

**Characteristics**	**Healthy subjects (*n* = 60)**	**RA with moderate to high disease activity (*n* = 35)**
Female, *n* (%)^‡^	48 (80)	28 (80)
Age (years) (mean ± SD) [min, max]	61 ± 17 [41, 79]	60 ± 10 [43, 78]
BMI (Kg/m^2^) (mean ± SD) [min, max]	21.84 ± 3.27 [16.97, 32.33]	21.41 ± 2.04 [16.61, 25.61]
Duration of disease (years) (mean ± SD) [min, max]	N/A	9.54 ± 8.51 [0.67, 30.00]
ESR (mm/h) (mean ± SD) [min, max]	6.70 ± 3.02 [2.00, 12.00]	63.86 ± 35.83*** [10.00, 85.00]
CRP (mg/L) (mean ± SD) [min, max]	3.08 ± 1.67 [1.10, 7.08]	34.79 ± 30.51*** [4.99, 77.00]
DAS28 (CRP)	N/A	4.46 ± 0.94 [3.00, 4.60]
DAS28 (ESR)	N/A	5.05 ± 0.98 [3.13, 7.67]
Rheumatoid factor, *n* (%) ||	N/A	33 (94.29)
ACPA, *n* (%) ||	N/A	35 (100)

BMI, Body mass index; ESR, Erythrocyte sedimentation rate; CRP, C reactive protein; DAS28, Disease Activity Score with 28-joint counts; ACPA, anti-cyclic citrullinated peptide. || antibody positivity. (^*^) significance by U-test.

‡ *significance by chi-square test. N/A, not applicable*.

**Table 2:** Baseline characteristics of remission to low active rheumatoid arthritis (RA), moderate to high active RA and healthy subjects from cohort 2.

**Characteristics**	**Healthy subjects (*n =* 28)**	**Remission to low active RA (*n =* 15)**	**RA with moderate to high active**
			**disease (*n =* 38)**
Female, *n* (%)^‡^	22 (78.57)	12 (80.00)	32 (84.21)
Age (years) (mean ± SD) [min, max]	51 ± 18 [29, 78]	51 ± 7 [41, 65]	58 ± 11 [27, 84]
BMI (Kg/m^2^) (mean ± SD) [min, max]	20.38 ± 2.57 [16.61, 25.95]	21.10 ± 3.29 [17.65, 29.76]	21.42 ± 3.23 [16.61, 27.68]
Duration of disease (year) (mean ± SD) [min, max]	N/A	13.00 ± 10.88 [2.00, 36.00]	12.62 ± 11.30 [0.60, 40.00]
ESR (mm/h) (mean ± SD) [min, max]	6.17 ± 3.01 [2.00, 15.00]	6.60 ± 3.70 [2.00, 15.00]	50.03 ± 30.97^&&&$$$^ [15.00, 120.00]
CRP (mg/L) (mean ± SD) [min, max]	4.31 ± 2.16 [0.20, 7.74]	2.40 ± 1.20 [1.11, 5.15]	27.36 ± 31.82^&&&$$$^ [1.00, 123.00]
DAS28 (CRP)	N/A	2.49 ± 0.67 [1.31, 3.26]	4.46 ± 0.94^$$$^ [3.00, 4.60]
DAS28 (ESR)	N/A	2.31 ± 0.62 [1.28, 3.20]	5.05 ± 0.98^$$$^ [3.13, 7.67]
Rheumatoid factor, *n* (%) ^||^^‡^	N/A	9 (60.00)	32 (84.21)
ACPA, *n* (%) ^||^^‡^	N/A	12 (80.00)	32 (84.21)

BMI, Body mass index; ESR, Erythrocyte sedimentation rate; CRP, C reactive protein; DAS28, Disease Activity Score with 28-joint counts; N/A, not applicable; ACPA, anti-cyclic citrullinated peptide. (^*^) significance with healthy subjects vs. RA in remission to low active disease, (^&^) significance with healthy subjects vs. RA with moderate to high active disease, (^$^) significance with remission to low active RA vs. RA with moderate to high active disease. (^*, &, $^) significance by one-way ANOVA with Kruskal-Wallis non-parametric test.

‡ *significance by chi-square test*.

|| *antibody positivity*.

**Table 3:** Baseline characteristics of rheumatoid arthritis (RA) in remission, RA with low disease activity, RA with moderate to high disease activity and healthy persons in discovery set from cohort 3.

**Characteristics**	**Healthy subjects**	**RA in remission**	**RA with low disease**	**RA with moderate to high disease**
	**(*n =* 182)**	**(*n* = 74)**	**activity (*n =* 50)**	**activity (*n =* 221)**
Female, *n* (%)	143 (78.57)	61 (83.56)	42 (84)	187 (84.62)
Age (years) (mean ± SD) [min, max]	57 ± 13 [30, 82]	57 ± 11 [37, 80]	59 ± 10 [29, 83]	60 ± 11 [19, 84]
BMI (Kg/m^2^) (mean ± SD) [min, max]	22.31 ± 5.17 [18.17, 31.99]	22.14 ± 4.19 [18.17, 31.03]	21.45 ± 6.19 [17.17, 33.09]	22.74 ± 4.88 [18.19, 31.99]
Duration of disease (year) (mean ± SD) [min, max]	N/A	12.77 ± 12.20 [0.50, 40.00]	10.77 ± 9.57 [0.50, 40.00]	10.26 ± 9.13 [0.50, 40.00]
ESR (mm/h) (mean ± SD) [min, max]	9.80 ± 6.15 [0.42, 19.91]	13.22 ± 12.44 [2.00, 72.00]	34.96 ± 26.23^$$$&&&^ [8.00, 85.00]	58.59 ± 31.48^***@@@∧∧∧^ [4.00, 140.00]
CRP (mg/L) (mean ± SD) [min, max]	3.93 ± 2.28 [0.02, 7.98]	3.66 ± 3.30 [1.00, 19.80]	6.81 ± 6.49 [1.04, 44.50]	31.59 ± 31.24^***@@@∧∧∧^ [1.24, 430.00]
DAS28 (CRP)	N/A	1.96 ± 0.52 [1.29, 3.25]	2.49 ± 0.44 [1.50, 3.26]	4.28 ± 1.04^***@@@^ [2.30, 7.10]
DAS28 (ESR)	N/A	1.92 ± 0.54 [0.50, 2.83]	2.91 ± 0.20^$^ [2.60, 3.30]	4.91 ± 1.09^***@@@^ [3.20, 7.80]
Rheumatoid factor, *n* (%) ^||^	N/A	50 (67.57)	36 (72.00)	181 (81.90)^!^
ACPA, *n* (%) ^||^	N/A	67 (90.54)	45 (90.00)	207 (93.67)

*BMI, Body mass index; ESR, Erythrocyte sedimentation rate; CRP, C reactive protein; DAS28, Disease Activity Score with 28-joint counts; N/A, not applicable; ACPA, anti-cyclic citrullinated peptide*.

|| antibody positivity. (^*, ‡^) significance with moderate to high active RA vs. RA with low disease activity; (^$, !^) significance with low active RA vs. RA in remission; (^#^) significance with RA in remission vs. healthy subjects; (^@, !^) significance with moderate to high active RA vs. RA in remission; (^∧^) significance with moderate to high active RA vs. healthy subjects; (^&^) significance with low active RA vs. healthy subjects. (^*, $, #, @, ∧, &^) significance by one-way ANOVA with Kruskal-Wallis non-parametric test. (^‡,′, !^) significance by chi-square test. ^||^ antibody positivity.

**Table 4:** Baseline characteristics of rheumatoid arthritis (RA) in remission, RA with low disease activity, RA with moderate to high disease activity and healthy persons in external validation set from cohort 3.

**Characteristics**	**Healthy subjects**	**RA in remission**	**RA with low disease**	**RA with moderate to high disease**
	**(*n =* 51)**	**(*n =* 28)**	**activity (*n =* 34)**	**activity (*n =* 47)**
Female, *n* (%)	38 (74.51)	20 (71.43)	27 (79.41)	39 (82.98)
Age (years) (mean ± SD) [min, max]	51.30 ± 14.83 [30, 81]	48.04 ± 9.82 [31, 72]	46.88 ± 9.74 [23, 63]	50.13 ± 12.69 [25, 93]
BMI (Kg/m^2^) (mean ± SD) [min, max]	21.91 ± 7.07 [17.27, 30.19]	22.11 ± 4.27 [19.07, 32.11]	21.01 ± 4.57 [18.61, 30.19]	22.55 ± 4.23 [19.07, 32.67]
Duration of disease (year) (mean ± SD) [min, max]	N/A	10.17 ± 9.58 [0.50, 30.00]	11.07 ± 10.50 [0.50, 31.00]	12.07 ± 10.55 [0.50, 31.00]
ESR (mm/h) (mean ± SD) [min, max]	10.72 ± 6.36 [1.07, 19.81]	7.79 ± 5.44 [2.00, 20.00]	19.33 ± 11.84^$^ [2.00, 20.00]	45.96 ± 29.81^∧∧∧@@@*^ [2.00, 20.00]
CRP (mg/L) (mean ± SD) [min, max]	4.53 ± 2.57 [0.34, 8.27]	3.77 ± 1.72 [3.14, 8.90]	3.69 ± 1.22 [3.14, 7.00]	14.10 ± 15.70^**@@∧^ [3.14, 85.10]
DAS28 (CRP)	N/A	1.85 ± 0.48 [1.47, 2.17]	2.21 ± 0.45 [1.47, 2.80]	3.98 ± 1.02^***@@@^ [2.40, 6.37]
DAS28 (ESR)	N/A	1.64 ± 0.64 [0.49, 2.60]	3.04 ± 0.34 [2.60, 3.98]	4.66 ± 0.95^***@@@^ [3.23, 6.82]
Rheumatoid factor, *n* (%) ||	N/A	19 (67.86)	23 (67.65)	39 (82.98)
ACPA, *n* (%) ||	N/A	25 (89.29)	31 (91.18)	44 (93.62)

BMI, Body mass index; ESR, Erythrocyte sedimentation rate; CRP, C reactive protein; DAS28, Disease Activity Score with 28-joint counts; N/A, not applicable; ACPA, anti-cyclic citrullinated peptide. ^||^ antibody positivity. (^*, ‡^) significance with moderate to high active RA vs. RA with low disease activity; (^$, ′^) significance with low active RA vs. RA in remission; (^#^) significance with RA in remission vs. healthy subjects; (^@, !^) significance with moderate to high active RA vs. RA in remission; (^∧^) significance with moderate to high active RA vs. healthy subjects; (^&^) significance with low active RA vs. healthy subjects. (^*, $, #, @, ∧, &^) significance by one-way ANOVA with Kruskal-Wallis non-parametric test. (^‡,′, !^) mean significance by chi-square test. ^||^ antibody positivity.

**Table 5:** Baseline characteristics of rheumatoid arthritis (RA) patients before and after treatment of conventional synthetic disease-modifying anti-rheumatic drugs (DMARDs) in cohort 4.

**Characteristics**	**Before treatment (*n =* 82)**	**After treatment (*n =* 82)**
Female, *n* (%)^‡^	71 (84.51)	
Age (years) (mean ± SD) [min, max]	58 ± 11 [29, 84]	
BMI (Kg/m^2^) (mean ± SD) [min, max]	22.18 ± 6.14 [17.09, 30.18]	
Duration of disease (year) (mean ± SD) [min, max]	9.47 ± 9.38 [0.50, 40.00]	
ESR (mm/h) (mean ± SD) [min, max]	78.00 ± 43.03^***^ [10.00, 135.00]	30.00 ± 21.91 [2.00, 80.00]
CRP (mg/L) (mean ± SD) [min, max]	45.67 ± 31.94^***^ [1.24, 156]	10.24 ± 15.98 [1.06, 91.60]
DAS28 (CRP)	4.11 ± 1.00^***^ [2.36, 6.80]	2.49 ± 0.88 [1.20, 5.00]
DAS28 (ESR)	4.77 ± 1.12^***^ [3.20, 7.60]	2.90 ± 1.04 [0.50, 5.10]
Moderate to high active RA, *n*	82	23
Low active RA, *n*	0	26
Remission RA, *n*	0	33
Rheumatoid factor, *n* (%) ^||^^‡^	67 (81.71)	68 (82.93)
ACPA, *n* (%) ^||^^‡^	79 (96.34)	79 (96.34)

BMI, Body mass index; ESR, Erythrocyte sedimentation rate; CRP, C reactive protein; DAS28, Disease Activity Score with 28-joint counts; ACPA, anti-cyclic citrullinated peptide; (^*^) significance by U-test.

‡ *significance by chi-square test*.

|| *antibody positivity*.

**Table 6:** Baseline characteristics of rheumatoid arthritis (RA), osteoarthritis (OA) in knee, ankylosing spondylitis (AS), systemic lupus erythematosus (SLE), and primary Sjogren's syndrome (pSS) from cohort 5.

**Characteristics**	**Healthy subjects**	**Low disease status**	**High disease status**
**Healthy subjects (*****n*** **= 35) vs. RA in remission (*****n*** **= 23)**			
**vs. RA with low to high disease activity (*****n*** **= 24)**			
Female, *n* (%)^‡^	28 (80.00)	20 (86.96)	20 (83.33)
Age (years) (mean ± SD) [min, max]	48.66 ± 14.18 [30, 83]	57.82 ± 11.97 [33, 77]	57.96 ± 12.88 [19, 80]
BMI (Kg/m^2^) (mean ± SD) [min, max]	21.23 ± 2.72 [17.99, 29.42]	21.29 ± 3.34 [17.58, 29.41]	21.55 ± 2.52 [17.30, 26.99]
Duration of disease (year) (mean ± SD) [min, max]	N/A	12.43 ± 11.70 [1.00, 40.00]	16.02 ± 12.38 [1.00, 40.00]
ESR (mm/h) (mean ± SD) [min, max]	9.25 ± 6.10 [0.42, 19.81]	18.00 ± 10.74 [2.00, 35.00]	50.84 ± 34.28^$$$&&&^ [12.00, 130.00]
CRP (mg/L) (mean ± SD) [min, max]	3.88 ± 1.94 [0.36, 7.37]	2.98 ± 1.82 [1.00, 8.16]	20.75 ± 23.53^$$&&&^ [1.10, 85.50]
DAS28 (CRP)	N/A	1.70 ± 0.44 [1.20, 2.40]	3.40 ± 0.73^$$$^ [2.20, 5.30]
DAS28 (ESR)	N/A	1.90 ± 0.51 [0.50, 2.50]	3.41 ± 0.68^$$$^ [2.60, 5.60]
Rheumatoid factor, *n* (%) ^||^^‡^	N/A	19 (82.61)	20 (83.33)
ACPA, *n* (%) ^||^^‡^	N/A	21 (91.30)	22 (91.67)
**Healthy subjects (*****n*** **= 35) vs. OA with K-L grade II (*****n*** **= 12) vs. OA with K-L grade III (*****n*** **= 38)**			
Female, *n* (%)^‡^	28 (80.00)	9 (75)	30 (78.95)
Age (years) (mean ± SD) [min, max]	48.66 ± 14.18 [30, 83]	48.39 ± 5.36 [36, 60]	46.35 ± 6.00 [31, 59]
BMI (Kg/m^2^) (mean ± SD) [min, max]	21.23 ± 2.72 [17.99, 29.42]	23.06 ± 3.01 [18.36, 27.68]	22.34 ± 3.23 [16.61, 29.41]
Duration of disease (year) (mean ± SD) [min, max]	N/A	2.5 ± 1.8 [1, 8]	2.8 ± 1.6 [1, 7]
**Healthy subjects (*****n*** **= 35) vs. stable AS (*****n*** **= 27) vs. OA active AS (*****n*** **= 33)**			
Female, *n* (%)^‡^	28 (80.00)	21 (77.78)	25 (75.76)
Age (years) (mean ± SD) [min, max]	48.66 ± 14.18 [30, 83]	47.48 ± 14.38 [26, 79]	46.30 ± 15.44 [18, 72]
BMI (Kg/m^2^) (mean ± SD) [min, max]	21.23 ± 2.72 [17.99, 29.42]	23.51 ± 3.95 [17.30, 31.14]	22.97 ± 3.79 [17.30, 30.45]
Duration of disease (year) (mean ± SD) [min, max]	N/A	8.60 ± 6.80 [1, 30]	10.21 ± 8.46 [0.8, 32]
ESR (mm/h) (mean ± SD) [min, max]	9.25 ± 6.10 [0.42, 19.81]	27.15 ± 23.57** [1, 80]	42.47 ± 29.29^$&&&^ [2, 100]
CRP (mg/L) (mean ± SD) [min, max]	3.88 ± 1.94 [0.36, 7.37]	9.64 ± 8.66* [1.38, 32.60]	17.03 ± 13.47^$&&&^ [2.37, 48.40]
BASDAI (mean ± SD) [min, max]	N/A	2.59 ± 0.60 [1.10, 3.40]	4.65 ± 0.91^$$$^ [3.50, 7.60]
HLA-B27, *n* (%)^||^^‡^	N/A	25 (92.59)	30 (90.91)
**Healthy subjects (*****n*** **= 35) vs. inactive SLE (*****n*** **= 27) vs. active SLE (*****n*** **= 38)**			
Female, *n* (%)^‡^	28 (80.00)	24 (88.89%)	34 (89.47)
Age (years) (mean ± SD) [min, max]	48.66 ± 14.18 [30, 83]	40.74 ± 11.67 [18, 64]	44.17 ± 14.13 [20, 75]
BMI (Kg/m^2^) (mean ± SD) [min, max]	21.23 ± 2.72 [17.99, 29.42]	22.25 ± 2.78 [17.30, 28.72]	20.75 ± 2.27 [17.99, 25.26]
Duration of disease (year) (mean ± SD) [min, max]	N/A	9.08 ± 4.68 [1.00, 20.00]	9.76 ± 8.44 [1.00, 32.00]
SLEDAI (mean ± SD) [min, max]	N/A	3.00 ± 1.10 [0, 4]	8.00 ± 3.21^$$$^ [5, 22]
24 h urine protein (mg/d)	N/A	79.22 ± 60.86 [22.00, 165.00]	144.23 ± 96.33^$^ [11.00, 348.00]
Complement C3 (g/L)	N/A	0.93 ± 0.69 [0.40, 2.48]	0.70 ± 0.13 [0.39, 0.90]
Complement C4 (g/L)	N/A	0.13 ± 0.07 [0.04, 0.28]	0.15 ± 0.04 [0.09, 0.25]
Immunoglobulin G (g/L)	N/A	16.11 ± 4.96 [7.72, 26.40]	15.22 ± 7.38 [5.49, 36.90]
Rheumatoid factor, *n* (%)^||^^‡^	N/A	8 (27.59)	15 (39.47)
Leukocyte count (mean ± SD) [min, max] (10^9^/L)	N/A	5.38 ± 1.63 [2.50, 9.98]	6.21 ± 3.95 [2.10, 25.00]
Hemoglobin (mean ± SD) [min, max] (g/L)	N/A	124.35 ± 22.69 [47.00, 156.00]	130.61 ± 28.94 [36.50, 173.00]
Platelet count (mean ± SD) [min, max] (10^9^/L)	N/A	200.73 ± 59.38 [91.00, 350.00]	194.21 ± 58.45 [85.00, 308.00]
Anti-ds DNA antibody <200, *n* (%)^‡^	N/A	22 (81.48)^‡^	21 (55.26)
Anti-ds DNA antibody>200, *n* (%)^‡^	N/A	5 (18.52)	17 (44.74)^‡^
Anti-nuclear antibody≥1:640, *n* (%)^‡^	N/A	5 (18.52)	26 (68.42)^‡^
Anti-Sm antibody, *n* (%)^||^^‡^	N/A	3 (11.11)	7 (18.42)
Anti-ribosome P protein antibody, *n* (%)^||^^‡^	N/A	3 (11.11)	5 (13.16)
Anti-histone antibody, *n* (%)^||^^‡^	N/A	3 (11.11)	10 (26.32)
Anti-nucleosome antibody, *n* (%)^||^^‡^	N/A	8 (29.63)	14 (36.84)
Anti-SSA, *n* (%)^||^^‡^	N/A	15 (55.56)	20 (52.63)
Anti-SSB, *n* (%)^||^^‡^	N/A	3 (11.11)	6 (15.79)
Direct Antiglobulin test, *n* (%)^||^^‡^	N/A	17 (62.96)	21 (55.26)
Indirect antiglobulin test, *n* (%)^||^^‡^	N/A	3 (11.11)	0 (0)
**Healthy subjects (*****n*** **= 35) vs. stable pSS to pSS with low disease activity (*****n*** **= 32) vs. pSS with moderate to high disease activity (*****n*** **= 10)**			
Female, *n* (%)^‡^	28 (80.00)	31 (96.88)	10 (100)
Age (years) (mean ± SD) [min, max]	48.66 ± 14.18 [30, 83]	56.06 ± 12.60 [27, 78]	55.40 ± 10.43 [43, 71]
BMI (Kg/m^2^) (mean ± SD) [min, max]	21.23 ± 2.72 [17.99, 29.42]	20.99 ± 3.10 [17.19, 29.41]	21.32 ± 2.25 [17.78, 25.95]
Duration of disease (year) (mean ± SD) [min, max]	N/A	7.25 ± 6.30 [0.60, 25.00]	6.20 ± 4.32 [2.00, 17.00]
Immunoglobulin G (g/L)	N/A	15.75 ± 4.65 [8.03, 28.40]	16.84 ± 4.67 [10.50, 26.90]
Rheumatoid factor, *n* (%)^||^^‡^	N/A	14 (43.75)	7 (70.00)
Leukocyte count (mean ± SD) [min, max] (10^9^/L)	N/A	4.59 ± 1.76 [2.40, 9.30]	5.68 ± 2.67 [3.20, 11.90]
Hemoglobin (mean ± SD) [min, max] (g/L)	N/A	121.06 ± 13.37 [94.00, 144.00]	124.70 ± 13.01 [98.00, 139.00]
Platelet count (mean ± SD) [min, max] (10^9^/L)	N/A	157.25 ± 60.78 [42.00, 294.00]	174.20 ± 43.16 [99.00, 244.00]
Anti-SSA, *n* (%)^||^^‡^	N/A	21 (65.63)	9 (90)
Anti-SSB, *n* (%)^||^^‡^	N/A	8 (25.00)	3 (30.00)
Anti-nuclear antibody≥1:640, *n* (%)^‡^	N/A	18 (56.25)	4 (40.00)

BMI, Body mass index; ESR, Erythrocyte sedimentation rate; CRP, C reactive protein. For Rheumatoid Arthritis (RA) disease, DAS28 means disease activity score with 28-joint counts. ACPA means anti-cyclic citrullinated peptide. (^*^) significance with healthy subjects vs. RA in remission; (^&^) significance with healthy subjects vs. RA with active disease activity; (^$^) significance with RA in remission vs. RA with active disease activity. For osteoarthritis (OA) disease, K-L grade means Kellgren-Lawrence grade. (*) significance with healthy subjects vs. OA with K-L grade II; (^&^) significance with healthy subjects vs. OA with K-L grade III; (^$^) significance with OA with K-L grade II vs. OA with K-L grade III. For ankylosing spondylitis (AS) disease, BASDAI means bath ankylosing spondylitis disease activity index. HLA-B27 means human leukocyte antigen B27. (^*^) significance with healthy subjects vs. stable AS; (^&^) significance with healthy subjects vs. active AS; (^$^) significance with stable AS vs. active AS. For systemic lupus erythematosus (SLE) disease, SLEDAI means systemic lupus erythematosus disease activity index renal domain. (^*^) significance with healthy subjects vs. inactive SLE; (^&^) significance with healthy subjects vs. active SLE; (^$^) significance with inactive SLE vs. active SLE. For primary Sjogren's syndrome (pSS) disease, ESSDAI means the European league against rheumatism sjögren's syndrome disease activity index. (^*^) significance with healthy subjects vs. pSS with no to low disease activity, (^&^) significance with healthy subjects vs. pSS with moderate to high disease activity, (^$^) significance with pSS with no to low disease activity vs. pSS with moderate to high disease activity. (^*, &, $^) significance by one-way ANOVA with Kruskal-Wallis non-parametric test.

‡ *means significance by chi-square test*.

|| *antibody positivity. N/A, not applicable*.

### TMT-Based Quantitative Serum Proteomics

The comparative proteomic method was described previously (14). The mass spectrometry proteomics data have been deposited to the ProteomeXchange Consortium (http://www.proteomexchange.org) via the PRIDE partner repository with the dataset identifier PXD021689.

### Enzyme-Linked Immunosorbent Assay (ELISA) Analysis of Biomarkers From Cohorts (2–5)

Human alpha-1-acid glycoprotein 2 (ORM2), phospholipase A2 (PLA2) and serum amyloid A2 (SAA2) ELISA kits were purchased from Elabscience Biotechnology Inc. (Houston, Texas, USA). The CRP ELISA kit was purchased from Beckman Coulter, Inc. (Fullerton, CA, USA). The protein-arginine deiminase type-4 (PADI4) ELISA kit was purchased from CUSABIO TECHNOLOGY LLC. (Houston, Texas, USA). Fibrinogen-like protein 1 (FGL1) was purchased from Biorbyt LLC (San Francisco, CA, USA). The serum protein levels in cohorts (2–5) were measured using 96 (Human) ELISA kits according to the manufacturer's protocol.

### Statistical Analysis

Results were expressed as the mean ± SD for continuous variables and as the number (percent) for categorical variables. The statistical significance of difference in proteomics between active RA and healthy subjects was calculated using a non-parametric test (Mann–Whitney U-test). For cross-comparison among groups, the Kruskal-Wallis test was used. For categorical data, the difference in prevalence was evaluated by a chi-square or Fisher's exact test. Receiver operating characteristic (ROC) analysis was utilized for diagnosis of different disease activity status. Statistical analyses were performed using SPSS software version 19.0 (IBM Corp., Armonk, New York). For each statistical analysis, a *p* < 0.05 was considered significance.

## Results

### Differentially Expressed Proteins in Cohort 1 Are Identified by TMT-Based Proteomics

**Figure 2:**
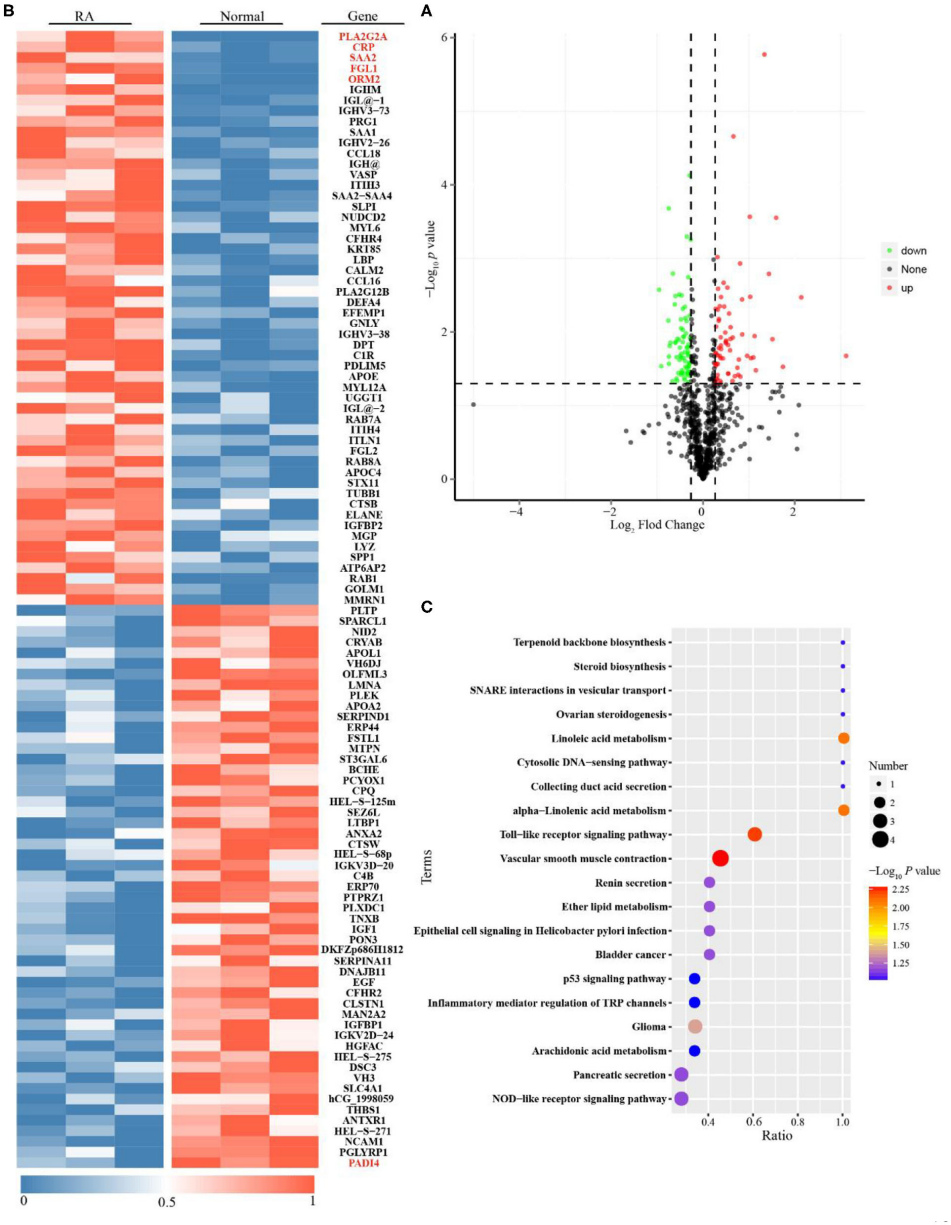
Proteomics analysis for Rheumatoid arthritis (RA) patients with moderate to high disease activity vs. healthy subjects. **(A)** Single volcano plots of the comparison of moderate to high active RA vs. healthy subjects (68 increased proteins are shown in volcano plots described in red color; 74 decreased proteins are displayed in volcano plots described in green color). **(B)** Heatmap of 142 differentially expressed proteins for the comparison of moderate to high active RA vs. healthy subjects. **(C)** KEGG enrichment analysis for differentially expressed proteins (The ratio means the protein numbers of significant differentially expressed proteins which located pathway entry divided into the total protein numbers of all annotated proteins which located pathway entry. When the ratio is greater, it means the degree of enrichment is higher. *P*-value was determined by hypergeometric test. When the *p*-value is closer to zero, the enrichment is more remarkable).

A comparative proteomic method was used to characterize the protein profiles. Supplementary Figure 1 shows good quality control of the proteomic data with regard to protein coverage, precursor ion tolerance, peptide length and protein mass. From these data, 762 proteins were identified as being differentially expressed in the sera of RA patients vs. healthy persons (Supplementary Table 1). We found 68 upregulated and 74 downregulated proteins in active RA patients compared to healthy subjects (Figure 2A and Supplementary Table 2). Among them, phospholipase A2 (PLA2), CRP, serum amyloid A2 (SAA2), Fibrinogen-like protein 1 (FGL1) and alpha-1-acid glycoprotein 2 (ORM2) were considered the most significantly upregulated immune/inflammation-related proteins, while protein-arginine deiminase type-4 (PADI4) was the most significantly downregulated immune/inflammation-related protein (Figure 2B). The GO database showed that the differentially expressed proteins, mostly existed in the extracellular region, had metal and calcium ion binding functions, and were involved in single-organism and organic substance metabolic processes (Supplementary Figure 2). The KEGG database showed that they were mainly involved in linoleic acid metabolism, alpha-linolenic acid metabolism and the Toll-like receptor signaling pathway (Figure 2C).

### Fibrinogen-Like Protein 1 Is a Novel Biomarker for Predicting Disease Activity and Prognosis of RA Based on Cohorts 2–5

**Figure 3:**
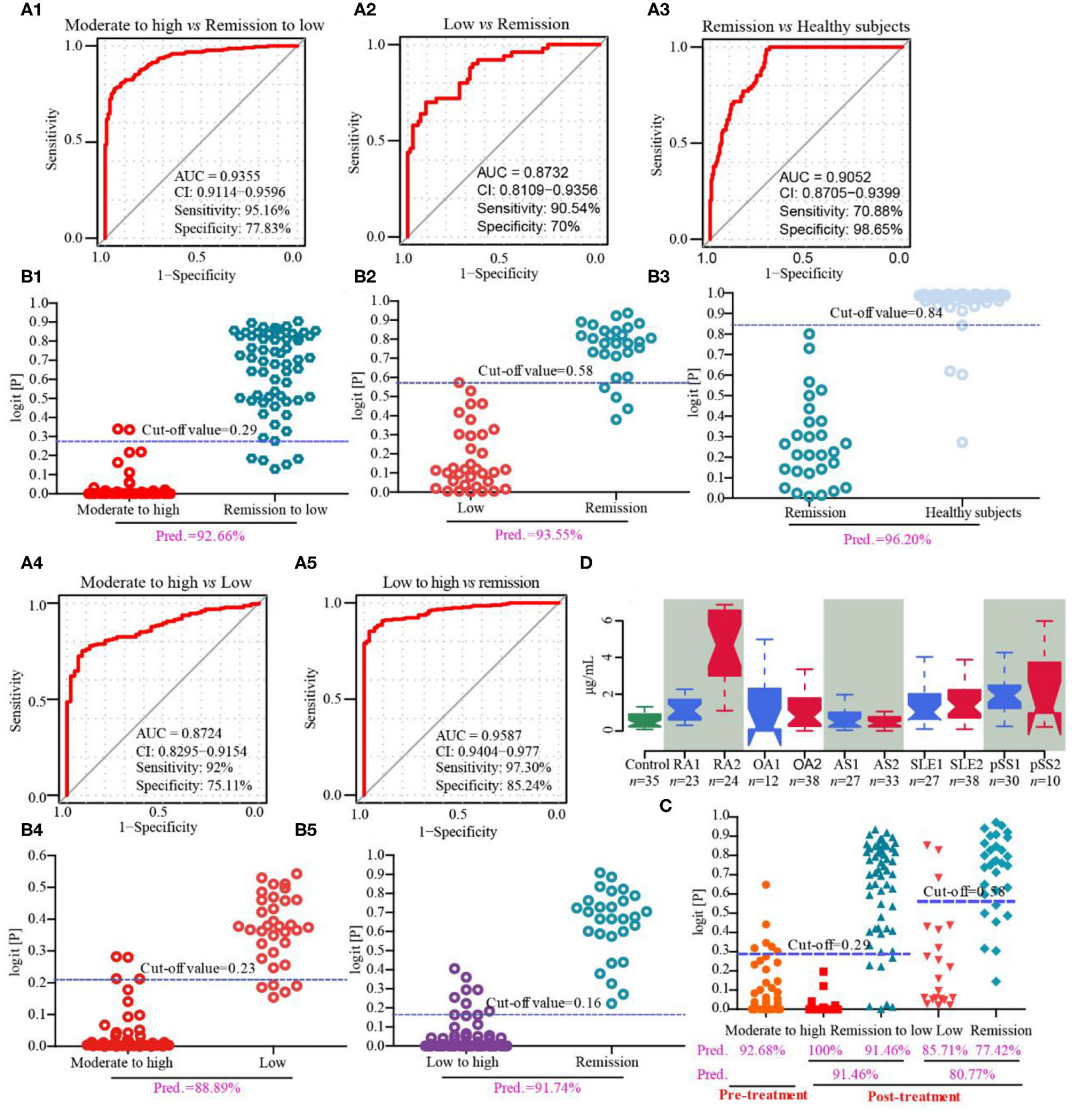
Discovery and validation of fibrinogen-like protein 1 (FGL1) as a novel biomarker from cohorts 3–5. **(A1,B1)**, respectively mean the receiver operating characteristic (ROC) curve in the discovery set and predictive value in the validation set of RA with moderate to high vs. remission to low disease activity from cohort 3; **(A2,B2)** mean the ROC curve in the discovery set and predictive value in the validation set, respectively, of RA with low disease activity vs. RA in remission from cohort 3; **(A3,B3)**, respectively mean the ROC curve in the discovery set and predictive value in the validation set of RA in remission vs. healthy subjects from cohort 3; **(A4,B4)** mean the ROC curve in the discovery set and predictive value in the validation phase, respectively, of moderate to high active RA vs. low active RA from cohort 3; **(A5,B5)**, respectively, mean the ROC curve in the discovery set and predictive value in the validation phase of low to high active RA vs. RA in remission from cohort 3. **(C)** represents the predictive values in drug intervention from cohort 4. **(D)** describes serum concentrations of FGL1 in healthy subjects (control), RA in remission (RA1), active RA (RA2), osteoarthritis with Kellgren-Lawrence grade II (OA1), osteoarthritis with Kellgren-Lawrence grade III (OA2), stable ankylosing spondylitis (AS1), active ankylosing spondylitis (AS2), inactive systemic lupus erythematosus (SLE1), active systemic lupus erythematosus (SLE2), stable primary Sjogren's syndrome to primary Sjogren's syndrome with low disease activity (pSS1) and primary Sjogren's syndrome with moderate to high disease activity (pSS2). There are no significant differences among control, OA1 and OA2 and among control, AS1 and AS2. It shows no significance in SLE1 vs. SLE2 and pSS vs. pSS2, but significance with control. It displays significance among control, RA1 and RA2. The statistical significance of difference is calculated using a Kruskal-Wallis test.

Cohort 2 (screening set), cohort 3 (discovery and validation sets), cohort 4 (drug intervention set) and cohort 5 (biomarker-specific evaluation set) were used to discover biomarkers for the assessment of disease activity and prognosis of RA. In cohort 2, we found that the serum concentrations, as determined by ELISA, of CRP and FGL1, but not those of PLA2, SAA2, ORM2, and PADI4 were consistent with the results of the proteomics analysis for the comparison of patients with RA with moderate to high disease activity vs. healthy subjects, while only FGL1 was significantly different in the comparison between RA patients in remission or with low disease activity and healthy subjects (Supplementary Figure 3). In cohort 3, we found that FGL1 can adequately differentiate among patients at different stages in the disease progression; its ability to differentiate among these patients was much better than those of CRP and ESR (Supplementary Figures 4, 5A). The area under the ROC curve (AUC) for the differentiation of RA patients with high/moderate disease activity from those in remission/with low disease activity was 0.935, the sensitivity was 95.16%, the specificity was 77.83% (95% confidence interval from 0.911 to 0.960), and the predictive value was 92.66% in the external validation set when using 0.29 as the cut-off value (Figures 3A1,B1 and Table 7). The AUC, sensitivity and specificity in RA patients with low disease activity vs. those in remission were 0.873, 90.54, and 70%, respectively, with a 95% confidence interval from 0.811 to 0.936, and the predictive value was 93.55% when 0.58 was used as the cut-off value (Figures 3A2,B2 and Table 7). For differentiation of RA patients in remission from healthy subjects, FGL1 had an AUC of 0.905, a sensitivity of 70.88%, a specificity of 98.65% with a 95% confidence interval from 0.871 to 0.940, and a predictive value of 96.20% when 0.84 was used as the cut-off value (Figures 3A3,B3 and Table 7). For the differentiation of RA patients with moderate to high disease activity from RA patients with low disease activity, FGL1 had an AUC of 0.872, a sensitivity of 92.00%, a specificity of 75.11% with a 95% confidence interval from 0.830 to 0.915, and predictive value of 88.89% when 0.23 was used as the cut-off value (Figures 3A4,B4 and Table 7). Meanwhile, the AUC, sensitivity and specificity for the differentiation of RA patients with low to high disease activity from those in remission were 0.959, 97.30, and 85.24% with a 95% confidence interval from 0.940 to 0.977, respectively. The predictive value when 0.16 was used as the cut-off value was 91.74% (Figures 3A5,B5 and Table 7). In cohort 4, we further validated that RA patients with moderate to high disease activity can be adequately distinguished from other RA patients, with predictive accuracies of 92.68% before intervention and 91.46% after drug treatment. Additionally, after treatment, RA patients with low disease activity and those in remission can be differentiated between with a predictive accuracy of 80.77% (Figure 3C and Supplementary Figure 5B). The analysis of cohort 4 showed that FGL1 is a potential biomarker for the prediction of prognosis. To evaluate the specificity of FGL1 for RA, the analysis was performed in cohort 5, and the results showed that the serum levels of FGL1 in OA and AS patients had no significant difference compared with that in healthy persons. The FGL1 level was 2.8-fold in active or inactive SLE and 3.8-fold in pSS with low or moderate to high disease activity, but ~10-fold in RA with low to high disease activity, higher than in healthy subjects (Figure 3D). The analysis in cohort 5 shows that FGL1 exhibits a high level of specificity in the pathogenesis of RA. Overall, the outcome of the study from cohorts 2–5 demonstrates that FGL1 is a good biomarker for the evaluation of disease activity and prognosis of RA.

**Table 7:** Parameters of unconditional logistic regression model for differentiating different disease status of rheumatoid arthritis (RA) in cohort 3.

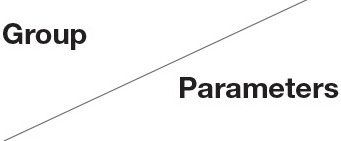	**Discovery set**					**External validation set**
	**AUC**	**Sensitivity (%)**	**Specificity (%)**	**95% confidence interval**	**Cut-off value**	**Predictive value (%)**
Moderate to high disease activity vs. Remission to low disease activity	0.935	95.16	77.83	0.911–0.960	0.29	92.66
Low disease activity vs. Remission	0.873	90.54	70	0.811–0.936	0.58	93.55
Remission vs. Healthy subjects	0.905	70.88	98.65	0.871–0.940	0.84	96.20
Moderate to high disease activity vs. Low disease activity	0.872	92	75.11	0.830–0.915	0.23	88.89
Low to high disease activity vs. Remission	0.959	97.30	85.24	0.940–0.977	0.16	91.74

## Discussion

This work describes a comprehensive comparative proteomics analysis of potential biomarkers for the assessment of disease activity and prognosis of RA; this was a large-scale (*n* = 1,244), two-center study. Proteomic phenotypes revealed 68 upregulated and 74 downregulated proteins among 762 proteins identified in the comparison of RA patients with moderate to high disease activity and healthy subjects, and the most differentially expressed immune-/inflammation-related proteins were PLA2, CRP, SAA2, FGL1, ORM2, and PADI4. The proteomics data strongly support an innate inflammatory pathogenesis in RA (15). Only FGL1 had an AUC of 0.9723 for moderate/high active vs. remission/low active RA, and an AUC of 0.8643 for remission/low active RA vs. healthy persons. We then found that FGL1 from cross-comparisons of moderate to high active RA, low active RA, remission and healthy subjects were ~0.9000 in AUC values and 90% in predictive values. FGL1 showed a good predictive accuracy for disease progression of pre- and post-treated RA patients. Importantly, we found the level of FGL1 in active RA was 10-fold higher than in OA, AS and healthy persons, and 3-fold higher than in SLE and pSS patients. These results suggest that FGL1 is a novel and specific biomarker for predicting progression of RA.

FGL1, called hepatocyte derived fibrinogen-related protein 1 or hepassocin, belongs to the fibrinogen family, with a high degree of amino acid homology with the carboxyl terminus of the fibrinogen β- and γ-subunits, but it does not have a platelet-binding site, cross-linking region, or thrombin-sensitive site, which are necessary for fibrin clot formation (16). FGL1 is primarily secreted by hepatocytes, and partial hepatectomy and IL-6 induce the promoter activity of FGL1, due to the STAT3 and hepatocyte nuclear factor-1 (HNF1) binding sites in the FGL1 promoter (17). FGL1 accelerates hepatocyte proliferation and protects against liver injury by activating the EGFR/ERK cascade through the Src-dependent pathway, exhibiting its potential to be used to treat fulminant hepatic failure in humans (18–20). However, it induces insulin resistance by the disruption of insulin signaling and induces non-alcoholic fatty liver disease (NAFLD) via the mechanism of hepatic lipid accumulation due to the activity of the ERK/JNK pathway. Increased FGL1 level is therefore a risk factor for both diabetes and NAFLD (21–23). FGL1 induces adipogenesis through an ERK1/2-C/EBPβ-dependent pathway. Blocking FGL1 as a therapeutic target may combat obesity (24). Importantly, FGL1 was reported to be a major inhibitory ligand for lymphocyte-activation gene 3 (LAG-3), an immune inhibitory receptor in antigen-specific T cells (25). The level of LAG-3^+^ regulatory T cells in peripheral blood was lower in RA patients with high disease activity than in healthy subjects, according to a small-scale study (26). It seems that the association of upregulated circulating FGL1 with disease activity is beneficial for the inhibition of autoimmunity but enhances the risks of obesity, diabetes and cardiovascular events (27–29). Finally, FGL1-LAG-3 pathway could become a novel intervention target for RA treatment.

Our study has a few limitations worth mentioning. Firstly, our recruited patients were suffered from RA for at least 6 months. Whether FGL1 plays a role in RA early diagnosis remains unclear. Secondly, we focused on the discovery of protein biomarkers, but ignored the metabolic biomarkers. Thirdly, only serum samples in RA patients were collected. In the future, early stage RA patients (<6 months) will be included to enhance the translational values of FGL1 in the clinical setting, and the combination of proteomics with metabolomics can be used to identify more potential specific biomarkers by analysis of serum, urine, feces, synovial fluid and T cells from blood based on a large-scale, multi-center study.

## Data Availability Statement

The datasets presented in this study can be found in online repositories. The names of the repository/repositories and accession number(s) can be found below: http://www.proteomexchange.org/, PXD021689.

## Ethics Statement

The studies involving human participants were reviewed and approved by Medical ethics committee of the Affiliated Hospital of Nanjing University of Chinese Medicine and the tenets of the Declaration of Helsinki (2018NL-106-02). The patients/participants provided their written informed consent to participate in this study.

## Author Contributions

WZ, SL, WJ, and YG: study design. LL, JL, MK, TX, YL, WC, JW, DK, QS, YZ, and WT: sample collection. WZ, SL, WJ, and YG: analyses of data. WZ, SL, and YG: interpretation of data. WZ and SL: writing and editing of the manuscript. All authors contributed to the article and approved the submitted version.

## Conflict of Interest

The authors declare that the research was conducted in the absence of any commercial or financial relationships that could be construed as a potential conflict of interest.
