# Supplementary material for: Fibrinogen-Like Protein 1 Is a Novel Biomarker for Predicting Disease Activity and Prognosis of Rheumatoid Arthritis
Source: Front Immunol. 2020 Oct 6;11:579228. doi: 10.3389/fimmu.2020.579228 (PMC7574527; doi:10.3389/fimmu.2020.579228)
Supplement: Supplementary file 1 [file Table_1.DOCX]

**Fibrinogen-like protein 1 is a novel biomarker for predicting disease activity and prognosis of rheumatoid arthritis**

Shijia Liu^a^, Yunke Guo^a^, Lu Lu^a^, Jiawei Lu^b^, Mengying Ke^c^, Tingting Xu^c^, Yan Lu^a^, Wenjun Chen^a^, Jue Wang^b^, Deshun Kong^c^, Qiuxiang Shen^c^, Youjuan Zhu^c^, WenFeng Tan^d^, Wei Ji^a*^, Wei Zhou^b*^

^a^ Department of Rheumatology and Immunology, Affiliated Hospital of Nanjing University of Chinese Medicine, Nanjing 210029, Jiangsu, China;

^b^ State Key Laboratory of Natural Medicines, School of Traditional Chinese Pharmacy, China Pharmaceutical University, Nanjing 210009, China;

^c^ College of Pharmacy, Jiangsu Collaborative Innovation Center of Chinese Medicinal Resources Industrialization, Nanjing University of Chinese Medicine, 210046, Nanjing, China;

^d^ Department of Rheumatology and Immunology, The First Affiliated Hospital with Nanjing Medical University, Nanjing 210029, China

Correspondence to:

^*b^Wei Zhou, School of Traditional Chinese Pharmacy, China Pharmaceutical University,

#639 Longmian Avenue, Jiangning District, Nanjing, 211198, P.R. China

wzhou@cpu.edu.cn

^*a^Wei Ji, Department of Rheumatology and Immunology, the Affiliated Hospital of Nanjing University of Chinese Medicine, #155 hanzhong Road, Qinhuai District, Nanjing, 210029, P.R. China

weiweiji1103@163.com

**Tables**

**Table S1** The identified proteins from the serum of patients with moderate to high active rheumatoid arthritis (RA) *vs* healthy subjects. The resulting spectra from each fraction were searched by Proteome Discoverer 2.2 (PD 2.2, Thermo). PD calculates protein abundance as the simple summation of its associated and used peptide group abundances.

**Table S2** The significantly changed proteins from serum of moderate to high active rheumatoid arthritis (RA) patients *vs* healthy subjects by fold change > 1.2 and *p* value < 0.05 screening.

**Figures**

**Figure S1** Evaluation of the proteomic data from the following parameters. A: protein coverage; B: precursor ion tolerance; C: peptide length; D: protein mass.

**Figure S2** GO functions for differential proteins (68 increased and 74 decreased proteins) from molecular function (A), biological process (B) and cellular component (C) angles. The differential proteins from serum of moderate to high active rheumatoid arthritis (RA) *vs* healthy subjects were analyzed based on fold change > 1.2 and *p* value < 0.05.

**Figure S3** The serum concentrations of C-reactive protein (CRP) (A), fibrinogen-like protein 1 (FGL1) (B), alpha-1-acid glycoprotein 2 (ORM2) (C), phospholipase A2 (PLA2) (D), serum amyloid A2 (SAA2) (E) and protein-arginine deiminase type-4 (PADI4) (F) in the cohort 2. AUC means area under receiver operating characteristic curve. (*) means significance with healthy subjects vs remission to low active rheumatoid arthritis (RA); (&) means significance with healthy subjects vs moderate to high active RA; ($) means significance with remission to low active RA vs moderate to high active RA; (ns) means non-significance. The significance of difference was analyzed by one-way ANOVA with Kruskal-Wallis nonparametric test.

**Figure S4** Receiver operating characteristic (ROC) curve of fibrinogen-like protein 1 (FGL1), C-reactive protein (CRP) and erythrocyte sedimentation rate (ESR) in cross-comparison among moderate to high active RA, low active RA, RA in remission and healthy subjects in the discovery set of the cohort 3. A: Moderate to high active RA *vs* remission to low active RA; B: Low active RA *vs* RA in remission; C: RA in remission *vs* healthy persons; D: Moderate to high active RA *vs* Low active RA; E: Low to high active RA *vs* RA in remission; F: The area under ROC curve (AUC) of FGL1, CRP and ESR in cross-comparison among moderate to high active RA, low active RA, RA in remission and healthy subjects.

**Figure S5** The serum concentrations of fibrinogen-like protein 1 (FGL1) in the cohort 3 (discovery set) (A) and cohort 4 (B). (^*^) means significance with moderate to high active RA *vs* RA with low disease activity; (^$^) means significance with low active RA *vs* RA in remission; (^#^) means significance with RA in remission *vs* healthy subjects; (^@^) means significance with moderate to high active RA *vs* RA in remission; (^^^) means significance with moderate to high active RA *vs* healthy subjects; (^&^) means significance with low active RA *vs* healthy subjects. (^!^) means significance with pre-treated RA *vs* post-treated RA. The significant difference was analyzed by one-way ANOVA with Kruskal-Wallis nonparametric test (A) and Mann−Whitney U test (B).

**Tables**

**Table S1** The identified proteins from the serum of patients with moderate to high active rheumatoid arthritis (RA) *vs* healthy subjects. The resulting spectra from each fraction were searched by Proteome Discoverer 2.2 (PD 2.2, Thermo). PD calculates protein abundance as the simple summation of its associated and used peptide group abundances.

| Protein | Description | Gene | PSMs | Unique_Peptides | RA1 | RA2 | RA3 | Healthy subjects1 | Healthy subjects2 | Healthy subjects3 | FC (RA *vs* Healthy subjects) | P value (RA vs Healthy subjects) |
| --- | --- | --- | --- | --- | --- | --- | --- | --- | --- | --- | --- | --- |
| A0A024QYT5 | Serpin peptidase inhibitor, clade E (Nexin, plasminogen activator inhibitor type 1), member 1, isoform CRA_b | SERPINE1 | 6 | 4 | 0.908 | 1.019 | 0.969 | 1.039 | 1.023 | 0.882 | 0.984 | 0.801 |
| A0A024QZI2 | FCGRT | hCG_1998059 | 2 | 1 | 0.608 | 0.898 | 0.668 | 1.025 | 1.042 | 1.378 | 0.631 | 0.047 |
| A0A024QZL1 | Proteoglycan 1, secretory granule, isoform CRA_a | PRG1 | 1 | 1 | 1.029 | 0.986 | 1.193 | 0.468 | 0.499 | 0.606 | 2.039 | 0.003 |
| A0A024R035 | Complement component 9, isoform CRA_a | C9 | 109 | 20 | 0.958 | 1.058 | 0.862 | 0.86 | 0.926 | 0.958 | 1.048 | 0.538 |
| A0A024R0V4 | Vasodilator-stimulated phosphoprotein isoform 1 | VASP | 2 | 2 | 1.118 | 0.983 | 1.338 | 0.752 | 0.535 | 0.715 | 1.718 | 0.024 |
| A0A024R1N1 | Myosin, heavy polypeptide 9, non-muscle, isoform CRA_a | MYH9 | 18 | 14 | 1.039 | 1.155 | 0.928 | 1.006 | 0.929 | 0.976 | 1.073 | 0.397 |
| A0A024R1P2 | Ras-related C3 botulinum toxin substrate 2 (Rho family, small GTP binding protein Rac2), isoform CRA_a | RAC2 | 3 | 3 | 1.204 | 1.144 | 1.035 | 1.075 | 0.941 | 1.034 | 1.109 | 0.158 |
| A0A024R1U8 | Insulin-like growth factor binding protein 4, isoform CRA_a | IGFBP4 | 6 | 3 | 1.184 | 1.229 | 0.918 | 1.003 | 0.925 | 1.248 | 1.049 | 0.723 |
| A0A024R1X8 | Junction plakoglobin, isoform CRA_a | JUP | 20 | 10 | 0.723 | 0.766 | 1.006 | 1.178 | 0.999 | 1.543 | 0.671 | 0.108 |
| A0A024R2B5 | Lipase, endothelial, isoform CRA_a | LIPG | 3 | 1 | 0.983 | 1.079 | 1.356 | 0.905 | 0.954 | 1.054 | 1.173 | 0.268 |
| A0A024R374 | Cathepsin B, isoform CRA_a | CTSB | 2 | 1 | 1.153 | 1.109 | 1.119 | 0.874 | 1.002 | 0.838 | 1.246 | 0.039 |
| A0A024R3H2 | Sortilin-related receptor, L(DLR class) A repeats-containing, isoform CRA_b | SORL1 | 3 | 3 | 0.973 | 0.966 | 1.056 | 0.806 | 0.84 | 0.872 | 1.189 | 0.015 |
| A0A024R3W7 | Eukaryotic translation elongation factor 1 beta 2, isoform CRA_a | EEF1B2 | 1 | 1 | 0.896 | 0.951 | 0.92 | 1.007 | 1.201 | 1.023 | 0.857 | 0.124 |
| A0A024R433 | Insulin-like growth factor binding protein 5, isoform CRA_a | IGFBP5 | 6 | 5 | 1.219 | 0.872 | 1.206 | 0.852 | 0.807 | 0.785 | 1.349 | 0.125 |
| A0A024R462 | Fibronectin 1, isoform CRA_n | FN1 | 1297 | 97 | 0.999 | 0.934 | 0.882 | 1.102 | 1.11 | 1.114 | 0.846 | 0.036 |
| A0A024R4H0 | Procollagen-lysine 1, 2-oxoglutarate 5-dioxygenase 1, isoform CRA_a | PLOD1 | 1 | 1 | 2.388 | 0.992 | 5.124 | 1.072 | 1.039 | 1.042 | 2.697 | 0.279 |
| A0A024R5J8 | Leucine rich repeat containing 54, isoform CRA_a | LRRC54 | 6 | 4 | 0.842 | 0.785 | 0.988 | 0.944 | 0.836 | 0.966 | 0.952 | 0.585 |
| A0A024R5L0 | Prolylcarboxypeptidase (Angiotensinase C), isoform CRA_a | PRCP | 4 | 4 | 0.952 | 1.046 | 0.925 | 1.072 | 1.007 | 1.11 | 0.917 | 0.138 |
| A0A024R5Z7 | Annexin | ANXA2 | 2 | 2 | 0.811 | 0.824 | 0.995 | 1.077 | 1.18 | 1.19 | 0.763 | 0.025 |
| A0A024R694 | Actinin, alpha 1, isoform CRA_a | ACTN1 | 9 | 4 | 1.113 | 1.202 | 1.039 | 1.052 | 0.941 | 1.058 | 1.099 | 0.174 |
| A0A024R6E8 | Galactosylceramidase, isoform CRA_c | GALC | 2 | 2 | 0.998 | 1.05 | 1.147 | 1.081 | 1.105 | 1.014 | 0.998 | 0.975 |
| A0A024R6K8 | Tryptophanyl-tRNA synthetase, isoform CRA_a | WARS | 6 | 3 | 0.913 | 1.02 | 0.942 | 0.84 | 0.892 | 0.744 | 1.161 | 0.074 |
| A0A024R6Y2 | Nuclear transport factor 2, isoform CRA_a | NUTF2 | 2 | 1 | 1.233 | 1.072 | 1.063 | 1.157 | 0.985 | 1.115 | 1.034 | 0.649 |
| A0A024R755 | Calumenin, isoform CRA_a | CALU | 16 | 1 | 0.886 | 0.895 | 1.176 | 1.218 | 1.072 | 0.971 | 0.907 | 0.448 |
| A0A024R868 | Angiopoietin-like 2, isoform CRA_a | ANGPTL2 | 1 | 1 | 1.047 | 1.115 | 0.974 | 0.988 | 0.972 | 1.052 | 1.041 | 0.442 |
| A0A024R8Q1 | Glucosidase, alpha acid (Pompe disease, glycogen storage disease type II), isoform CRA_a | GAA | 2 | 2 | 1.137 | 0.926 | 1.402 | 1.13 | 1.105 | 1.072 | 1.048 | 0.74 |
| A0A024R8S5 | Protein disulfide-isomerase | P4HB | 27 | 13 | 0.895 | 0.875 | 0.896 | 0.981 | 1.01 | 1.057 | 0.875 | 0.021 |
| A0A024R930 | Proteoglycan 4, isoform CRA_a | PRG4 | 48 | 17 | 0.974 | 0.891 | 0.848 | 0.853 | 0.959 | 0.943 | 0.985 | 0.791 |
| A0A024R971 | Fibromodulin, isoform CRA_a | FMOD | 2 | 1 | 0.982 | 1.086 | 0.981 | 1.122 | 1.213 | 1.226 | 0.857 | 0.023 |
| A0A024R9Q1 | Thrombospondin 1, isoform CRA_a | THBS1 | 257 | 2 | 0.671 | 0.64 | 0.909 | 1.129 | 1.152 | 1.351 | 0.611 | 0.014 |
| A0A024RA94 | Microfibrillar-associated protein 2, isoform CRA_d | MFAP2 | 1 | 1 | 0.668 | 0.772 | 0.993 | 1.139 | 1.006 | 1.125 | 0.744 | 0.083 |
| A0A024RAB9 | Complement component 1, q subcomponent, B chain, isoform CRA_a | C1QB | 58 | 6 | 0.98 | 0.919 | 1.323 | 0.874 | 0.764 | 0.855 | 1.292 | 0.186 |
| A0A024RAG6 | Complement component 1, q subcomponent, A chain, isoform CRA_a | C1QA | 41 | 3 | 1.053 | 1.053 | 0.972 | 0.83 | 0.831 | 0.931 | 1.187 | 0.021 |
| A0A024RB87 | RAP1B, member of RAS oncogene family, isoform CRA_a | RAP1B | 3 | 3 | 1.156 | 1.174 | 1.005 | 1.036 | 0.918 | 0.859 | 1.186 | 0.08 |
| A0A024RC29 | Desmocollin 3, isoform CRA_b | DSC3 | 1 | 1 | 0.703 | 0.82 | 0.973 | 1.156 | 1.26 | 1.377 | 0.658 | 0.014 |
| A0A024RD26 | G protein-coupled receptor 116, isoform CRA_a | GPR116 | 2 | 2 | 1.02 | 1.065 | 0.914 | 1.093 | 0.952 | 1.07 | 0.963 | 0.571 |
| A0A024RD39 | Platelet-activating factor acetylhydrolase | PLA2G7 | 3 | 2 | 0.754 | 0.752 | 1.008 | 1.244 | 1.094 | 0.983 | 0.757 | 0.078 |
| A0A024RD80 | Heat shock protein 90kDa alpha (Cytosolic), class B member 1, isoform CRA_a | HSP90AB1 | 13 | 3 | 1.046 | 0.997 | 1.03 | 1.456 | 1.277 | 0.924 | 0.84 | 0.339 |
| A0A024RDB8 | Heparanase, isoform CRA_a | HPSE | 10 | 6 | 0.845 | 0.876 | 0.921 | 0.957 | 1.021 | 0.97 | 0.896 | 0.027 |
| A0A024RDE6 | Secreted phosphoprotein 1 (Osteopontin, bone sialoprotein I, early T-lymphocyte activation 1), isoform CRA_c | SPP1 | 5 | 3 | 1.006 | 0.97 | 0.901 | 0.832 | 0.721 | 0.806 | 1.219 | 0.019 |
| A0A024RDE8 | PDZ and LIM domain 5, isoform CRA_c | PDLIM5 | 1 | 1 | 1.165 | 1.008 | 1.166 | 0.791 | 0.831 | 0.85 | 1.35 | 0.023 |
| A0A024RDL1 | Chaperonin containing TCP1, subunit 6A (Zeta 1), isoform CRA_a | CCT6A | 2 | 2 | 1.143 | 0.982 | 0.832 | 0.999 | 0.944 | 1.023 | 0.997 | 0.978 |
| A0A068LKQ2 | Ig heavy chain variable region (Fragment) |  | 9 | 3 | 1.187 | 1.018 | 1.117 | 0.997 | 0.919 | 0.952 | 1.158 | 0.073 |
| A0A068LKQ8 | Ig heavy chain variable region (Fragment) |  | 9 | 1 | 0.969 | 0.998 | 0.974 | 0.924 | 0.857 | 1.063 | 1.034 | 0.645 |
| A0A068LKR7 | Ig heavy chain variable region (Fragment) |  | 23 | 1 | 0.805 | 0.899 | 0.883 | 1.009 | 1.044 | 0.74 | 0.927 | 0.557 |
| A0A068LN03 | Ig heavy chain variable region (Fragment) |  | 8 | 1 | 0.965 | 1.09 | 1.67 | 1.006 | 1.127 | 1.161 | 1.131 | 0.579 |
| A0A068LN13 | Ig heavy chain variable region (Fragment) |  | 37 | 1 | 1.155 | 2.505 | 0.823 | 0.837 | 1.208 | 0.818 | 1.566 | 0.405 |
| A0A068LRW4 | Ig heavy chain variable region (Fragment) |  | 5 | 1 | 0.957 | 0.967 | 1.033 | 0.883 | 0.934 | 0.92 | 1.08 | 0.072 |
| A0A068LRW6 | Ig heavy chain variable region (Fragment) |  | 14 | 2 | 1.672 | 1.092 | 0.807 | 0.87 | 0.877 | 1.382 | 1.141 | 0.658 |
| A0A075B6I0 | Immunoglobulin lambda variable 8-61 | IGLV8-61 | 13 | 4 | 0.717 | 0.962 | 0.87 | 1.322 | 1.024 | 0.947 | 0.774 | 0.153 |
| A0A075B6I9 | Immunoglobulin lambda variable 7-46 | IGLV7-46 | 14 | 1 | 1.201 | 0.993 | 1.287 | 1.057 | 0.928 | 0.954 | 1.185 | 0.163 |
| A0A075B6J9 | Immunoglobulin lambda variable 2-18 | IGLV2-18 | 3 | 1 | 0.939 | 0.922 | 0.98 | 1.242 | 1.006 | 1.068 | 0.857 | 0.147 |
| A0A075B6K0 | Immunoglobulin lambda variable 3-16 | IGLV3-16 | 5 | 1 | 1.424 | 1.304 | 0.957 | 0.818 | 0.898 | 0.662 | 1.549 | 0.071 |
| A0A075B6R9 | Immunoglobulin kappa variable 2D-24 (non-functional) (Fragment) | IGKV2D-24 | 5 | 1 | 0.661 | 0.804 | 0.724 | 1.1 | 1.206 | 0.952 | 0.672 | 0.022 |
| A0A075B7B6 | Immunoglobulin heavy variable 4/OR15-8 (non-functional) (Fragment) | IGHV4OR15-8 | 3 | 2 | 0.736 | 0.76 | 0.871 | 0.754 | 1.287 | 1.079 | 0.759 | 0.243 |
| A0A087WSV8 | Nucleobindin 2, isoform CRA_b | NUCB2 | 1 | 1 | 0.946 | 1.164 | 1.06 | 1.037 | 1.018 | 1.051 | 1.021 | 0.769 |
| A0A087WTY6 | Neuroblastoma suppressor of tumorigenicity 1 | NBL1 | 1 | 1 | 0.854 | 0.887 | 0.783 | 1.199 | 1.054 | 0.964 | 0.785 | 0.06 |
| A0A087WV75 | Neural cell adhesion molecule 1 | NCAM1 | 20 | 1 | 0.821 | 0.756 | 0.74 | 1.251 | 1.283 | 1.352 | 0.596 | 0 |
| A0A087WVQ6 | Clathrin heavy chain | CLTC | 2 | 2 | 1.087 | 1.048 | 1.069 | 1.136 | 1.031 | 1.094 | 0.983 | 0.609 |
| A0A087WXB8 | ST3 beta-galactoside alpha-2,3-sialyltransferase 6, isoform CRA_b | ST3GAL6 | 2 | 2 | 0.799 | 0.907 | 0.95 | 1.037 | 1.171 | 1.127 | 0.796 | 0.02 |
| A0A087WXI5 | Cadherin-1 | CDH1 | 8 | 3 | 0.969 | 1.04 | 1.052 | 0.928 | 0.946 | 0.896 | 1.105 | 0.044 |
| A0A087WY68 | Proprotein convertase subtilisin/kexin type 6 | PCSK6 | 5 | 4 | 0.953 | 1.012 | 1.161 | 0.941 | 0.792 | 0.915 | 1.181 | 0.113 |
| A0A087X0Q4 | Immunoglobulin kappa variable 2-40 | IGKV2-40 | 38 | 1 | 0.859 | 0.696 | 1.228 | 0.825 | 1.01 | 0.928 | 1.007 | 0.972 |
| A0A090N7U9 | Retinoic acid receptor responder (Tazarotene induced) 2 | RARRES2 | 3 | 1 | 0.986 | 1.207 | 0.887 | 0.898 | 0.896 | 1.163 | 1.041 | 0.768 |
| A0A090N8Y2 | Protein disulfide-isomerase A4 | ERP70 | 14 | 10 | 0.874 | 0.862 | 0.736 | 1.146 | 1.112 | 1.091 | 0.738 | 0.014 |
| A0A096LPE2 | SAA2-SAA4 readthrough | SAA2-SAA4 | 36 | 5 | 0.928 | 1.115 | 1.216 | 0.67 | 0.614 | 0.657 | 1.68 | 0.031 |
| A0A0A0MRJ7 | Coagulation factor V | F5 | 219 | 42 | 0.922 | 0.894 | 0.841 | 0.878 | 0.963 | 0.92 | 0.962 | 0.367 |
| A0A0A0MRZ8 | Immunoglobulin kappa variable 3D-11 | IGKV3D-11 | 14 | 1 | 0.909 | 0.991 | 1.355 | 0.834 | 0.781 | 0.978 | 1.255 | 0.245 |
| A0A0A0MSD0 | Sushi, von Willebrand factor type A, EGF and pentraxin domain-containing protein 1 | SVEP1 | 7 | 7 | 0.865 | 2.333 | 0.837 | 0.776 | 0.746 | 0.758 | 1.77 | 0.358 |
| A0A0A0MT89 | Immunoglobulin kappa joining 1 | IGKJ1 | 1 | 1 | 0.806 | 0.746 | 0.846 | 0.956 | 1.292 | 1.116 | 0.713 | 0.07 |
| A0A0B4J1V2 | Immunoglobulin heavy variable 2-26 | IGHV2-26 | 2 | 2 | 1.523 | 1.159 | 1.223 | 0.605 | 0.737 | 0.665 | 1.946 | 0.021 |
| A0A0B4J1V6 | Immunoglobulin heavy variable 3-73 | IGHV3-73 | 15 | 1 | 0.889 | 1.195 | 1.026 | 0.503 | 0.533 | 0.471 | 2.063 | 0.023 |
| A0A0B4J1X5 | Immunoglobulin heavy variable 3-74 | IGHV3-74 | 31 | 2 | 0.993 | 0.952 | 1.032 | 0.948 | 0.906 | 0.954 | 1.06 | 0.124 |
| A0A0B4J1Y9 | Immunoglobulin heavy variable 3-72 | IGHV3-72 | 22 | 3 | 1.043 | 1.03 | 1.917 | 1.043 | 1.092 | 0.914 | 1.309 | 0.397 |
| A0A0B4J2D9 | Immunoglobulin kappa variable 1D-13 | IGKV1D-13 | 3 | 2 | 0.659 | 0.789 | 0.427 | 0.845 | 1.24 | 0.941 | 0.62 | 0.075 |
| A0A0C4DGQ5 | Calpain small subunit 1 | CAPNS1 | 1 | 1 | 0.787 | 1.06 | 0.851 | 1.151 | 1.063 | 1.057 | 0.825 | 0.134 |
| A0A0C4DH25 | Immunoglobulin kappa variable 3D-20 | IGKV3D-20 | 10 | 1 | 0.813 | 0.848 | 0.837 | 1.18 | 1.143 | 0.981 | 0.756 | 0.045 |
| A0A0C4DH26 | Immunoglobulin kappa variable 6D-41 (non-functional) (Fragment) | IGKV6D-41 | 2 | 1 | 0.483 | 0.959 | 0.531 | 1.025 | 1.485 | 0.854 | 0.586 | 0.131 |
| A0A0C4DH35 | Immunoglobulin heavy variable 3-35 (non-functional) (Fragment) | IGHV3-35 | 3 | 2 | 1.022 | 0.896 | 1.07 | 1.031 | 0.981 | 0.982 | 0.998 | 0.972 |
| A0A0C4DH36 | Immunoglobulin heavy variable 3-38 (non-functional) (Fragment) | IGHV3-38 | 2 | 2 | 1.139 | 1.271 | 1.121 | 0.82 | 0.823 | 0.861 | 1.41 | 0.014 |
| A0A0E3SU01 | Brain-derived neurotrophic factor | BDNF | 4 | 4 | 1.179 | 1.251 | 1.228 | 1.187 | 1.131 | 0.997 | 1.104 | 0.168 |
| A0A0F7T8I6 | IGHV4-31 protein (Fragment) | IGHV4-31 | 1 | 1 | 0.587 | 1.228 | 0.441 | 0.739 | 0.79 | 2.173 | 0.609 | 0.429 |
| A0A0F7TC28 | IGHV4-4 protein (Fragment) | IGHV4-4 | 3 | 1 | 0.664 | 0.787 | 1.074 | 1.898 | 0.764 | 0.827 | 0.724 | 0.481 |
| A0A0F7TD49 | IGHV3-7 protein (Fragment) | IGHV3-7 | 17 | 1 | 0.681 | 1.064 | 0.683 | 1.239 | 1.149 | 1.896 | 0.567 | 0.101 |
| A0A0G2JK05 | Uncharacterized protein |  | 3 | 2 | 0.935 | 0.91 | 1.182 | 1.005 | 0.947 | 0.957 | 1.04 | 0.698 |
| A0A0G2JPH5 | Leukocyte immunoglobulin-like receptor subfamily B member 2 | LILRB2 | 2 | 2 | 1.671 | 1.027 | 0.877 | 1.021 | 1.007 | 1.195 | 1.109 | 0.682 |
| A0A0G2JS06 | Immunoglobulin lambda variable 5-39 | IGLV5-39 | 2 | 1 | 0.626 | 0.679 | 2.055 | 0.834 | 0.768 | 1.001 | 1.291 | 0.645 |
| A0A0J9YX35 | Immunoglobulin heavy variable 3-64D | IGHV3-64D | 3 | 2 | 1.181 | 1.134 | 1.163 | 0.874 | 0.968 | 0.694 | 1.372 | 0.056 |
| A0A0R7FJH5 | Coagulation factor XII | F12 | 23 | 8 | 0.764 | 0.714 | 0.795 | 0.905 | 1.187 | 1.137 | 0.704 | 0.058 |
| A0A0S2Z3F6 | Cholesteryl ester transfer protein plasma isoform 1 (Fragment) | CETP | 30 | 7 | 0.89 | 0.907 | 1.016 | 0.955 | 1.083 | 0.922 | 0.951 | 0.486 |
| A0A0S2Z3G9 | Actinin alpha 4 isoform 1 (Fragment) | ACTN4 | 7 | 2 | 0.902 | 0.965 | 2.136 | 1.182 | 1.021 | 1.247 | 1.161 | 0.692 |
| A0A0S2Z3Y1 | Lectin galactoside-binding soluble 3 binding protein isoform 1 (Fragment) | LGALS3BP | 120 | 16 | 0.93 | 1.011 | 0.931 | 0.943 | 0.875 | 0.93 | 1.045 | 0.29 |
| A0A0S2Z428 | HCG2039812, isoform CRA_b (Fragment) | KRT6A | 153 | 2 | 0.5 | 0.539 | 0.752 | 0.966 | 0.966 | 2.505 | 0.404 | 0.226 |
| A0A0S2Z4F1 | EGF containing fibulin-like extracellular matrix protein 1 isoform 1 (Fragment) | EFEMP1 | 64 | 14 | 1.193 | 1.22 | 1.327 | 0.916 | 0.886 | 0.785 | 1.446 | 0.002 |
| A0A0S2Z4G4 | Tropomyosin 3 isoform 1 (Fragment) | TPM3 | 27 | 5 | 1.324 | 1.06 | 1.089 | 0.999 | 0.911 | 1.001 | 1.193 | 0.143 |
| A0A0S2Z4L3 | Protein S isoform 2 (Fragment) | PROS1 | 196 | 27 | 1.04 | 1.011 | 1.042 | 0.992 | 1.034 | 1.011 | 1.019 | 0.302 |
| A0A0X9T7T4 | MS-D3 heavy chain variable region (Fragment) |  | 53 | 1 | 1.061 | 1.063 | 1.974 | 2.35 | 1.233 | 1.134 | 0.869 | 0.699 |
| A0A0X9T7Y9 | IBM-A2 heavy chain variable region (Fragment) |  | 1 | 1 | 0.844 | 1.095 | 1.031 | 0.939 | 0.691 | 0.918 | 1.165 | 0.269 |
| A0A0X9TD23 | MS-A5 heavy chain variable region (Fragment) |  | 27 | 1 | 1.166 | 0.921 | 0.605 | 0.963 | 1.141 | 0.859 | 0.909 | 0.655 |
| A0A0X9TD47 | MS-D1 light chain variable region (Fragment) |  | 25 | 1 | 0.799 | 0.928 | 1.26 | 1.005 | 0.933 | 0.881 | 1.06 | 0.727 |
| A0A0X9TD88 | GCT-A3 heavy chain variable region (Fragment) |  | 3 | 1 | 1.036 | 0.926 | 0.868 | 1.08 | 1.028 | 0.997 | 0.911 | 0.195 |
| A0A0X9USK2 | MS-A6 heavy chain variable region (Fragment) |  | 1 | 1 | 1.049 | 0.878 | 0.914 | 1.121 | 1.322 | 1.179 | 0.784 | 0.031 |
| A0A0X9UWK7 | MS-D4 heavy chain variable region (Fragment) |  | 18 | 2 | 1.06 | 0.946 | 1.245 | 0.795 | 0.785 | 0.851 | 1.337 | 0.081 |
| A0A0X9UWL5 | GCT-A5 light chain variable region (Fragment) |  | 15 | 1 | 0.941 | 1.049 | 0.969 | 0.944 | 1.097 | 1.022 | 0.966 | 0.565 |
| A0A0X9UWM4 | IBM-A3 heavy chain variable region (Fragment) |  | 5 | 2 | 1.245 | 0.831 | 1.17 | 0.907 | 0.936 | 0.909 | 1.18 | 0.324 |
| A0A0X9V9B3 | MS-F1 light chain variable region (Fragment) |  | 10 | 2 | 0.767 | 0.912 | 1.172 | 1.056 | 1.048 | 1.012 | 0.915 | 0.534 |
| A0A0X9V9C4 | GCT-A8 heavy chain variable region (Fragment) |  | 16 | 1 | 1.528 | 0.973 | 1.919 | 0.421 | 0.435 | 0.543 | 3.159 | 0.064 |
| A0A0X9V9D6 | IBM-B2 light chain variable region (Fragment) |  | 6 | 2 | 0.885 | 0.852 | 0.957 | 1.174 | 1.176 | 0.842 | 0.844 | 0.271 |
| A0A109NGN6 | Proteasome subunit alpha type |  | 1 | 1 | 0.659 | 0.617 | 1.033 | 1.045 | 0.851 | 0.963 | 0.808 | 0.301 |
| A0A109PS54 | GCT-A8 light chain variable region (Fragment) |  | 39 | 2 | 1.483 | 1.102 | 1.012 | 1.289 | 1.018 | 0.939 | 1.108 | 0.553 |
| A0A109PSY4 | MS-A1 light chain variable region (Fragment) |  | 6 | 1 | 0.979 | 0.974 | 1.183 | 0.989 | 0.989 | 1.091 | 1.022 | 0.79 |
| A0A125QYY9 | IBM-B2 heavy chain variable region (Fragment) |  | 12 | 2 | 0.923 | 0.954 | 1.065 | 0.928 | 0.849 | 0.957 | 1.076 | 0.272 |
| A0A125U0U7 | MS-C1 heavy chain variable region (Fragment) |  | 8 | 1 | 0.754 | 0.747 | 0.829 | 0.955 | 1.088 | 1.09 | 0.743 | 0.012 |
| A0A125U0V1 | MS-F1 heavy chain variable region (Fragment) |  | 15 | 2 | 0.879 | 0.952 | 0.853 | 1.221 | 0.936 | 0.979 | 0.856 | 0.225 |
| A0A125U0V4 | GCT-A2 heavy chain variable region (Fragment) |  | 2 | 1 | 1.037 | 0.967 | 1.362 | 0.975 | 0.824 | 0.717 | 1.337 | 0.133 |
| A0A140TA29 | Complement C4-B | C4B | 1970 | 1 | 1.002 | 0.857 | 0.801 | 0.712 | 0.863 | 0.805 | 1.118 | 0.282 |
| A0A140TA33 | Tenascin-X | TNXB | 14 | 13 | 0.895 | 0.805 | 0.783 | 1.161 | 1.164 | 1.103 | 0.724 | 0.003 |
| A0A140VJI7 | Testicular tissue protein Li 61 |  | 75 | 19 | 1.051 | 1.079 | 0.91 | 0.943 | 0.959 | 0.923 | 1.076 | 0.303 |
| A0A140VJJ2 | S-formylglutathione hydrolase | ESD | 3 | 3 | 1.772 | 1.022 | 0.986 | 0.916 | 1.036 | 1.014 | 1.274 | 0.401 |
| A0A140VK00 | Testicular tissue protein Li 227 |  | 3 | 3 | 1.098 | 0.863 | 0.953 | 1.062 | 0.896 | 1.044 | 0.971 | 0.751 |
| A0A140VK24 | Testicular secretory protein Li 24 |  | 34 | 7 | 0.942 | 0.9 | 1.118 | 1.028 | 1.086 | 0.987 | 0.955 | 0.571 |
| A0A140VK27 | Leukotriene A(4) hydrolase |  | 1 | 1 | 0.782 | 0.86 | 0.629 | 0.817 | 0.887 | 0.818 | 0.901 | 0.345 |
| A0A193CHR8 | 10E8 light chain variable region (Fragment) |  | 1 | 1 | 0.925 | 0.709 | 0.612 | 1.223 | 1.364 | 1.128 | 0.605 | 0.016 |
| A0A193CHS1 | 10E8 light chain variable region (Fragment) |  | 5 | 2 | 1.426 | 1.011 | 0.972 | 0.517 | 0.592 | 0.611 | 1.981 | 0.056 |
| A0A1B0GTC6 | Uncharacterized protein C3orf85 | C3orf85 | 3 | 1 | 0.898 | 0.92 | 0.781 | 1.017 | 0.944 | 0.976 | 0.885 | 0.103 |
| A0A1B1CYC5 | Vitamin D binding protein (Fragment) | Gc | 6 | 2 | 1.485 | 0.997 | 1.026 | 1.126 | 0.71 | 0.788 | 1.337 | 0.224 |
| A0A1C9J6R2 | B cell receptor heavy chain variable region (Fragment) |  | 17 | 0 | 0.758 | 0.781 | 0.511 | 1.044 | 1.984 | 0.975 | 0.512 | 0.177 |
| A0A1C9J6Z4 | B cell receptor heavy chain variable region (Fragment) |  | 16 | 1 | 0.905 | 0.867 | 0.813 | 1.135 | 0.861 | 0.928 | 0.884 | 0.302 |
| A0A1K0GXZ1 | Globin C1 | GLNC1 | 27 | 4 | 1.482 | 1.992 | 0.684 | 1.419 | 0.717 | 1.035 | 1.311 | 0.5 |
| A0A1L2BU38 | Anti-staphylococcal enterotoxin E heavy chain variable region (Fragment) |  | 2 | 1 | 0.804 | 1.027 | 0.841 | 1.104 | 1.196 | 1.366 | 0.729 | 0.033 |
| A0A1L2BU40 | Anti-staphylococcal enterotoxin E variable region lambda chain (Fragment) |  | 18 | 1 | 0.599 | 0.778 | 0.588 | 0.908 | 1.07 | 1.003 | 0.659 | 0.014 |
| A0A1S5UZ07 | Talin-1 | TLN1 | 20 | 18 | 1.016 | 1.291 | 0.991 | 0.865 | 0.789 | 0.834 | 1.326 | 0.1 |
| A0A1U9X8X6 | CDSN |  | 5 | 3 | 0.783 | 0.862 | 1.195 | 0.996 | 1.005 | 1.138 | 0.905 | 0.522 |
| A0A1W6IYI5 | N90-VRC38.08 heavy chain variable region (Fragment) |  | 13 | 1 | 0.917 | 0.843 | 0.941 | 0.832 | 0.837 | 0.84 | 1.076 | 0.161 |
| A0A1W6IYI8 | N90-VRC38.10 heavy chain variable region (Fragment) |  | 3 | 1 | 0.749 | 1.69 | 0.797 | 1.059 | 1.064 | 1.304 | 0.944 | 0.856 |
| A0A1W6IYI9 | N90-VRC38.04 heavy chain variable region (Fragment) |  | 3 | 2 | 1.079 | 1.153 | 1.287 | 1.116 | 1.177 | 1.167 | 1.017 | 0.781 |
| A0A1W6IYJ0 | N90-VRC38.03 heavy chain variable region (Fragment) |  | 1 | 1 | 0.746 | 0.81 | 0.922 | 1.306 | 1.184 | 1.083 | 0.693 | 0.013 |
| A0A1W6IYJ9 | N90-VRC38.01 light chain variable region (Fragment) |  | 4 | 2 | 0.872 | 1.037 | 0.992 | 1.065 | 1.276 | 1.093 | 0.845 | 0.103 |
| A0A218KGR2 | Amyloid beta A4 protein isoform a | APP | 74 | 15 | 1.012 | 1.074 | 1.059 | 1.018 | 0.955 | 0.965 | 1.071 | 0.063 |
| A0A286YFJ8 | Immunoglobulin heavy constant gamma 4 (Fragment) | IGHG4 | 190 | 1 | 1.346 | 0.969 | 1.423 | 0.966 | 0.698 | 0.845 | 1.49 | 0.08 |
| A0A288Q9P9 | MHC class I antigen (Fragment) | HLA-B | 1 | 1 | 1.004 | 0.907 | 1.127 | 0.733 | 0.792 | 0.826 | 1.292 | 0.052 |
| A0A2P9ATZ6 | Uncharacterized protein | BQ8482_480129 | 2 | 1 | 1.187 | 0.997 | 1.083 | 0.948 | 0.921 | 1.153 | 1.081 | 0.426 |
| A0A2Q2TTZ9 | Immunoglobulin kappa variable 1-33 | IGKV1D-33 | 12 | 1 | 0.97 | 0.851 | 1.052 | 0.911 | 0.974 | 1.02 | 0.989 | 0.881 |
| A0A2R8Y3M9 | Uncharacterized protein |  | 10 | 6 | 1.04 | 1.055 | 1.035 | 0.935 | 0.836 | 0.795 | 1.22 | 0.044 |
| A0N5G1 | Rheumatoid factor C6 light chain (Fragment) | V-kappa-1 | 16 | 1 | 0.937 | 0.779 | 1.242 | 0.905 | 0.812 | 0.95 | 1.109 | 0.556 |
| A0N5G3 | Rheumatoid factor G9 light chain (Fragment) | V-lambda-3 | 36 | 3 | 0.771 | 1.522 | 1.302 | 1.008 | 1.282 | 1.012 | 1.089 | 0.715 |
| A0N5G7 | Rheumatoid factor D5 heavy chain (Fragment) | VH3 | 2 | 1 | 0.905 | 0.761 | 0.915 | 1.365 | 1.287 | 1.302 | 0.653 | 0.004 |
| A0N7J6 | REV25-2 (Fragment) |  | 4 | 2 | 1.15 | 0.952 | 0.991 | 0.816 | 1.173 | 0.954 | 1.051 | 0.704 |
| A1L4H1 | Soluble scavenger receptor cysteine-rich domain-containing protein SSC5D | SSC5D | 4 | 3 | 0.967 | 0.963 | 0.967 | 1.063 | 0.963 | 0.976 | 0.965 | 0.376 |
| A2IPI2 | HRV Fab N27-VL (Fragment) |  | 15 | 1 | 0.651 | 0.92 | 0.806 | 0.788 | 1.062 | 1.547 | 0.7 | 0.261 |
| A2IPI4 | HRV Fab 025-VL (Fragment) |  | 5 | 2 | 0.945 | 0.895 | 1.235 | 0.966 | 0.964 | 0.976 | 1.058 | 0.649 |
| A2IPI6 | HRV Fab 027-VL (Fragment) |  | 6 | 1 | 0.765 | 1.009 | 0.708 | 1.144 | 1.114 | 1.35 | 0.688 | 0.036 |
| A2J1M3 | Rheumatoid factor RF-ET5 (Fragment) |  | 8 | 1 | 1.201 | 0.917 | 1.536 | 0.912 | 0.881 | 0.879 | 1.368 | 0.208 |
| A2J1M8 | Rheumatoid factor RF-IP12 (Fragment) |  | 6 | 1 | 1.157 | 1.634 | 1.438 | 0.974 | 0.894 | 0.926 | 1.514 | 0.071 |
| A2J1N5 | Rheumatoid factor RF-ET6 (Fragment) |  | 20 | 1 | 1.096 | 1 | 1.398 | 0.647 | 0.598 | 0.741 | 1.76 | 0.04 |
| A2J1N6 | Rheumatoid factor RF-ET9 (Fragment) |  | 15 | 2 | 0.974 | 0.849 | 1.285 | 0.639 | 0.763 | 0.691 | 1.484 | 0.112 |
| A2J1N7 | Rheumatoid factor RF-ET10 (Fragment) |  | 3 | 1 | 1.034 | 0.985 | 0.759 | 1.248 | 1.304 | 1.018 | 0.778 | 0.097 |
| A2J1N9 | Rheumatoid factor RF-ET12 (Fragment) |  | 11 | 1 | 0.638 | 1.37 | 2.052 | 0.653 | 0.615 | 0.469 | 2.337 | 0.196 |
| A2MYE1 | A30 (Fragment) |  | 16 | 3 | 0.82 | 0.905 | 0.861 | 0.873 | 1.013 | 1.062 | 0.877 | 0.156 |
| A2N0T6 | VH6DJ protein (Fragment) | VH6DJ | 12 | 1 | 0.9 | 0.865 | 0.772 | 1.098 | 0.943 | 1.041 | 0.823 | 0.039 |
| A2N0U1 | VH6DJ protein (Fragment) | VH6DJ | 12 | 1 | 1.035 | 0.974 | 0.912 | 1.272 | 0.94 | 1.024 | 0.902 | 0.406 |
| A2N2F4 | VK3 protein (Fragment) | VK3 | 5 | 1 | 1.262 | 2.296 | 1.454 | 1.238 | 1.243 | 1.212 | 1.357 | 0.3 |
| A2N7P4 | Immunoglobulin mu-chain D-J4-region (Fragment) | IGHM | 6 | 1 | 0.971 | 1.082 | 0.88 | 0.437 | 0.455 | 0.454 | 2.179 | 0.011 |
| A2NB44 | Cold agglutinin FS-2 H-chain (Fragment) | IGH@ | 5 | 1 | 0.95 | 0.943 | 1.008 | 1.07 | 0.987 | 1.22 | 0.885 | 0.2 |
| A2NB45 | Cold agglutinin FS-1 L-chain (Fragment) |  | 45 | 1 | 0.904 | 1.039 | 0.994 | 1.088 | 1.002 | 0.989 | 0.954 | 0.401 |
| A2NH53 | Immunogobulin kappa, VJ region (Fragment) |  | 1 | 1 | 0.635 | 0.727 | 0.934 | 0.807 | 1.169 | 1.02 | 0.766 | 0.166 |
| A2NKM7 | NANUC-2 heavy chain (Fragment) |  | 28 | 1 | 1.256 | 0.844 | 1.328 | 1.024 | 1.148 | 1.015 | 1.076 | 0.653 |
| A2NV54 | Precursor (AA -19 to 108) (Fragment) |  | 12 | 3 | 0.91 | 0.916 | 1.472 | 0.916 | 0.926 | 0.896 | 1.204 | 0.422 |
| A2NW98 | Rheumatoid factor light chain variable region (Fragment) |  | 1 | 1 | 1.355 | 1.587 | 1.361 | 1.095 | 0.926 | 1.021 | 1.414 | 0.014 |
| A2NYU7 | Heavy chain Fab (Fragment) |  | 2 | 1 | 1.073 | 0.944 | 1.233 | 0.369 | 0.367 | 0.403 | 2.853 | 0.013 |
| A2NYU8 | Heavy chain Fab (Fragment) |  | 1 | 1 | 0.757 | 0.71 | 1.169 | 0.591 | 0.577 | 0.635 | 1.461 | 0.196 |
| A2NYV1 | Heavy chain Fab (Fragment) |  | 7 | 1 | 0.956 | 0.709 | 0.793 | 1.973 | 1.141 | 0.925 | 0.608 | 0.237 |
| A4D0V4 | Capping protein (Actin filament) muscle Z-line, alpha 2, isoform CRA_b | CAPZA2 | 2 | 1 | 0.762 | 0.873 | 1.041 | 1.032 | 0.925 | 0.982 | 0.91 | 0.398 |
| A4D1W8 | Ependymin related protein 1 (Zebrafish), isoform CRA_b | UCC1 | 1 | 1 | 0.843 | 0.947 | 1.234 | 1.139 | 1.324 | 1.277 | 0.809 | 0.167 |
| A5PL27 | CP protein | CP | 178 | 29 | 1.053 | 1.042 | 0.972 | 0.965 | 1.061 | 0.843 | 1.069 | 0.409 |
| A5PL32 | APOL1 protein (Fragment) | APOL1 | 26 | 9 | 0.791 | 0.937 | 0.838 | 0.982 | 1.019 | 1.109 | 0.825 | 0.034 |
| A5PLK9 | Metalloendopeptidase | BMP1 | 13 | 9 | 0.904 | 0.854 | 0.852 | 1.016 | 0.988 | 1.047 | 0.855 | 0.004 |
| A8K061 | cDNA FLJ77880, highly similar to Homo sapiens angiopoietin-like 3, mRNA |  | 5 | 4 | 0.981 | 0.888 | 0.958 | 0.918 | 0.891 | 0.907 | 1.041 | 0.312 |
| A8K1K1 | cDNA FLJ76342, highly similar to Homo sapiens carnosine dipeptidase 1 (metallopeptidase M20 family)(CNDP1), mRNA |  | 17 | 7 | 0.868 | 0.789 | 1.077 | 0.795 | 1.463 | 1.097 | 0.815 | 0.406 |
| A8K2L4 | cDNA FLJ76079, highly similar to Homo sapiens lymphocyte-specific protein 1 (LSP1), mRNA |  | 2 | 2 | 1.099 | 1.135 | 0.919 | 1.16 | 1.148 | 1.076 | 0.932 | 0.372 |
| A8K2M5 | Lipoprotein lipase |  | 11 | 6 | 0.998 | 0.898 | 1.151 | 0.866 | 0.815 | 0.907 | 1.177 | 0.164 |
| A8K2T4 | cDNA FLJ78207, highly similar to Human complement protein component C7 mRNA |  | 55 | 21 | 0.877 | 1.01 | 0.836 | 0.957 | 1.113 | 1.24 | 0.823 | 0.127 |
| A8K3H0 | cDNA FLJ75548, highly similar to Homo sapiens microfibrillar associated protein 5 (MFAP5), mRNA |  | 3 | 1 | 1.017 | 1.031 | 1.24 | 1.043 | 1.111 | 0.79 | 1.116 | 0.404 |
| A8K3H8 | cDNA FLJ77680, highly similar to Homo sapiens protein phosphatase 2 (formerly 2A), regulatory subunit A (PR 65), alpha isoform (PPP2R1A), mRNA |  | 1 | 1 | 1.117 | 1.015 | 1.053 | 1.13 | 0.886 | 1.18 | 0.996 | 0.97 |
| A8K3I0 | cDNA FLJ78437, highly similar to Homo sapiens cartilage oligomeric matrix protein (COMP), mRNA |  | 11 | 5 | 1.202 | 1.144 | 0.965 | 1.15 | 1.136 | 0.956 | 1.021 | 0.823 |
| A8K486 | Peptidyl-prolyl cis-trans isomerase |  | 2 | 2 | 1.181 | 1.356 | 1.048 | 0.889 | 0.82 | 1.164 | 1.248 | 0.162 |
| A8K5T0 | cDNA FLJ75416, highly similar to Homo sapiens complement factor H (CFH), mRNA |  | 1366 | 1 | 1.119 | 0.982 | 1.164 | 1.049 | 1.115 | 1.071 | 1.009 | 0.876 |
| A8K7Q1 | cDNA FLJ77770, highly similar to Homo sapiens nucleobindin 1 (NUCB1), mRNA |  | 11 | 9 | 0.98 | 0.969 | 1.096 | 0.885 | 0.834 | 0.849 | 1.185 | 0.046 |
| A8K8U1 | cDNA FLJ77762, highly similar to Homo sapiens cullin-associated and neddylation-dissociated 1 (CAND1), mRNA |  | 1 | 1 | 0.909 | 0.853 | 0.791 | 1.235 | 1.066 | 1.161 | 0.737 | 0.009 |
| A8K9M5 | cDNA FLJ77947, highly similar to Human complement protein C8 beta subunit mRNA |  | 28 | 11 | 0.876 | 0.992 | 0.903 | 1.068 | 1.176 | 1.201 | 0.805 | 0.015 |
| A8KAP9 | cDNA FLJ78448, highly similar to Homo sapiens argininosuccinate synthetase (ASS), transcript variant 1, mRNA |  | 1 | 1 | 0.799 | 0.847 | 0.801 | 0.865 | 1.101 | 1.015 | 0.821 | 0.117 |
| B0AZL7 | cDNA, FLJ79457, highly similar to Insulin-like growth factor-binding proteincomplex acid labile chain |  | 16 | 11 | 0.815 | 0.839 | 0.866 | 1.065 | 1.046 | 1.102 | 0.785 | 0.001 |
| B0YIW2 | Apolipoprotein C-III variant 1 | APOC3 | 139 | 7 | 1.226 | 1.039 | 1.003 | 1.028 | 0.914 | 0.823 | 1.182 | 0.141 |
| B0YJC6 | Vitamin K-dependent protein Z variant 1 | PROZ | 49 | 13 | 1.006 | 0.874 | 0.913 | 0.936 | 0.867 | 0.803 | 1.071 | 0.322 |
| B1N7B6 | Cryocrystalglobulin CC1 heavy chain variable region (Fragment) |  | 20 | 2 | 1.16 | 1.129 | 1.806 | 0.99 | 1.12 | 0.888 | 1.365 | 0.235 |
| B1N7B8 | Cryocrystalglobulin CC1 kappa light chain variable region (Fragment) |  | 14 | 1 | 0.614 | 0.741 | 0.565 | 1.155 | 0.95 | 1.133 | 0.593 | 0.007 |
| B1N7B9 | Cryocrystalglobulin CC2 lambda light chain variable region (Fragment) |  | 8 | 2 | 0.923 | 1.289 | 1.134 | 0.969 | 1.188 | 1.09 | 1.031 | 0.805 |
| B2MUX6 | Insulin-like growth factor 2 | IGF2 | 9 | 3 | 1.007 | 1.041 | 0.977 | 0.999 | 1.045 | 1.046 | 0.979 | 0.426 |
| B2R4M6 | Protein S100 |  | 9 | 3 | 0.999 | 1.453 | 0.869 | 0.989 | 0.918 | 0.944 | 1.165 | 0.469 |
| B2R4R0 | Histone H4 | HIST1H4L | 7 | 3 | 1.154 | 1.078 | 1.122 | 1.19 | 1.18 | 1.127 | 0.959 | 0.183 |
| B2R5J8 | C-C motif chemokine |  | 11 | 2 | 1.208 | 1.171 | 1.086 | 0.852 | 0.86 | 0.802 | 1.379 | 0.005 |
| B2R5M3 | cDNA, FLJ92530, highly similar to Homo sapiens chromogranin B (secretogranin 1) (CHGB), mRNA |  | 2 | 1 | 1.037 | 0.869 | 0.986 | 1.193 | 1.237 | 1.143 | 0.809 | 0.026 |
| B2R6C0 | Glycerol-3-phosphate dehydrogenase [NAD(+)] |  | 1 | 1 | 0.952 | 1.04 | 0.979 | 1.1 | 1.019 | 0.995 | 0.954 | 0.31 |
| B2R6S5 | UMP-CMP kinase | CMPK | 1 | 1 | 0.808 | 0.964 | 0.713 | 0.847 | 0.911 | 0.934 | 0.923 | 0.453 |
| B2R6V9 | cDNA, FLJ93141, highly similar to Homo sapiens coagulation factor XIII, A1 polypeptide (F13A1), mRNA |  | 34 | 11 | 1.013 | 1.153 | 1.02 | 0.912 | 0.926 | 0.953 | 1.142 | 0.092 |
| B2R773 | cDNA, FLJ93312, highly similar to Homo sapiens adipose most abundant gene transcript 1 (APM1), mRNA |  | 15 | 4 | 1.124 | 0.996 | 1.223 | 0.925 | 1.093 | 0.916 | 1.14 | 0.193 |
| B2R7D2 | cDNA, FLJ93389, highly similar to Homo sapiens multiple inositol polyphosphate histidine phosphatase, 1 (MINPP1), mRNA |  | 2 | 2 | 0.959 | 1.053 | 1.097 | 1.078 | 1.182 | 1.196 | 0.9 | 0.104 |
| B2R7N9 | cDNA, FLJ93532, highly similar to Homo sapiens osteomodulin (OMD), mRNA |  | 7 | 2 | 0.998 | 0.981 | 0.833 | 1.288 | 1.157 | 1.093 | 0.795 | 0.036 |
| B2R7T2 | cDNA, FLJ93591, highly similar to Homo sapiens transforming growth factor, beta 2 (TGFB2), mRNA |  | 1 | 1 | 1.05 | 0.995 | 0.869 | 1.069 | 0.984 | 0.934 | 0.975 | 0.732 |
| B2R7Z6 | cDNA, FLJ93674 |  | 1 | 1 | 1.26 | 1.09 | 1.341 | 1.058 | 1.124 | 1.201 | 1.091 | 0.308 |
| B2R815 | cDNA, FLJ93695, highly similar to Homo sapiens serpin peptidase inhibitor, clade A (alpha-1 antiproteinase, antitrypsin), member 4 (SERPINA4), mRNA |  | 9 | 6 | 0.89 | 0.844 | 0.896 | 1.081 | 1.097 | 1.106 | 0.8 | 0.002 |
| B2R829 | cDNA, FLJ93711, highly similar to Homo sapiens myeloid cell nuclear differentiation antigen (MNDA), mRNA |  | 4 | 4 | 1.1 | 1.117 | 0.93 | 1.045 | 0.888 | 1.005 | 1.071 | 0.413 |
| B2R888 | Monocyte differentiation antigen CD14 |  | 33 | 10 | 0.802 | 1.142 | 0.766 | 1.043 | 1.054 | 1.035 | 0.865 | 0.36 |
| B2R8I2 | cDNA, FLJ93914, highly similar to Homo sapiens histidine-rich glycoprotein (HRG), mRNA |  | 101 | 2 | 0.948 | 0.934 | 0.833 | 1.139 | 1.219 | 1.206 | 0.762 | 0.005 |
| B2R950 | cDNA, FLJ94213, highly similar to Homo sapiens pregnancy-zone protein (PZP), mRNA |  | 94 | 18 | 1.146 | 1 | 1.042 | 0.803 | 0.872 | 0.764 | 1.307 | 0.012 |
| B2R983 | cDNA, FLJ94267, highly similar to Homo sapiens glutathione S-transferase omega 1 (GSTO1), mRNA |  | 1 | 1 | 0.996 | 1.013 | 0.876 | 1.21 | 1.005 | 1.101 | 0.87 | 0.129 |
| B2R9F2 | cDNA, FLJ94361, highly similar to Homo sapiens serine (or cysteine) proteinase inhibitor, clade A(alpha-1 antiproteinase, antitrypsin), member 6 (SERPINA6), mRNA |  | 1 | 1 | 0.936 | 0.988 | 1.066 | 1.2 | 1.213 | 0.97 | 0.884 | 0.236 |
| B2R9K6 | cDNA, FLJ94436, highly similar to Homo sapiens platelet derived growth factor C (PDGFC), mRNA |  | 5 | 4 | 1.183 | 1.256 | 1.188 | 0.971 | 1.009 | 1.155 | 1.157 | 0.084 |
| B2RAN2 | cDNA, FLJ95014, highly similar to Homo sapiens vanin 1 (VNN1), mRNA |  | 3 | 3 | 0.982 | 0.888 | 0.733 | 1.231 | 0.957 | 1.314 | 0.743 | 0.092 |
| B2RBW9 | cDNA, FLJ95746, highly similar to Homo sapiens inhibin, beta C (INHBC), mRNA |  | 8 | 4 | 0.877 | 0.956 | 0.893 | 0.995 | 0.952 | 1.019 | 0.919 | 0.064 |
| B2RDG0 | Proteasome subunit alpha type |  | 2 | 2 | 0.899 | 0.967 | 0.92 | 0.939 | 0.855 | 1.033 | 0.986 | 0.822 |
| B2RDY9 | Adenylyl cyclase-associated protein |  | 2 | 2 | 0.969 | 1.946 | 0.923 | 1.072 | 0.906 | 1.1 | 1.246 | 0.529 |
| B2RMS9 | Inter-alpha (Globulin) inhibitor H4 (Plasma Kallikrein-sensitive glycoprotein) | ITIH4 | 173 | 27 | 1.03 | 1.173 | 1.02 | 0.804 | 0.778 | 0.916 | 1.29 | 0.021 |
| B3KNB4 | cDNA FLJ14168 fis, clone NT2RP2001440, highly similar to 14-3-3 protein gamma |  | 11 | 2 | 0.989 | 1.024 | 0.953 | 1.052 | 0.96 | 1.008 | 0.983 | 0.628 |
| B3KNX0 | cDNA FLJ30621 fis, clone CTONG2001681, highly similar to Complement C1s subcomponent (EC 3.4.21.42) |  | 83 | 19 | 1.027 | 1.052 | 1.123 | 0.896 | 0.817 | 0.846 | 1.251 | 0.005 |
| B3KQF5 | cDNA FLJ90381 fis, clone NT2RP2005035, highly similar to Calumenin |  | 18 | 1 | 0.901 | 0.932 | 0.772 | 1.111 | 1.069 | 1.094 | 0.796 | 0.038 |
| B3KQT8 | cDNA PSEC0172 fis, clone PLACE1011835, highly similar to Procollagen C-endopeptidase enhancer 2 |  | 5 | 5 | 0.949 | 0.942 | 0.97 | 1.002 | 1.119 | 1.024 | 0.91 | 0.112 |
| B3KS79 | cDNA FLJ35730 fis, clone TESTI2003131, highly similar to ALPHA-1-ANTICHYMOTRYPSIN |  | 26 | 11 | 1.069 | 1.168 | 1.015 | 0.943 | 0.952 | 0.878 | 1.173 | 0.051 |
| B3KUE5 | Phospholipid transfer protein, isoform CRA_c | PLTP | 9 | 3 | 0.882 | 0.923 | 0.918 | 1.117 | 1.086 | 1.073 | 0.831 | 0.001 |
| B3KUI5 | Hyaluronidase |  | 8 | 4 | 0.943 | 1.018 | 0.92 | 0.98 | 1.058 | 0.965 | 0.959 | 0.38 |
| B3VMW0 | Lactoferrin |  | 154 | 3 | 0.988 | 0.947 | 1.051 | 1.078 | 1.037 | 1.099 | 0.929 | 0.113 |
| B4DEA7 | cDNA FLJ58131, highly similar to Secretogranin-1 |  | 2 | 1 | 1.203 | 1.084 | 1.066 | 1.126 | 1.052 | 1.11 | 1.02 | 0.689 |
| B4DI63 | cDNA FLJ59205, highly similar to Mimecan |  | 2 | 2 | 1.074 | 1.058 | 1.111 | 0.892 | 0.909 | 0.921 | 1.192 | 0.002 |
| B4DID6 | cDNA FLJ52545, highly similar to Dickkopf-related protein 3 |  | 7 | 2 | 0.949 | 0.999 | 0.993 | 1.076 | 1.056 | 1.076 | 0.917 | 0.017 |
| B4DJ30 | cDNA FLJ61290, highly similar to Neutral alpha-glucosidase AB |  | 17 | 13 | 0.927 | 0.963 | 1.162 | 0.893 | 0.942 | 0.924 | 1.106 | 0.311 |
| B4DJQ5 | cDNA FLJ59211, highly similar to Glucosidase 2 subunit beta |  | 18 | 7 | 1.019 | 0.925 | 0.945 | 0.905 | 0.923 | 0.852 | 1.078 | 0.127 |
| B4DJV9 | cDNA FLJ60607, highly similar to Acyl-protein thioesterase 1 (EC 3.1.2.-) |  | 2 | 2 | 1.1 | 0.893 | 3.338 | 1.152 | 1.292 | 1.131 | 1.491 | 0.533 |
| B4DKV4 | cDNA FLJ60647, highly similar to Keratin, type II cytoskeletal 6B |  | 112 | 1 | 0.743 | 0.97 | 3.136 | 0.282 | 0.355 | 0.541 | 4.117 | 0.249 |
| B4DM85 | Kinesin-like protein |  | 2 | 2 | 1.028 | 1.016 | 1.536 | 0.821 | 0.708 | 0.764 | 1.561 | 0.125 |
| B4DMN1 | cDNA FLJ61136, highly similar to Ras-related protein Rab-11A |  | 3 | 3 | 0.778 | 0.792 | 1.017 | 0.784 | 0.717 | 0.97 | 1.047 | 0.74 |
| B4DPQ0 | cDNA FLJ54471, highly similar to Complement C1r subcomponent (EC 3.4.21.41) | C1R | 96 | 20 | 1.057 | 1.116 | 1.124 | 0.821 | 0.796 | 0.8 | 1.364 | 0.002 |
| B4DPQ3 | cDNA FLJ51034, highly similar to Vitamin K-dependent protein C (EC 3.4.21.69) |  | 67 | 12 | 1.033 | 0.903 | 0.929 | 1.029 | 1.021 | 0.993 | 0.941 | 0.268 |
| B4DR52 | Histone H2B |  | 7 | 2 | 1.087 | 1.192 | 0.864 | 1.196 | 1.315 | 1.144 | 0.86 | 0.216 |
| B4DR57 | cDNA FLJ60818, highly similar to Complement C3 |  | 87 | 1 | 0.899 | 0.983 | 0.924 | 1.174 | 1.266 | 0.994 | 0.817 | 0.11 |
| B4DT31 | cDNA FLJ53425, highly similar to Far upstream element-binding protein 1 |  | 1 | 1 | 1.074 | 1.036 | 1.053 | 0.659 | 0.554 | 0.541 | 1.804 | 0.004 |
| B4DUV1 | Fibulin-1 |  | 255 | 6 | 0.981 | 1.043 | 0.977 | 1.048 | 1.001 | 0.981 | 0.99 | 0.756 |
| B4DVA7 | Beta-hexosaminidase |  | 2 | 2 | 0.851 | 0.899 | 0.991 | 1.055 | 1.042 | 1.136 | 0.848 | 0.036 |
| B4DWA6 | cDNA FLJ60094, highly similar to F-actin capping protein subunit beta |  | 1 | 1 | 0.73 | 0.924 | 0.893 | 0.952 | 0.91 | 1.003 | 0.889 | 0.214 |
| B4E1B2 | cDNA FLJ53691, highly similar to Serotransferrin |  | 61 | 22 | 1.07 | 0.784 | 0.68 | 1.191 | 1.033 | 0.797 | 0.839 | 0.377 |
| B4E1U9 | cDNA FLJ54776, highly similar to Cell division control protein 42 homolog |  | 1 | 1 | 1.252 | 1.243 | 1.499 | 1.149 | 1.302 | 1.114 | 1.121 | 0.24 |
| B4E1Z4 | cDNA FLJ55673, highly similar to Complement factor B (EC 3.4.21.47) |  | 82 | 27 | 1.029 | 1.158 | 0.931 | 1.047 | 1.145 | 1.071 | 0.956 | 0.553 |
| B4E3D4 | cDNA FLJ56293, highly similar to Transmembrane glycoprotein NMB |  | 1 | 1 | 0.97 | 0.993 | 1.031 | 1.081 | 1.085 | 1.071 | 0.925 | 0.038 |
| B6EDE2 | Epididymis luminal protein 180 (Fragment) | HEL180 | 13 | 1 | 1.133 | 0.93 | 0.901 | 0.849 | 0.905 | 0.934 | 1.102 | 0.336 |
| B7Z1F8 | cDNA FLJ53025, highly similar to Complement C4-B |  | 538 | 2 | 0.77 | 0.897 | 0.677 | 1.09 | 1.16 | 1.09 | 0.702 | 0.024 |
| B7Z539 | cDNA FLJ56954, highly similar to Inter-alpha-trypsin inhibitor heavy chain H1 |  | 394 | 12 | 0.956 | 0.891 | 0.922 | 1.09 | 1.073 | 1.117 | 0.844 | 0.003 |
| B7Z6C2 | cDNA FLJ50663, highly similar to Phosphoglucomutase-1 (EC 5.4.2.2) |  | 1 | 1 | 0.756 | 0.818 | 0.87 | 1.133 | 0.994 | 1.137 | 0.749 | 0.012 |
| B7Z6S9 | Glucosylceramidase |  | 1 | 1 | 0.915 | 0.939 | 1.031 | 0.959 | 1.067 | 1.073 | 0.931 | 0.235 |
| B7Z6Z4 | cDNA FLJ56329, highly similar to Myosin light polypeptide 6 | MYL6 | 2 | 2 | 1.225 | 1.24 | 1.184 | 0.902 | 0.75 | 0.753 | 1.517 | 0.009 |
| B7Z809 | cDNA FLJ56016, highly similar to C-1-tetrahydrofolate synthase, cytoplasmic |  | 1 | 1 | 1.368 | 1.032 | 1.273 | 1.155 | 1.21 | 1.029 | 1.082 | 0.475 |
| B7Z8B6 | cDNA FLJ54395, highly similar to Inter-alpha-trypsin inhibitor heavy chain H1 |  | 103 | 2 | 1.047 | 0.727 | 1.807 | 0.385 | 0.448 | 0.297 | 3.168 | 0.123 |
| B7Z8Q2 | cDNA FLJ55606, highly similar to Alpha-2-HS-glycoprotein |  | 112 | 9 | 0.881 | 1.004 | 0.776 | 1.223 | 1.163 | 1.158 | 0.751 | 0.037 |
| B7Z9B8 | cDNA FLJ56912, highly similar to Fibulin-2 |  | 3 | 3 | 1.064 | 1.078 | 1.167 | 1.076 | 1.012 | 0.921 | 1.1 | 0.154 |
| B8ZX62 | Plasminogen activator | PLAT | 3 | 3 | 1.091 | 1.068 | 0.955 | 1.071 | 0.998 | 1.033 | 1.004 | 0.941 |
| C0JYY2 | Apolipoprotein B (Including Ag(X) antigen), isoform CRA_a | APOB | 753 | 135 | 0.824 | 0.997 | 0.924 | 1.032 | 1.041 | 0.962 | 0.904 | 0.184 |
| C9JC84 | Fibrinogen gamma chain | FGG | 6 | 5 | 0.927 | 1.696 | 1.574 | 0.448 | 0.422 | 0.43 | 3.231 | 0.056 |
| C9JD84 | Latent-transforming growth factor beta-binding protein 1 | LTBP1 | 97 | 2 | 0.867 | 0.9 | 0.915 | 1.219 | 1.107 | 1.181 | 0.765 | 0.006 |
| C9JIF9 | Acylamino-acid-releasing enzyme | APEH | 8 | 7 | 1.257 | 1.007 | 0.964 | 1.121 | 0.967 | 1.161 | 0.994 | 0.954 |
| D0PNI1 | Epididymis luminal protein 4 | YWHAZ | 11 | 3 | 1.057 | 1.195 | 1.189 | 1.02 | 1.035 | 1.034 | 1.114 | 0.119 |
| D3DNN4 | Carboxylic ester hydrolase | BCHE | 8 | 5 | 0.866 | 0.895 | 0.818 | 1.159 | 1.071 | 1.019 | 0.794 | 0.016 |
| D3DQH8 | Secreted protein, acidic, cysteine-rich (Osteonectin), isoform CRA_a | SPARC | 17 | 8 | 0.889 | 0.932 | 0.732 | 0.808 | 0.74 | 0.826 | 1.076 | 0.441 |
| D3DQX7 | Serum amyloid A protein | SAA1 | 15 | 2 | 1.212 | 1.131 | 1.097 | 0.614 | 0.531 | 0.55 | 2.028 | 0 |
| D3DSQ1 | N-acylsphingosine amidohydrolase (Acid ceramidase) 1, isoform CRA_c | ASAH1 | 1 | 1 | 0.895 | 0.965 | 1.101 | 1.191 | 1.129 | 1.177 | 0.847 | 0.087 |
| D6RF35 | Vitamin D-binding protein | GC | 53 | 2 | 1.378 | 1.046 | 1.006 | 0.875 | 1.077 | 1.341 | 1.042 | 0.811 |
| D6W5L6 | Pulmonary surfactant-associated protein B | SFTPB | 1 | 1 | 1.241 | 1.003 | 1.074 | 1.005 | 0.956 | 0.846 | 1.181 | 0.126 |
| D9ZGF2 | Collagen, type VI, alpha 3 | COL6A3 | 8 | 8 | 0.89 | 1.25 | 1.025 | 0.958 | 0.932 | 0.876 | 1.144 | 0.333 |
| D9ZGG2 | Vitronectin | VTN | 2228 | 18 | 1.019 | 1.007 | 1.078 | 0.983 | 1 | 1.022 | 1.033 | 0.275 |
| E2RVJ0 | Anion exchange protein | SLC4A1 | 1 | 1 | 0.695 | 0.727 | 0.662 | 1.153 | 1.036 | 1.093 | 0.635 | 0.002 |
| E5RJR5 | S-phase kinase-associated protein 1 | SKP1 | 1 | 1 | 1.061 | 0.973 | 0.866 | 1.092 | 1.092 | 0.974 | 0.918 | 0.287 |
| E7EMB3 | Calmodulin-2 | CALM2 | 20 | 5 | 1.279 | 1.125 | 1.116 | 0.875 | 0.758 | 0.741 | 1.482 | 0.006 |
| E7EQ64 | Trypsin-1 | PRSS1 | 22 | 2 | 1.098 | 1.041 | 0.882 | 1.157 | 1.103 | 1.069 | 0.907 | 0.249 |
| E9PK25 | Cofilin-1 | CFL1 | 10 | 4 | 1.018 | 0.953 | 0.828 | 0.986 | 0.902 | 0.865 | 1.017 | 0.825 |
| F2RM37 | Coagulation factor IX | F9 | 57 | 15 | 0.956 | 1.015 | 0.936 | 0.956 | 0.944 | 0.929 | 1.028 | 0.387 |
| F5H423 | Uncharacterized protein |  | 3 | 2 | 1.002 | 0.933 | 1.239 | 0.67 | 0.639 | 0.723 | 1.562 | 0.046 |
| F8W031 | Uncharacterized protein (Fragment) |  | 1 | 1 | 1.019 | 0.988 | 1 | 0.411 | 0.401 | 0.377 | 2.528 | 0 |
| G1FM90 | Anti-Influenza A hemagglutinin heavy chain variable region (Fragment) |  | 9 | 2 | 1.525 | 1.089 | 1.378 | 0.853 | 0.704 | 0.911 | 1.617 | 0.04 |
| G3V2W1 | Protein Z-dependent protease inhibitor | SERPINA10 | 19 | 8 | 0.854 | 0.995 | 1.01 | 0.907 | 1.027 | 0.91 | 1.005 | 0.941 |
| G3V5Z7 | Proteasome subunit alpha type | PSMA6 | 1 | 1 | 0.914 | 0.846 | 0.799 | 0.873 | 0.895 | 0.883 | 0.965 | 0.456 |
| G3XAI2 | Laminin subunit beta-1 | LAMB1 | 3 | 3 | 0.953 | 0.861 | 1.128 | 1.118 | 1.151 | 1.084 | 0.878 | 0.218 |
| G3XAK1 | Hepatocyte growth factor-like protein | MST1 | 29 | 12 | 0.859 | 0.723 | 0.868 | 0.954 | 1.316 | 1.025 | 0.743 | 0.111 |
| G4XXL9 | Cytochrome c, somatic, isoform CRA_a | CYCS | 1 | 1 | 0.832 | 1.093 | 0.628 | 1.063 | 1.812 | 0.989 | 0.661 | 0.236 |
| H0YAC1 | Plasma kallikrein (Fragment) | KLKB1 | 37 | 19 | 1.093 | 0.902 | 0.892 | 1.098 | 1.069 | 1.019 | 0.906 | 0.262 |
| H0YDW8 | Granulysin (Fragment) | GNLY | 1 | 1 | 1.128 | 1.322 | 1.163 | 0.858 | 0.792 | 0.898 | 1.418 | 0.013 |
| H3BMJ4 | Golgin subfamily A member 6-like protein 2 | GOLGA6L2 | 1 | 1 | 0.055 | 0.095 | 0.09 | 1.659 | 4.232 | 1.801 | 0.031 | 0.097 |
| H6VRG1 | Keratin 1 | KRT1 | 403 | 43 | 0.69 | 0.903 | 1.026 | 1.016 | 1.058 | 1.277 | 0.782 | 0.13 |
| H7BY58 | Protein-L-isoaspartate O-methyltransferase | PCMT1 | 2 | 2 | 0.995 | 1.04 | 0.963 | 1.066 | 0.837 | 1.061 | 1.011 | 0.897 |
| H7BY64 | ZNF511-PRAP1 readthrough (Fragment) | ZNF511-PRAP1 | 4 | 1 | 1.148 | 1.185 | 1.022 | 0.978 | 0.846 | 1.134 | 1.134 | 0.259 |
| I3L504 | Eukaryotic translation initiation factor 5A-1 | EIF5A | 2 | 2 | 1.372 | 0.907 | 4.317 | 1.084 | 1.556 | 2.103 | 1.39 | 0.627 |
| J3KNB4 | Cathelicidin antimicrobial peptide | CAMP | 6 | 5 | 0.975 | 1.222 | 1.119 | 0.907 | 0.953 | 0.894 | 1.204 | 0.113 |
| J3KQ66 | Reelin | RELN | 5 | 5 | 0.912 | 1.73 | 0.817 | 0.903 | 0.898 | 0.835 | 1.312 | 0.444 |
| J3KQE5 | GTP-binding nuclear protein Ran (Fragment) | RAN | 6 | 3 | 1.177 | 0.883 | 1.035 | 1.069 | 1.045 | 1.137 | 0.952 | 0.61 |
| J3QRS3 | Myosin regulatory light chain 12A | MYL12A | 2 | 1 | 1.125 | 1.184 | 1.179 | 0.948 | 0.857 | 0.852 | 1.313 | 0.003 |
| K7ER74 | APOC4-APOC2 readthrough (NMD candidate) | APOC4-APOC2 | 49 | 4 | 1.072 | 1.094 | 0.913 | 1.172 | 0.844 | 0.712 | 1.128 | 0.495 |
| K7ES70 | Microfibril-associated glycoprotein 4 | MFAP4 | 2 | 1 | 1.028 | 1.097 | 1.25 | 0.951 | 1.031 | 0.79 | 1.218 | 0.106 |
| K9JA46 | Epididymis luminal secretory protein 52 | EL52 | 7 | 1 | 0.914 | 0.972 | 0.852 | 1.027 | 0.994 | 0.881 | 0.943 | 0.389 |
| L8E853 | von Willebrand factor | VWF | 94 | 46 | 1.046 | 0.983 | 1.022 | 0.972 | 0.94 | 1.007 | 1.045 | 0.174 |
| M1LAK4 | Olfactomedin-like 3, isoform CRA_b | OLFML3 | 3 | 3 | 0.908 | 0.879 | 0.899 | 1.11 | 1.087 | 1.094 | 0.816 | 0 |
| M1VKI3 | Tyrosine-protein kinase receptor | SDC4-ROS1_S4;R32 | 1 | 1 | 0.98 | 0.97 | 0.905 | 1.075 | 1.055 | 1.116 | 0.88 | 0.014 |
| O00187 | Mannan-binding lectin serine protease 2 | MASP2 | 42 | 14 | 0.979 | 0.983 | 1.038 | 0.953 | 0.892 | 0.851 | 1.113 | 0.055 |
| O00194 | Ras-related protein Rab-27B | RAB27B | 1 | 1 | 0.911 | 0.909 | 1.221 | 0.958 | 0.906 | 0.881 | 1.108 | 0.444 |
| O00299 | Chloride intracellular channel protein 1 | CLIC1 | 6 | 4 | 1.18 | 1.132 | 1.139 | 1.05 | 0.892 | 0.964 | 1.187 | 0.047 |
| O00391 | Sulfhydryl oxidase 1 | QSOX1 | 33 | 12 | 0.952 | 0.929 | 0.99 | 0.916 | 0.926 | 0.942 | 1.031 | 0.238 |
| O00461 | Golgi integral membrane protein 4 | GOLIM4 | 4 | 2 | 1 | 0.943 | 1.063 | 0.977 | 0.896 | 1.13 | 1.001 | 0.994 |
| O00602 | Ficolin-1 | FCN1 | 10 | 5 | 0.995 | 1.344 | 1.209 | 0.954 | 0.926 | 0.986 | 1.238 | 0.151 |
| O14786 | Neuropilin-1 | NRP1 | 5 | 4 | 0.957 | 0.919 | 0.891 | 1.03 | 1.011 | 1.036 | 0.899 | 0.022 |
| O14960 | Leukocyte cell-derived chemotaxin-2 | LECT2 | 6 | 1 | 1.102 | 1.019 | 0.773 | 1.087 | 1.15 | 1.256 | 0.828 | 0.17 |
| O15467 | C-C motif chemokine 16 | CCL16 | 2 | 2 | 1.04 | 0.988 | 0.799 | 0.596 | 0.569 | 0.766 | 1.464 | 0.037 |
| O43866 | CD5 antigen-like | CD5L | 117 | 15 | 0.957 | 1.007 | 0.872 | 1.112 | 1.014 | 1.069 | 0.887 | 0.075 |
| O60234 | Glia maturation factor gamma | GMFG | 5 | 3 | 0.962 | 1.085 | 0.892 | 0.923 | 0.91 | 0.944 | 1.058 | 0.442 |
| O60462 | Neuropilin-2 | NRP2 | 19 | 13 | 0.913 | 0.879 | 0.909 | 0.886 | 0.904 | 0.984 | 0.974 | 0.516 |
| O60568 | Procollagen-lysine,2-oxoglutarate 5-dioxygenase 3 | PLOD3 | 1 | 1 | 0.983 | 1.123 | 1.156 | 1.06 | 0.874 | 1.068 | 1.087 | 0.356 |
| O75356 | Ectonucleoside triphosphate diphosphohydrolase 5 | ENTPD5 | 3 | 3 | 0.864 | 0.929 | 0.885 | 1.012 | 1.034 | 1.076 | 0.858 | 0.005 |
| O75558 | Syntaxin-11 | STX11 | 1 | 1 | 1.03 | 1.042 | 1.106 | 0.877 | 0.84 | 0.816 | 1.255 | 0.003 |
| O75563 | Src kinase-associated phosphoprotein 2 | SKAP2 | 1 | 1 | 1.026 | 1.121 | 1.136 | 0.999 | 0.963 | 0.988 | 1.113 | 0.074 |
| O75594 | Peptidoglycan recognition protein 1 | PGLYRP1 | 2 | 1 | 0.554 | 0.736 | 0.393 | 1.025 | 1.022 | 1.116 | 0.532 | 0.029 |
| O75636 | Ficolin-3 | FCN3 | 97 | 8 | 0.892 | 1.057 | 0.937 | 0.973 | 0.96 | 0.888 | 1.023 | 0.727 |
| O75787 | Renin receptor | ATP6AP2 | 1 | 1 | 1.018 | 1.117 | 1.054 | 0.894 | 0.887 | 0.836 | 1.219 | 0.008 |
| O76076 | WNT1-inducible-signaling pathway protein 2 | WISP2 | 12 | 7 | 1.002 | 1.064 | 0.977 | 1.019 | 0.93 | 1.083 | 1.003 | 0.955 |
| O94769 | Extracellular matrix protein 2 | ECM2 | 7 | 5 | 1.001 | 0.819 | 1.017 | 1.228 | 1.146 | 1.087 | 0.82 | 0.061 |
| O94985 | Calsyntenin-1 | CLSTN1 | 3 | 3 | 0.861 | 0.825 | 0.797 | 1.12 | 1.194 | 1.244 | 0.698 | 0.003 |
| O95428 | Papilin | PAPLN | 7 | 5 | 0.947 | 0.988 | 0.8 | 0.921 | 0.931 | 0.897 | 0.995 | 0.946 |
| O95445 | Apolipoprotein M | APOM | 15 | 4 | 1.052 | 1.029 | 1.054 | 1.007 | 1.02 | 0.978 | 1.043 | 0.054 |
| P00325 | Alcohol dehydrogenase 1B | ADH1B | 2 | 1 | 0.866 | 0.919 | 0.784 | 0.868 | 0.931 | 0.821 | 0.981 | 0.757 |
| P00338 | L-lactate dehydrogenase A chain | LDHA | 6 | 3 | 0.895 | 1.093 | 0.977 | 0.81 | 1.05 | 0.942 | 1.058 | 0.579 |
| P00451 | Coagulation factor VIII | F8 | 12 | 9 | 1.017 | 0.977 | 1.082 | 0.879 | 0.889 | 0.89 | 1.157 | 0.044 |
| P00734 | Prothrombin | F2 | 2462 | 40 | 1.055 | 0.962 | 0.971 | 1.001 | 0.983 | 0.975 | 1.01 | 0.779 |
| P00738 | Haptoglobin | HP | 84 | 8 | 1.04 | 0.897 | 1.08 | 0.949 | 0.823 | 0.888 | 1.135 | 0.157 |
| P00739 | Haptoglobin-related protein | HPR | 85 | 6 | 0.906 | 0.857 | 1.281 | 1.064 | 0.988 | 1.022 | 0.99 | 0.949 |
| P00747 | Plasminogen | PLG | 299 | 42 | 1.014 | 0.989 | 0.943 | 0.993 | 1.091 | 1.046 | 0.941 | 0.16 |
| P01008 | Antithrombin-III | SERPINC1 | 1487 | 36 | 1.05 | 0.959 | 0.984 | 1.038 | 1.065 | 1.034 | 0.954 | 0.211 |
| P01009 | Alpha-1-antitrypsin | SERPINA1 | 214 | 22 | 0.924 | 0.982 | 0.912 | 0.816 | 1.762 | 0.865 | 0.818 | 0.568 |
| P01023 | Alpha-2-macroglobulin | A2M | 133 | 25 | 1.005 | 0.984 | 0.908 | 0.992 | 0.992 | 0.918 | 0.998 | 0.96 |
| P01024 | Complement C3 | C3 | 3582 | 119 | 0.984 | 1.017 | 0.99 | 1.116 | 1.017 | 1.017 | 0.949 | 0.242 |
| P01031 | Complement C5 | C5 | 212 | 50 | 0.922 | 0.992 | 0.924 | 0.89 | 1.489 | 1.074 | 0.822 | 0.367 |
| P01034 | Cystatin-C | CST3 | 1 | 1 | 1.095 | 1.047 | 1.042 | 1.006 | 1.153 | 0.896 | 1.042 | 0.627 |
| P01127 | Platelet-derived growth factor subunit B | PDGFB | 15 | 7 | 0.893 | 1.118 | 1.013 | 1.021 | 1.01 | 1.039 | 0.985 | 0.837 |
| P01133 | Pro-epidermal growth factor | EGF | 1 | 1 | 0.811 | 0.831 | 0.874 | 1.112 | 1.153 | 1.251 | 0.716 | 0.007 |
| P01137 | Transforming growth factor beta-1 | TGFB1 | 12 | 6 | 0.994 | 1.031 | 0.949 | 0.935 | 0.895 | 0.938 | 1.074 | 0.083 |
| P01591 | Immunoglobulin J chain | JCHAIN | 58 | 8 | 1.091 | 1.072 | 1.099 | 1.079 | 1.024 | 1.043 | 1.037 | 0.123 |
| P01601 | Immunoglobulin kappa variable 1D-16 | IGKV1D-16 | 3 | 1 | 0.872 | 1.102 | 0.979 | 0.901 | 1.932 | 0.95 | 0.781 | 0.498 |
| P01624 | Immunoglobulin kappa variable 3-15 | IGKV3-15 | 11 | 1 | 0.923 | 0.888 | 0.971 | 0.775 | 0.843 | 0.857 | 1.123 | 0.044 |
| P01704 | Immunoglobulin lambda variable 2-14 | IGLV2-14 | 1 | 1 | 1.001 | 0.638 | 1.516 | 0.983 | 0.875 | 1.104 | 1.065 | 0.828 |
| P01705 | Immunoglobulin lambda variable 2-23 | IGLV2-23 | 1 | 1 | 0.944 | 0.941 | 1.945 | 0.361 | 0.248 | 0.293 | 4.252 | 0.098 |
| P01706 | Immunoglobulin lambda variable 2-11 | IGLV2-11 | 7 | 1 | 1.132 | 1.353 | 2.109 | 0.581 | 0.538 | 0.532 | 2.782 | 0.08 |
| P01766 | Immunoglobulin heavy variable 3-13 | IGHV3-13 | 10 | 1 | 0.898 | 0.901 | 1.46 | 0.847 | 1.199 | 0.765 | 1.16 | 0.553 |
| P01833 | Polymeric immunoglobulin receptor | PIGR | 12 | 9 | 0.871 | 1.054 | 0.975 | 0.974 | 0.859 | 0.961 | 1.038 | 0.614 |
| P01871 | Immunoglobulin heavy constant mu | IGHM | 1133 | 3 | 1.033 | 1.063 | 1.166 | 1.016 | 0.951 | 1.04 | 1.085 | 0.164 |
| P02042 | Hemoglobin subunit delta | HBD | 32 | 2 | 1.316 | 1.613 | 0.774 | 1.575 | 0.9 | 1.111 | 1.033 | 0.907 |
| P02452 | Collagen alpha-1(I) chain | COL1A1 | 1 | 1 | 0.731 | 0.81 | 0.74 | 0.885 | 1.024 | 0.782 | 0.848 | 0.181 |
| P02511 | Alpha-crystallin B chain | CRYAB | 4 | 1 | 0.844 | 0.833 | 0.794 | 1.008 | 0.945 | 1.043 | 0.825 | 0.012 |
| P02533 | Keratin, type I cytoskeletal 14 | KRT14 | 129 | 6 | 0.531 | 0.644 | 0.816 | 1.056 | 1.09 | 2.355 | 0.442 | 0.186 |
| P02545 | Prelamin-A/C | LMNA | 1 | 1 | 1.011 | 0.975 | 0.907 | 1.129 | 1.213 | 1.208 | 0.815 | 0.006 |
| P02549 | Spectrin alpha chain, erythrocytic 1 | SPTA1 | 5 | 5 | 0.973 | 1.134 | 0.899 | 1.163 | 0.943 | 1.186 | 0.913 | 0.414 |
| P02647 | Apolipoprotein A-I | APOA1 | 1475 | 34 | 1.024 | 1.043 | 1.002 | 0.978 | 1.027 | 1.035 | 1.01 | 0.677 |
| P02649 | Apolipoprotein E | APOE | 296 | 29 | 0.989 | 1.109 | 0.996 | 0.808 | 0.781 | 0.767 | 1.314 | 0.017 |
| P02654 | Apolipoprotein C-I | APOC1 | 81 | 8 | 1.004 | 1.022 | 0.903 | 1.015 | 0.968 | 1.091 | 0.953 | 0.399 |
| P02671 | Fibrinogen alpha chain | FGA | 35 | 13 | 0.95 | 1.454 | 1.224 | 0.838 | 0.757 | 0.823 | 1.5 | 0.105 |
| P02675 | Fibrinogen beta chain | FGB | 10 | 8 | 0.96 | 1.999 | 1.441 | 0.488 | 0.383 | 0.452 | 3.327 | 0.075 |
| P02741 | C-reactive protein | CRP | 80 | 7 | 1.191 | 1.576 | 1.393 | 0.419 | 0.222 | 0.302 | 4.41 | 0.003 |
| P02743 | Serum amyloid P-component | APCS | 145 | 8 | 1.035 | 1.094 | 1.201 | 1.004 | 0.916 | 0.855 | 1.2 | 0.047 |
| P02747 | Complement C1q subcomponent subunit C | C1QC | 91 | 7 | 1.013 | 1.084 | 1.142 | 0.876 | 0.896 | 0.997 | 1.169 | 0.042 |
| P02750 | Leucine-rich alpha-2-glycoprotein | LRG1 | 2 | 2 | 0.832 | 0.848 | 0.731 | 0.948 | 1.043 | 0.9 | 0.834 | 0.047 |
| P02753 | Retinol-binding protein 4 | RBP4 | 14 | 6 | 1.236 | 0.898 | 1.129 | 0.897 | 0.929 | 0.947 | 1.177 | 0.242 |
| P02760 | Protein AMBP | AMBP | 163 | 9 | 0.986 | 0.923 | 1.027 | 1.027 | 1.031 | 1.03 | 0.951 | 0.238 |
| P02768 | Serum albumin | ALB | 656 | 46 | 1.192 | 0.878 | 0.831 | 1.197 | 1.066 | 0.959 | 0.9 | 0.473 |
| P02775 | Platelet basic protein | PPBP | 27 | 4 | 1.018 | 1.037 | 0.815 | 1.053 | 1.21 | 1.136 | 0.845 | 0.117 |
| P02776 | Platelet factor 4 | PF4 | 378 | 8 | 0.955 | 1.246 | 0.985 | 1.245 | 1.312 | 1.101 | 0.871 | 0.241 |
| P02790 | Hemopexin | HPX | 15 | 9 | 1.12 | 0.879 | 0.956 | 0.956 | 0.864 | 0.831 | 1.115 | 0.296 |
| P03951 | Coagulation factor XI | F11 | 11 | 7 | 0.996 | 1.155 | 0.926 | 1.042 | 1.055 | 0.958 | 1.007 | 0.926 |
| P03973 | Antileukoproteinase | SLPI | 29 | 11 | 1.096 | 1.064 | 1.089 | 0.661 | 0.689 | 0.703 | 1.583 | 0 |
| P04003 | C4b-binding protein alpha chain | C4BPA | 783 | 27 | 1.016 | 1.019 | 0.913 | 0.97 | 0.988 | 0.951 | 1.014 | 0.743 |
| P04040 | Catalase | CAT | 3 | 2 | 0.918 | 0.944 | 1.125 | 1.123 | 0.921 | 1.18 | 0.927 | 0.484 |
| P04066 | Tissue alpha-L-fucosidase | FUCA1 | 1 | 1 | 0.906 | 1.021 | 0.673 | 1.098 | 0.946 | 1.059 | 0.838 | 0.24 |
| P04196 | Histidine-rich glycoprotein | HRG | 91 | 1 | 0.845 | 1.066 | 0.781 | 0.864 | 0.924 | 1.165 | 0.911 | 0.528 |
| P04211 | Immunoglobulin lambda variable 7-43 | IGLV7-43 | 14 | 1 | 1.439 | 0.749 | 1.537 | 0.777 | 1.371 | 0.759 | 1.281 | 0.443 |
| P04217 | Alpha-1B-glycoprotein | A1BG | 17 | 7 | 0.95 | 0.941 | 0.844 | 1.004 | 0.914 | 0.91 | 0.967 | 0.538 |
| P04259 | Keratin, type II cytoskeletal 6B | KRT6B | 150 | 1 | 0.447 | 0.483 | 0.675 | 0.995 | 1.079 | 3.045 | 0.314 | 0.221 |
| P04278 | Sex hormone-binding globulin | SHBG | 3 | 3 | 0.829 | 0.959 | 0.922 | 0.915 | 1.191 | 1.158 | 0.83 | 0.155 |
| P04406 | Glyceraldehyde-3-phosphate dehydrogenase | GAPDH | 20 | 9 | 1.268 | 1.099 | 1.005 | 0.952 | 0.991 | 1.058 | 1.124 | 0.246 |
| P04430 | Immunoglobulin kappa variable 1-16 | IGKV1-16 | 2 | 2 | 1.04 | 0.895 | 0.796 | 0.949 | 0.915 | 0.874 | 0.998 | 0.98 |
| P05019 | Insulin-like growth factor I | IGF1 | 3 | 1 | 1.021 | 0.939 | 0.912 | 1.184 | 1.308 | 1.476 | 0.724 | 0.036 |
| P05089 | Arginase-1 | ARG1 | 3 | 2 | 0.941 | 0.877 | 1.05 | 0.96 | 0.981 | 1.111 | 0.94 | 0.427 |
| P05090 | Apolipoprotein D | APOD | 266 | 14 | 1.074 | 1.01 | 1.113 | 0.931 | 0.937 | 0.862 | 1.171 | 0.017 |
| P05109 | Protein S100-A8 | S100A8 | 3 | 2 | 0.831 | 1.281 | 0.96 | 1.041 | 1.574 | 1.444 | 0.757 | 0.193 |
| P05154 | Plasma serine protease inhibitor | SERPINA5 | 71 | 14 | 1.049 | 1.192 | 0.96 | 0.955 | 0.982 | 1.121 | 1.047 | 0.608 |
| P05160 | Coagulation factor XIII B chain | F13B | 7 | 6 | 0.997 | 1.09 | 0.903 | 1.12 | 1.182 | 1.087 | 0.882 | 0.117 |
| P05164 | Myeloperoxidase | MPO | 46 | 20 | 0.945 | 1.017 | 1.005 | 1.019 | 0.939 | 1.023 | 0.996 | 0.907 |
| P05543 | Thyroxine-binding globulin | SERPINA7 | 3 | 1 | 0.882 | 0.896 | 0.82 | 0.965 | 0.949 | 0.999 | 0.892 | 0.026 |
| P05546 | Heparin cofactor 2 | SERPIND1 | 140 | 15 | 0.8 | 0.958 | 0.852 | 0.997 | 1.148 | 1.113 | 0.801 | 0.03 |
| P05981 | Serine protease hepsin | HPN | 1 | 1 | 0.9 | 0.863 | 0.627 | 0.667 | 0.844 | 0.677 | 1.093 | 0.554 |
| P06312 | Immunoglobulin kappa variable 4-1 | IGKV4-1 | 13 | 1 | 0.857 | 1.009 | 0.928 | 0.716 | 0.864 | 0.634 | 1.261 | 0.085 |
| P06396 | Gelsolin | GSN | 75 | 18 | 0.693 | 0.981 | 0.699 | 1.117 | 1.186 | 1.073 | 0.703 | 0.06 |
| P06727 | Apolipoprotein A-IV | APOA4 | 529 | 42 | 0.909 | 0.943 | 1.009 | 0.927 | 1.02 | 1.01 | 0.968 | 0.487 |
| P07093 | Glia-derived nexin | SERPINE2 | 3 | 2 | 0.881 | 0.928 | 0.901 | 1.061 | 1.131 | 1.031 | 0.841 | 0.016 |
| P07195 | L-lactate dehydrogenase B chain | LDHB | 9 | 4 | 0.924 | 1.049 | 0.75 | 0.852 | 1.186 | 0.904 | 0.926 | 0.619 |
| P07203 | Glutathione peroxidase 1 | GPX1 | 1 | 1 | 1.18 | 1.035 | 0.853 | 1.074 | 0.996 | 1.244 | 0.926 | 0.531 |
| P07357 | Complement component C8 alpha chain | C8A | 16 | 7 | 0.928 | 1.017 | 0.924 | 0.926 | 0.984 | 1.044 | 0.971 | 0.568 |
| P07359 | Platelet glycoprotein Ib alpha chain | GP1BA | 11 | 3 | 0.947 | 0.852 | 0.838 | 1.085 | 1.035 | 1.025 | 0.838 | 0.021 |
| P07360 | Complement component C8 gamma chain | C8G | 12 | 4 | 0.791 | 0.927 | 0.772 | 0.915 | 0.981 | 1.065 | 0.841 | 0.075 |
| P07437 | Tubulin beta chain | TUBB | 13 | 5 | 1.432 | 1.072 | 1.936 | 0.917 | 0.863 | 0.832 | 1.7 | 0.134 |
| P07737 | Profilin-1 | PFN1 | 1 | 1 | 0.964 | 1.224 | 1.036 | 1.029 | 0.918 | 1.075 | 1.067 | 0.506 |
| P07741 | Adenine phosphoribosyltransferase | APRT | 1 | 1 | 0.927 | 0.922 | 0.843 | 0.936 | 0.933 | 1.082 | 0.912 | 0.219 |
| P07996 | Thrombospondin-1 | THBS1 | 259 | 3 | 0.944 | 0.966 | 1.101 | 1.02 | 0.97 | 0.995 | 1.009 | 0.874 |
| P08246 | Neutrophil elastase | ELANE | 1 | 1 | 1.225 | 1.091 | 1.185 | 1.024 | 0.925 | 0.864 | 1.245 | 0.021 |
| P08294 | Extracellular superoxide dismutase [Cu-Zn] | SOD3 | 11 | 5 | 0.91 | 0.837 | 1.03 | 0.994 | 0.909 | 1.028 | 0.947 | 0.49 |
| P08311 | Cathepsin G | CTSG | 15 | 5 | 1.112 | 1.122 | 1.143 | 1.005 | 0.891 | 0.987 | 1.172 | 0.036 |
| P08493 | Matrix Gla protein | MGP | 3 | 1 | 1.086 | 1.145 | 1.067 | 0.779 | 0.932 | 0.95 | 1.239 | 0.043 |
| P08514 | Integrin alpha-IIb | ITGA2B | 1 | 1 | 0.846 | 1.811 | 1.026 | 1.201 | 1.08 | 1.023 | 1.114 | 0.714 |
| P08567 | Pleckstrin | PLEK | 2 | 1 | 0.918 | 0.997 | 0.841 | 1.207 | 1.05 | 1.152 | 0.809 | 0.028 |
| P08670 | Vimentin | VIM | 7 | 3 | 1.104 | 1.042 | 0.97 | 1.078 | 0.977 | 1.3 | 0.929 | 0.501 |
| P08697 | Alpha-2-antiplasmin | SERPINF2 | 185 | 14 | 1.053 | 1.077 | 0.946 | 1.064 | 0.988 | 1.055 | 0.99 | 0.831 |
| P08709 | Coagulation factor VII | F7 | 7 | 2 | 1.058 | 0.977 | 1.095 | 1.048 | 1.012 | 1.138 | 0.979 | 0.68 |
| P08779 | Keratin, type I cytoskeletal 16 | KRT16 | 142 | 16 | 0.402 | 0.465 | 0.732 | 0.855 | 0.819 | 2.21 | 0.412 | 0.235 |
| P08833 | Insulin-like growth factor-binding protein 1 | IGFBP1 | 8 | 4 | 0.826 | 1.029 | 0.685 | 1.225 | 1.436 | 1.092 | 0.677 | 0.046 |
| P09382 | Galectin-1 | LGALS1 | 7 | 5 | 0.979 | 1.008 | 0.913 | 1.004 | 0.959 | 1.007 | 0.976 | 0.518 |
| P0C0L4 | Complement C4-A | C4A | 1980 | 3 | 0.902 | 0.888 | 1.043 | 0.981 | 1.166 | 1.056 | 0.885 | 0.167 |
| P0DJI8 | Serum amyloid A-1 protein | SAA1 | 13 | 2 | 0.877 | 1.325 | 1.056 | 0.835 | 0.765 | 0.771 | 1.375 | 0.147 |
| P0DJI9 | Serum amyloid A-2 protein | SAA2 | 7 | 2 | 1.98 | 1.367 | 1.388 | 0.49 | 0.421 | 0.505 | 3.342 | 0.03 |
| P0DOX2 | Immunoglobulin alpha-2 heavy chain |  | 104 | 4 | 0.997 | 0.949 | 0.989 | 0.972 | 0.998 | 1.029 | 0.979 | 0.39 |
| P0DOX3 | Immunoglobulin delta heavy chain |  | 6 | 4 | 0.935 | 0.855 | 0.884 | 0.897 | 0.988 | 1.016 | 0.922 | 0.165 |
| P0DOX5 | Immunoglobulin gamma-1 heavy chain |  | 511 | 1 | 0.938 | 1.168 | 0.982 | 0.914 | 0.86 | 0.849 | 1.177 | 0.15 |
| P0DOX6 | Immunoglobulin mu heavy chain |  | 1026 | 1 | 0.969 | 0.923 | 2.015 | 0.941 | 0.824 | 1.138 | 1.346 | 0.45 |
| P0DOX7 | Immunoglobulin kappa light chain |  | 97 | 1 | 1.099 | 1.08 | 1.1 | 0.984 | 1.086 | 1.295 | 0.975 | 0.785 |
| P0DOX8 | Immunoglobulin lambda-1 light chain |  | 111 | 1 | 1.072 | 1.377 | 1.173 | 0.889 | 0.925 | 0.958 | 1.306 | 0.081 |
| P10645 | Chromogranin-A | CHGA | 11 | 5 | 1.057 | 1.048 | 1.466 | 0.763 | 0.834 | 0.756 | 1.517 | 0.094 |
| P10909 | Clusterin | CLU | 849 | 18 | 0.998 | 0.957 | 0.955 | 0.962 | 0.943 | 0.99 | 1.005 | 0.815 |
| P11047 | Laminin subunit gamma-1 | LAMC1 | 1 | 1 | 0.896 | 0.868 | 0.908 | 1.249 | 1.237 | 0.963 | 0.775 | 0.107 |
| P11226 | Mannose-binding protein C | MBL2 | 1 | 1 | 0.873 | 0.955 | 1.176 | 0.879 | 0.826 | 0.897 | 1.154 | 0.275 |
| P11678 | Eosinophil peroxidase | EPX | 11 | 6 | 1.071 | 1.253 | 0.842 | 1.072 | 1.16 | 1.107 | 0.948 | 0.68 |
| P12109 | Collagen alpha-1(VI) chain | COL6A1 | 3 | 3 | 0.803 | 1.064 | 0.737 | 0.916 | 0.844 | 0.899 | 0.98 | 0.875 |
| P12724 | Eosinophil cationic protein | RNASE3 | 22 | 5 | 1.138 | 1.427 | 1.185 | 0.969 | 0.928 | 1.007 | 1.292 | 0.079 |
| P12838 | Neutrophil defensin 4 | DEFA4 | 1 | 1 | 1.073 | 1.175 | 0.958 | 0.713 | 0.671 | 0.812 | 1.46 | 0.015 |
| P13010 | X-ray repair cross-complementing protein 5 | XRCC5 | 1 | 1 | 1.023 | 0.958 | 0.705 | 1.255 | 1.074 | 1.327 | 0.735 | 0.062 |
| P13489 | Ribonuclease inhibitor | RNH1 | 8 | 6 | 1.085 | 1.06 | 0.884 | 1.141 | 0.993 | 1.086 | 0.941 | 0.458 |
| P13521 | Secretogranin-2 | SCG2 | 1 | 1 | 1.087 | 1.101 | 1.062 | 0.918 | 0.859 | 0.972 | 1.182 | 0.026 |
| P13645 | Keratin, type I cytoskeletal 10 | KRT10 | 172 | 26 | 0.842 | 0.799 | 1.26 | 1.027 | 0.888 | 1.289 | 0.905 | 0.621 |
| P13647 | Keratin, type II cytoskeletal 5 | KRT5 | 137 | 16 | 0.665 | 0.827 | 1.021 | 1.001 | 0.961 | 1.337 | 0.762 | 0.173 |
| P13671 | Complement component C6 | C6 | 49 | 17 | 0.881 | 1.012 | 0.761 | 0.936 | 1.05 | 1.164 | 0.843 | 0.167 |
| P13727 | Bone marrow proteoglycan | PRG2 | 3 | 2 | 0.931 | 1.039 | 1.179 | 0.997 | 0.96 | 1.02 | 1.058 | 0.509 |
| P14543 | Nidogen-1 | NID1 | 47 | 25 | 0.884 | 0.915 | 0.987 | 1.042 | 1.05 | 1.089 | 0.876 | 0.033 |
| P14555 | Phospholipase A2, membrane associated | PLA2G2A | 4 | 3 | 1.534 | 2.426 | 1.954 | 0.229 | 0.228 | 0.225 | 8.679 | 0.021 |
| P14866 | Heterogeneous nuclear ribonucleoprotein L | HNRNPL | 1 | 1 | 0.805 | 0.941 | 1.372 | 1.008 | 1.05 | 1.025 | 1.012 | 0.951 |
| P15169 | Carboxypeptidase N catalytic chain | CPN1 | 9 | 3 | 0.867 | 0.968 | 0.867 | 1.007 | 0.977 | 1.036 | 0.895 | 0.069 |
| P15291 | Beta-1,4-galactosyltransferase 1 | B4GALT1 | 2 | 1 | 0.94 | 1.097 | 1.051 | 0.823 | 0.979 | 0.801 | 1.186 | 0.094 |
| P15814 | Immunoglobulin lambda-like polypeptide 1 | IGLL1 | 4 | 1 | 1.017 | 0.956 | 1.355 | 0.976 | 1.095 | 1.06 | 1.062 | 0.658 |
| P15907 | Beta-galactoside alpha-2,6-sialyltransferase 1 | ST6GAL1 | 1 | 1 | 1.004 | 1.077 | 0.968 | 0.979 | 0.766 | 0.842 | 1.178 | 0.116 |
| P15924 | Desmoplakin | DSP | 45 | 34 | 0.71 | 0.829 | 1.06 | 1.158 | 0.999 | 1.439 | 0.723 | 0.117 |
| P16070 | CD44 antigen | CD44 | 8 | 4 | 0.95 | 0.811 | 0.711 | 1.033 | 1.029 | 1.04 | 0.797 | 0.094 |
| P16157 | Ankyrin-1 | ANK1 | 1 | 1 | 0.994 | 1.132 | 0.863 | 1.196 | 1.157 | 1.264 | 0.826 | 0.099 |
| P16401 | Histone H1.5 | HIST1H1B | 3 | 2 | 1.754 | 1.506 | 1.22 | 0.98 | 0.861 | 0.89 | 1.641 | 0.057 |
| P16402 | Histone H1.3 | HIST1H1D | 5 | 3 | 1.274 | 1.468 | 1.126 | 1.048 | 0.962 | 1.074 | 1.254 | 0.106 |
| P17213 | Bactericidal permeability-increasing protein | BPI | 8 | 6 | 1.348 | 1.152 | 1.179 | 1.178 | 0.926 | 1.013 | 1.18 | 0.125 |
| P17900 | Ganglioside GM2 activator | GM2A | 2 | 2 | 1.117 | 1.145 | 0.994 | 1.044 | 0.863 | 1.135 | 1.07 | 0.493 |
| P17936 | Insulin-like growth factor-binding protein 3 | IGFBP3 | 18 | 8 | 1.084 | 0.993 | 0.859 | 1.035 | 1.139 | 1.122 | 0.891 | 0.201 |
| P18065 | Insulin-like growth factor-binding protein 2 | IGFBP2 | 1 | 1 | 0.996 | 1.003 | 1.046 | 0.823 | 0.785 | 0.841 | 1.244 | 0.001 |
| P18428 | Lipopolysaccharide-binding protein | LBP | 117 | 10 | 1.008 | 1.127 | 1.231 | 0.791 | 0.706 | 0.74 | 1.505 | 0.018 |
| P19320 | Vascular cell adhesion protein 1 | VCAM1 | 1 | 1 | 0.913 | 0.983 | 0.892 | 0.889 | 0.853 | 0.989 | 1.021 | 0.724 |
| P19652 | Alpha-1-acid glycoprotein 2 | ORM2 | 13 | 2 | 1.196 | 0.997 | 1.434 | 0.568 | 0.536 | 0.528 | 2.222 | 0.033 |
| P19883 | Follistatin | FST | 2 | 2 | 0.887 | 1.049 | 0.927 | 0.974 | 0.894 | 0.913 | 1.03 | 0.651 |
| P20160 | Azurocidin | AZU1 | 19 | 3 | 1.123 | 1.04 | 1.174 | 1.014 | 0.82 | 1.022 | 1.168 | 0.12 |
| P20618 | Proteasome subunit beta type-1 | PSMB1 | 1 | 1 | 0.878 | 1.038 | 0.862 | 1.023 | 0.873 | 0.97 | 0.969 | 0.701 |
| P20851 | C4b-binding protein beta chain | C4BPB | 67 | 9 | 1.061 | 0.946 | 1.098 | 0.846 | 0.891 | 0.859 | 1.196 | 0.056 |
| P21333 | Filamin-A | FLNA | 11 | 9 | 0.973 | 1.375 | 1.083 | 0.908 | 0.697 | 0.694 | 1.493 | 0.067 |
| P22314 | Ubiquitin-like modifier-activating enzyme 1 | UBA1 | 1 | 1 | 0.864 | 0.927 | 1.018 | 1.066 | 0.968 | 1.241 | 0.857 | 0.184 |
| P22352 | Glutathione peroxidase 3 | GPX3 | 78 | 8 | 0.954 | 0.898 | 1.157 | 0.863 | 0.888 | 0.855 | 1.155 | 0.229 |
| P22792 | Carboxypeptidase N subunit 2 | CPN2 | 35 | 11 | 0.9 | 0.884 | 0.846 | 1.049 | 1.042 | 1.036 | 0.841 | 0.007 |
| P23142 | Fibulin-1 | FBLN1 | 353 | 8 | 1.006 | 1.026 | 1.046 | 1.089 | 1.002 | 0.957 | 1.01 | 0.824 |
| P23471 | Receptor-type tyrosine-protein phosphatase zeta | PTPRZ1 | 1 | 1 | 1.056 | 0.982 | 0.895 | 1.386 | 1.339 | 1.256 | 0.737 | 0.005 |
| P24158 | Myeloblastin | PRTN3 | 2 | 1 | 1.027 | 0.979 | 1.064 | 0.713 | 0.829 | 0.912 | 1.251 | 0.055 |
| P24387 | Corticotropin-releasing factor-binding protein | CRHBP | 8 | 5 | 0.901 | 0.966 | 0.864 | 0.984 | 0.989 | 0.981 | 0.925 | 0.129 |
| P25788 | Proteasome subunit alpha type-3 | PSMA3 | 1 | 1 | 0.883 | 0.838 | 0.893 | 0.857 | 0.835 | 0.869 | 1.021 | 0.429 |
| P26022 | Pentraxin-related protein PTX3 | PTX3 | 2 | 2 | 0.814 | 0.874 | 0.825 | 0.995 | 0.931 | 0.943 | 0.876 | 0.012 |
| P26447 | Protein S100-A4 | S100A4 | 1 | 1 | 1.204 | 1.125 | 0.716 | 1.109 | 0.924 | 1.071 | 0.981 | 0.911 |
| P27169 | Serum paraoxonase/arylesterase 1 | PON1 | 104 | 6 | 0.877 | 0.941 | 0.836 | 0.998 | 1.115 | 1.041 | 0.842 | 0.023 |
| P27824 | Calnexin | CANX | 1 | 1 | 0.876 | 0.876 | 0.913 | 0.903 | 0.92 | 0.94 | 0.965 | 0.114 |
| P27918 | Properdin | CFP | 215 | 16 | 1.152 | 1.087 | 0.845 | 1.093 | 1.171 | 1.146 | 0.904 | 0.365 |
| P28072 | Proteasome subunit beta type-6 | PSMB6 | 2 | 2 | 0.866 | 1.026 | 1.551 | 1.005 | 1.006 | 1.033 | 1.13 | 0.588 |
| P28676 | Grancalcin | GCA | 3 | 2 | 1.042 | 0.942 | 1.368 | 0.993 | 0.971 | 1.009 | 1.128 | 0.43 |
| P28799 | Granulins | GRN | 3 | 2 | 1.156 | 1.059 | 1.017 | 0.971 | 0.851 | 0.962 | 1.161 | 0.057 |
| P29508 | Serpin B3 | SERPINB3 | 1 | 1 | 0.952 | 0.943 | 0.892 | 1.071 | 1.014 | 1.312 | 0.82 | 0.151 |
| P30041 | Peroxiredoxin-6 | PRDX6 | 7 | 5 | 1.041 | 1.053 | 0.871 | 1.056 | 1.044 | 0.881 | 0.995 | 0.951 |
| P30043 | Flavin reductase (NADPH) | BLVRB | 2 | 2 | 1.146 | 0.995 | 1.038 | 0.624 | 0.625 | 0.998 | 1.415 | 0.118 |
| P30044 | Peroxiredoxin-5, mitochondrial | PRDX5 | 2 | 2 | 0.987 | 0.874 | 0.889 | 1.223 | 1.698 | 1.092 | 0.685 | 0.145 |
| P30530 | Tyrosine-protein kinase receptor UFO | AXL | 2 | 2 | 0.868 | 1.092 | 0.895 | 1.053 | 0.991 | 1.108 | 0.906 | 0.299 |
| P31151 | Protein S100-A7 | S100A7 | 4 | 2 | 1.237 | 1.034 | 0.907 | 0.889 | 0.942 | 0.891 | 1.168 | 0.252 |
| P32119 | Peroxiredoxin-2 | PRDX2 | 26 | 8 | 0.969 | 1.145 | 1.049 | 1.182 | 1.098 | 1.336 | 0.874 | 0.161 |
| P33908 | Mannosyl-oligosaccharide 1,2-alpha-mannosidase IA | MAN1A1 | 31 | 10 | 0.983 | 1.095 | 0.95 | 1.043 | 0.881 | 1.148 | 0.986 | 0.88 |
| P35443 | Thrombospondin-4 | THBS4 | 13 | 5 | 1.06 | 1.055 | 0.858 | 1.184 | 1.077 | 0.975 | 0.919 | 0.386 |
| P35527 | Keratin, type I cytoskeletal 9 | KRT9 | 189 | 29 | 0.722 | 0.916 | 1.161 | 0.906 | 0.95 | 1.002 | 0.98 | 0.893 |
| P35555 | Fibrillin-1 | FBN1 | 42 | 28 | 1.088 | 1.008 | 1.039 | 0.903 | 0.907 | 0.884 | 1.164 | 0.017 |
| P35625 | Metalloproteinase inhibitor 3 | TIMP3 | 15 | 8 | 1.07 | 1.059 | 1.447 | 0.901 | 0.91 | 0.873 | 1.332 | 0.144 |
| P35908 | Keratin, type II cytoskeletal 2 epidermal | KRT2 | 145 | 22 | 0.896 | 0.853 | 1.272 | 0.868 | 0.729 | 1.39 | 1.011 | 0.966 |
| P36955 | Pigment epithelium-derived factor | SERPINF1 | 1 | 1 | 0.81 | 0.91 | 1.421 | 1.037 | 1.003 | 0.962 | 1.046 | 0.831 |
| P36959 | GMP reductase 1 | GMPR | 2 | 2 | 1.049 | 0.932 | 1.038 | 1.152 | 1.063 | 1.216 | 0.88 | 0.078 |
| P36980 | Complement factor H-related protein 2 | CFHR2 | 227 | 5 | 0.858 | 0.89 | 0.815 | 1.222 | 1.305 | 1.089 | 0.709 | 0.021 |
| P37802 | Transgelin-2 | TAGLN2 | 2 | 1 | 1.032 | 1.083 | 1.265 | 1.133 | 1.018 | 1.151 | 1.024 | 0.769 |
| P39060 | Collagen alpha-1(XVIII) chain | COL18A1 | 9 | 5 | 0.973 | 0.908 | 1.019 | 0.97 | 0.985 | 0.973 | 0.99 | 0.794 |
| P40197 | Platelet glycoprotein V | GP5 | 1 | 1 | 1.117 | 1.07 | 0.791 | 1.159 | 1.174 | 1.266 | 0.827 | 0.17 |
| P43251 | Biotinidase | BTD | 16 | 6 | 0.959 | 0.958 | 0.917 | 0.88 | 0.941 | 0.922 | 1.033 | 0.257 |
| P43652 | Afamin | AFM | 43 | 22 | 1.075 | 0.987 | 0.953 | 0.993 | 1.025 | 1.001 | 0.999 | 0.978 |
| P48061 | Stromal cell-derived factor 1 | CXCL12 | 11 | 4 | 1.011 | 1.059 | 0.873 | 0.937 | 0.999 | 1.014 | 0.998 | 0.975 |
| P48735 | Isocitrate dehydrogenase [NADP], mitochondrial | IDH2 | 11 | 1 | 0.772 | 0.851 | 0.868 | 0.909 | 0.827 | 0.993 | 0.913 | 0.245 |
| P48740 | Mannan-binding lectin serine protease 1 | MASP1 | 81 | 19 | 0.961 | 1.024 | 0.988 | 0.983 | 0.976 | 0.91 | 1.036 | 0.309 |
| P49641 | Alpha-mannosidase 2x | MAN2A2 | 1 | 1 | 0.847 | 0.955 | 1.125 | 1.385 | 1.364 | 1.561 | 0.679 | 0.012 |
| P49767 | Vascular endothelial growth factor C | VEGFC | 9 | 7 | 1.072 | 1.019 | 0.942 | 1.008 | 0.936 | 1.135 | 0.985 | 0.837 |
| P49773 | Histidine triad nucleotide-binding protein 1 | HINT1 | 2 | 2 | 1.38 | 1.048 | 1.14 | 1.157 | 0.851 | 1.008 | 1.183 | 0.239 |
| P49908 | Selenoprotein P | SELENOP | 35 | 8 | 1.001 | 0.894 | 0.963 | 1.011 | 0.929 | 1.011 | 0.969 | 0.497 |
| P50502 | Hsc70-interacting protein | ST13 | 3 | 2 | 1.095 | 0.875 | 0.783 | 1.113 | 1.1 | 1.018 | 0.852 | 0.222 |
| P51149 | Ras-related protein Rab-7a | RAB7A | 2 | 2 | 0.939 | 0.903 | 1.099 | 0.827 | 0.776 | 0.665 | 1.296 | 0.046 |
| P51884 | Lumican | LUM | 32 | 9 | 1.042 | 0.966 | 1.04 | 1.043 | 1.028 | 0.987 | 0.997 | 0.921 |
| P52209 | 6-phosphogluconate dehydrogenase, decarboxylating | PGD | 5 | 4 | 0.955 | 1.209 | 0.875 | 1.078 | 1.088 | 1.012 | 0.956 | 0.691 |
| P54920 | Alpha-soluble NSF attachment protein | NAPA | 2 | 2 | 1.213 | 1.125 | 1.092 | 1.103 | 1.209 | 1.145 | 0.992 | 0.857 |
| P55056 | Apolipoprotein C-IV | APOC4 | 19 | 3 | 1.05 | 1.135 | 1.024 | 0.844 | 0.885 | 0.803 | 1.268 | 0.007 |
| P55072 | Transitional endoplasmic reticulum ATPase | VCP | 10 | 9 | 1.188 | 1.028 | 0.957 | 1.216 | 1.1 | 1.236 | 0.893 | 0.204 |
| P55774 | C-C motif chemokine 18 | CCL18 | 5 | 2 | 1.589 | 1.376 | 1.23 | 0.682 | 0.729 | 0.909 | 1.808 | 0.011 |
| P56202 | Cathepsin W | CTSW | 2 | 2 | 0.793 | 0.857 | 0.694 | 0.941 | 1.079 | 1.063 | 0.76 | 0.019 |
| P58166 | Inhibin beta E chain | INHBE | 5 | 3 | 0.925 | 0.954 | 0.942 | 1 | 0.9 | 1.003 | 0.972 | 0.507 |
| P58215 | Lysyl oxidase homolog 3 | LOXL3 | 1 | 1 | 0.835 | 0.979 | 0.825 | 1.034 | 1.082 | 1.006 | 0.846 | 0.066 |
| P58546 | Myotrophin | MTPN | 1 | 1 | 0.873 | 0.87 | 0.782 | 1.039 | 0.991 | 1.135 | 0.798 | 0.018 |
| P59665 | Neutrophil defensin 1 | DEFA1 | 49 | 4 | 1.208 | 1.267 | 1.199 | 1.099 | 0.747 | 1.012 | 1.285 | 0.119 |
| P60709 | Actin, cytoplasmic 1 | ACTB | 109 | 15 | 0.965 | 1.067 | 1.543 | 0.855 | 0.75 | 0.786 | 1.495 | 0.154 |
| P61006 | Ras-related protein Rab-8A | RAB8A | 4 | 3 | 1.011 | 1.057 | 1.147 | 0.83 | 0.882 | 0.822 | 1.269 | 0.016 |
| P61081 | NEDD8-conjugating enzyme Ubc12 | UBE2M | 1 | 1 | 1.084 | 1.036 | 1.302 | 0.852 | 0.895 | 0.836 | 1.324 | 0.07 |
| P61626 | Lysozyme C | LYZ | 51 | 8 | 1.094 | 0.954 | 1.051 | 0.804 | 0.872 | 0.85 | 1.227 | 0.027 |
| P68871 | Hemoglobin subunit beta | HBB | 67 | 3 | 1.814 | 2.078 | 0.702 | 1.8 | 0.692 | 1.185 | 1.25 | 0.596 |
| P78386 | Keratin, type II cuticular Hb5 | KRT85 | 6 | 2 | 0.654 | 0.614 | 0.68 | 0.408 | 0.414 | 0.47 | 1.509 | 0 |
| P78492 | Inter-alpha-trypsin inhibitor (Fragment) | ITIL | 76 | 1 | 1.01 | 0.687 | 0.798 | 0.48 | 0.642 | 0.538 | 1.502 | 0.08 |
| P80108 | Phosphatidylinositol-glycan-specific phospholipase D | GPLD1 | 48 | 16 | 0.801 | 0.829 | 0.842 | 1.021 | 1.579 | 1.063 | 0.675 | 0.157 |
| P81605 | Dermcidin | DCD | 12 | 2 | 1.001 | 0.987 | 1.109 | 0.877 | 0.793 | 1.129 | 1.107 | 0.435 |
| Q01469 | Fatty acid-binding protein 5 | FABP5 | 1 | 1 | 0.832 | 0.772 | 2.84 | 1.257 | 1.021 | 1.334 | 1.231 | 0.724 |
| Q01523 | Defensin-5 | DEFA5 | 2 | 1 | 0.999 | 1.219 | 0.956 | 1.108 | 0.877 | 0.961 | 1.078 | 0.511 |
| Q02413 | Desmoglein-1 | DSG1 | 8 | 7 | 0.738 | 0.736 | 0.977 | 1.045 | 0.962 | 1.31 | 0.739 | 0.099 |
| Q02985 | Complement factor H-related protein 3 | CFHR3 | 119 | 2 | 0.892 | 1.255 | 0.983 | 0.619 | 0.619 | 0.633 | 1.673 | 0.061 |
| Q03167 | Transforming growth factor beta receptor type 3 | TGFBR3 | 5 | 4 | 0.655 | 0.794 | 0.675 | 0.874 | 0.861 | 0.884 | 0.811 | 0.059 |
| Q03591 | Complement factor H-related protein 1 | CFHR1 | 583 | 3 | 1.126 | 0.971 | 0.995 | 1.069 | 1.046 | 1.153 | 0.946 | 0.376 |
| Q04695 | Keratin, type I cytoskeletal 17 | KRT17 | 85 | 5 | 0.537 | 0.598 | 0.617 | 0.793 | 0.942 | 3.481 | 0.336 | 0.317 |
| Q04756 | Hepatocyte growth factor activator | HGFAC | 22 | 11 | 0.842 | 0.692 | 0.796 | 1.071 | 1.356 | 1.051 | 0.67 | 0.043 |
| Q05315 | Galectin-10 | CLC | 1 | 1 | 1.34 | 1.144 | 0.876 | 1.1 | 1.014 | 1.063 | 1.057 | 0.697 |
| Q05639 | Elongation factor 1-alpha 2 | EEF1A2 | 5 | 4 | 0.967 | 0.892 | 0.932 | 0.975 | 1.176 | 1.05 | 0.872 | 0.132 |
| Q06033 | Inter-alpha-trypsin inhibitor heavy chain H3 | ITIH3 | 109 | 17 | 0.986 | 0.976 | 1.252 | 0.644 | 0.621 | 0.622 | 1.704 | 0.038 |
| Q06481 | Amyloid-like protein 2 | APLP2 | 8 | 4 | 0.936 | 0.952 | 1.033 | 1.087 | 0.991 | 0.943 | 0.967 | 0.561 |
| Q07507 | Dermatopontin | DPT | 6 | 3 | 1.309 | 1.301 | 1.316 | 1.01 | 0.877 | 0.92 | 1.399 | 0.01 |
| Q07954 | Prolow-density lipoprotein receptor-related protein 1 | LRP1 | 39 | 26 | 1.032 | 0.992 | 0.96 | 1.085 | 1.046 | 1.139 | 0.913 | 0.052 |
| Q08554 | Desmocollin-1 | DSC1 | 2 | 2 | 0.853 | 0.824 | 1.117 | 1.027 | 0.927 | 1.084 | 0.92 | 0.492 |
| Q08830 | Fibrinogen-like protein 1 | FGL1 | 2 | 1 | 1.032 | 1.18 | 1.104 | 0.438 | 0.39 | 0.398 | 2.706 | 0.002 |
| Q0IIN1 | Keratin 77 | KRT77 | 62 | 3 | 0.998 | 0.973 | 1.299 | 0.998 | 0.996 | 1.23 | 1.014 | 0.913 |
| Q0PNF2 | FEX1 |  | 3 | 3 | 1.133 | 0.983 | 1.066 | 1.101 | 0.939 | 1.081 | 1.02 | 0.773 |
| Q0VD83 | Apolipoprotein B receptor | APOBR | 3 | 3 | 0.881 | 0.992 | 1.049 | 0.842 | 0.832 | 0.888 | 1.14 | 0.125 |
| Q0ZCH6 | Immunglobulin heavy chain variable region (Fragment) |  | 17 | 4 | 1.06 | 0.89 | 0.898 | 1.329 | 1.011 | 0.9 | 0.879 | 0.427 |
| Q0ZCH9 | Immunglobulin heavy chain variable region (Fragment) |  | 71 | 1 | 1.14 | 1.172 | 1.054 | 1.079 | 1.042 | 1.049 | 1.062 | 0.198 |
| Q0ZCI2 | Immunglobulin heavy chain variable region (Fragment) |  | 10 | 2 | 0.93 | 1.19 | 0.58 | 1.014 | 1.325 | 0.925 | 0.827 | 0.436 |
| Q0ZCJ1 | Immunglobulin heavy chain variable region (Fragment) |  | 12 | 1 | 1.065 | 0.98 | 0.869 | 0.948 | 0.799 | 0.892 | 1.104 | 0.274 |
| Q0ZGT2 | Nexilin | NEXN | 4 | 3 | 1.002 | 0.879 | 2.212 | 0.613 | 0.581 | 0.488 | 2.432 | 0.199 |
| Q12841 | Follistatin-related protein 1 | FSTL1 | 3 | 2 | 0.921 | 0.99 | 0.784 | 1.089 | 1.175 | 1.112 | 0.798 | 0.048 |
| Q13103 | Secreted phosphoprotein 24 | SPP2 | 22 | 5 | 0.832 | 0.982 | 1.51 | 0.986 | 1.021 | 0.983 | 1.112 | 0.643 |
| Q13201 | Multimerin-1 | MMRN1 | 39 | 21 | 1.028 | 1.15 | 1.12 | 0.908 | 0.894 | 0.929 | 1.207 | 0.029 |
| Q13228 | Methanethiol oxidase | SELENBP1 | 4 | 4 | 1.046 | 1.042 | 0.858 | 1.187 | 1.145 | 1.129 | 0.851 | 0.1 |
| Q13444 | Disintegrin and metalloproteinase domain-containing protein 15 | ADAM15 | 1 | 1 | 1.03 | 1.069 | 1.088 | 0.922 | 0.691 | 1.03 | 1.205 | 0.209 |
| Q13790 | Apolipoprotein F | APOF | 14 | 4 | 0.897 | 0.908 | 0.868 | 1.083 | 1.14 | 1.321 | 0.754 | 0.053 |
| Q13835 | Plakophilin-1 | PKP1 | 1 | 1 | 0.806 | 0.741 | 1.026 | 0.937 | 0.855 | 1.283 | 0.837 | 0.355 |
| Q14112 | Nidogen-2 | NID2 | 17 | 9 | 0.97 | 0.909 | 0.869 | 1.087 | 1.067 | 1.175 | 0.825 | 0.012 |
| Q14247 | Src substrate cortactin | CTTN | 1 | 1 | 1.203 | 1.019 | 1.68 | 0.884 | 0.769 | 0.688 | 1.667 | 0.109 |
| Q14314 | Fibroleukin | FGL2 | 2 | 1 | 1.267 | 1.228 | 1.14 | 0.986 | 0.895 | 0.977 | 1.272 | 0.007 |
| Q14393 | Growth arrest-specific protein 6 | GAS6 | 1 | 1 | 0.97 | 0.973 | 0.957 | 1.058 | 1.12 | 0.996 | 0.914 | 0.121 |
| Q14515 | SPARC-like protein 1 | SPARCL1 | 25 | 10 | 0.961 | 0.884 | 0.82 | 1.108 | 1.083 | 1.021 | 0.83 | 0.026 |
| Q14520 | Hyaluronan-binding protein 2 | HABP2 | 1523 | 28 | 1.128 | 1.136 | 1.033 | 1.061 | 0.997 | 0.968 | 1.09 | 0.107 |
| Q14766 | Latent-transforming growth factor beta-binding protein 1 | LTBP1 | 109 | 4 | 0.962 | 1.029 | 0.918 | 1.013 | 0.937 | 0.946 | 1.005 | 0.917 |
| Q14767 | Latent-transforming growth factor beta-binding protein 2 | LTBP2 | 1 | 1 | 1.022 | 0.984 | 0.93 | 1.083 | 1.099 | 1.111 | 0.892 | 0.038 |
| Q15063 | Periostin | POSTN | 1 | 1 | 0.771 | 0.861 | 0.997 | 1.069 | 0.891 | 1.158 | 0.843 | 0.189 |
| Q15102 | Platelet-activating factor acetylhydrolase IB subunit gamma | PAFAH1B3 | 2 | 1 | 1.189 | 1.104 | 1.142 | 1.111 | 1.257 | 1.357 | 0.922 | 0.307 |
| Q15166 | Serum paraoxonase/lactonase 3 | PON3 | 60 | 8 | 0.888 | 0.867 | 0.746 | 1.041 | 1.276 | 1.146 | 0.722 | 0.023 |
| Q15323 | Keratin, type I cuticular Ha1 | KRT31 | 11 | 1 | 0.71 | 0.998 | 0.837 | 0.609 | 0.593 | 0.56 | 1.444 | 0.084 |
| Q15389 | Angiopoietin-1 | ANGPT1 | 6 | 5 | 0.875 | 1.144 | 1.021 | 1.098 | 1.121 | 1.495 | 0.818 | 0.224 |
| Q15430 | Coagulation factor V (Fragment) | F5 | 13 | 1 | 0.956 | 1.005 | 0.963 | 0.79 | 1.355 | 1.03 | 0.921 | 0.662 |
| Q15485 | Ficolin-2 | FCN2 | 27 | 7 | 0.99 | 1.194 | 1.264 | 0.866 | 0.841 | 0.944 | 1.301 | 0.069 |
| Q15555 | Microtubule-associated protein RP/EB family member 2 | MAPRE2 | 1 | 1 | 1.1 | 0.996 | 1.121 | 0.958 | 0.871 | 0.94 | 1.162 | 0.039 |
| Q15582 | Transforming growth factor-beta-induced protein ig-h3 | TGFBI | 11 | 7 | 0.911 | 0.931 | 0.842 | 1.001 | 0.934 | 0.969 | 0.924 | 0.099 |
| Q15691 | Microtubule-associated protein RP/EB family member 1 | MAPRE1 | 2 | 2 | 0.992 | 0.994 | 1.316 | 0.999 | 0.964 | 0.885 | 1.159 | 0.294 |
| Q15746 | Myosin light chain kinase, smooth muscle | MYLK | 1 | 1 | 1.216 | 1.031 | 1.513 | 0.739 | 0.78 | 0.632 | 1.748 | 0.051 |
| Q16270 | Insulin-like growth factor-binding protein 7 | IGFBP7 | 1 | 1 | 0.798 | 0.827 | 0.587 | 1.05 | 0.86 | 1.367 | 0.675 | 0.123 |
| Q16769 | Glutaminyl-peptide cyclotransferase | QPCT | 5 | 2 | 0.947 | 0.957 | 1.092 | 1.052 | 1.382 | 0.993 | 0.875 | 0.361 |
| Q1HP67 | Lipoprotein, Lp(A) | LPA | 45 | 22 | 0.954 | 1.168 | 1.145 | 1.081 | 0.908 | 0.746 | 1.194 | 0.216 |
| Q32M96 | Platelet-derived growth factor alpha polypeptide | PDGFA | 8 | 3 | 0.972 | 1.098 | 0.966 | 0.978 | 0.92 | 1.015 | 1.042 | 0.478 |
| Q3ZCW2 | Galectin-related protein | LGALSL | 1 | 1 | 0.902 | 1.022 | 0.978 | 0.843 | 0.84 | 0.969 | 1.094 | 0.209 |
| Q4LE33 | TNC variant protein (Fragment) | TNC | 7 | 7 | 1.078 | 0.945 | 1.198 | 0.997 | 0.962 | 0.919 | 1.12 | 0.252 |
| Q4ZG40 | Macrophage receptor with collagenous structure | MARCO | 1 | 1 | 0.89 | 0.993 | 0.819 | 0.796 | 0.869 | 0.807 | 1.093 | 0.267 |
| Q53H76 | Phospholipase A1 member A | PLA1A | 3 | 3 | 0.784 | 1.007 | 0.824 | 0.971 | 0.918 | 0.946 | 0.923 | 0.399 |
| Q53RD9 | Fibulin-7 | FBLN7 | 2 | 2 | 0.869 | 0.861 | 0.847 | 1.059 | 0.927 | 1.07 | 0.843 | 0.071 |
| Q53XB4 | Full-length cDNA clone CS0DF032YM23 of Fetal brain of Homo sapiens (human) | RAB1 | 31 | 6 | 1.172 | 1.03 | 1.161 | 0.921 | 0.928 | 0.93 | 1.21 | 0.05 |
| Q569I7 | Uncharacterized protein |  | 192 | 1 | 0.757 | 1.068 | 0.944 | 1.083 | 0.944 | 1.027 | 0.907 | 0.413 |
| Q59EG8 | Proteasome 26S non-ATPase subunit 2 variant (Fragment) |  | 2 | 2 | 0.977 | 0.915 | 0.758 | 1.101 | 1.053 | 1.067 | 0.823 | 0.093 |
| Q59EH3 | Acid phosphatase 1 isoform c variant (Fragment) |  | 1 | 1 | 1.206 | 0.988 | 0.969 | 0.97 | 0.972 | 0.983 | 1.082 | 0.406 |
| Q59EN5 | Prosaposin variant (Fragment) |  | 10 | 7 | 1.083 | 0.938 | 1.03 | 0.812 | 0.776 | 0.739 | 1.311 | 0.015 |
| Q59EP2 | Angiotensinogen variant (Fragment) |  | 22 | 7 | 0.577 | 0.85 | 0.648 | 0.76 | 1.874 | 0.825 | 0.6 | 0.328 |
| Q59ER5 | WD repeat-containing protein 1 isoform 1 variant (Fragment) |  | 9 | 8 | 0.923 | 1.05 | 0.808 | 1.047 | 0.908 | 1.037 | 0.93 | 0.453 |
| Q59FP5 | Spectrin, beta, erythrocytic (Includes spherocytosis, clinical type I) variant (Fragment) |  | 3 | 3 | 0.948 | 1.15 | 0.865 | 1.126 | 1.053 | 1.154 | 0.889 | 0.279 |
| Q59G48 | C1q and tumor necrosis factor related protein 3 variant (Fragment) |  | 1 | 1 | 0.873 | 0.771 | 0.809 | 1.051 | 1.071 | 1.421 | 0.692 | 0.087 |
| Q59G70 | Mannosyl (Alpha-1,3-)-glycoprotein beta-1,2-N-acetylglucosaminyltransferase variant (Fragment) |  | 1 | 1 | 0.691 | 0.85 | 0.823 | 1.002 | 0.917 | 1.064 | 0.792 | 0.035 |
| Q59H08 | Hexokinase 3 variant (Fragment) |  | 3 | 3 | 0.864 | 1.018 | 0.944 | 1.119 | 0.981 | 1.057 | 0.895 | 0.139 |
| Q59H77 | T-complex protein 1 subunit gamma (Fragment) |  | 2 | 2 | 1.144 | 1.083 | 1.015 | 0.926 | 0.845 | 0.923 | 1.203 | 0.02 |
| Q5CZ94 | Uncharacterized protein DKFZp781M0386 | DKFZp781M0386 | 90 | 1 | 1.114 | 0.915 | 1.492 | 1.237 | 0.809 | 0.726 | 1.27 | 0.342 |
| Q5EC54 | Heterogeneous nuclear ribonucleoprotein K transcript variant | HNRPK | 1 | 1 | 1.168 | 1.038 | 0.885 | 1.014 | 0.897 | 1.003 | 1.061 | 0.56 |
| Q5FWF9 | IGL@ protein | IGL@ | 153 | 5 | 1.039 | 1.017 | 1.05 | 0.914 | 0.879 | 0.937 | 1.138 | 0.006 |
| Q5HYE3 | Uncharacterized protein DKFZp686H1812 | DKFZp686H1812 | 1 | 1 | 0.872 | 0.958 | 0.757 | 1.199 | 1.158 | 1.228 | 0.722 | 0.02 |
| Q5IWS5 | Intelectin 1 | ITLN1 | 28 | 8 | 1.135 | 1.236 | 1.144 | 0.953 | 0.928 | 0.848 | 1.288 | 0.004 |
| Q5JVE7 | Coagulation factor X, isoform CRA_a | F10 | 83 | 14 | 1.008 | 0.93 | 0.982 | 0.971 | 0.99 | 0.992 | 0.989 | 0.686 |
| Q5NV91 | V2-19 protein (Fragment) | V2-19 | 9 | 3 | 0.689 | 1.022 | 0.661 | 0.509 | 0.597 | 0.588 | 1.4 | 0.186 |
| Q5T749 | Keratinocyte proline-rich protein | KPRP | 1 | 1 | 0.44 | 1.161 | 0.689 | 0.9 | 0.9 | 1.076 | 0.796 | 0.456 |
| Q5T9B7 | Adenylate kinase isoenzyme 1 | AK1 | 3 | 2 | 1.163 | 0.979 | 0.898 | 1.009 | 0.885 | 1.112 | 1.011 | 0.916 |
| Q5TDH0 | Protein DDI1 homolog 2 | DDI2 | 5 | 4 | 1.269 | 1.043 | 1.147 | 1.186 | 1.065 | 1.373 | 0.955 | 0.651 |
| Q5U0B9 | Stem cell growth factor lymphocyte secreted C-type lectin |  | 6 | 5 | 0.831 | 0.852 | 0.895 | 0.967 | 0.859 | 1.052 | 0.896 | 0.208 |
| Q65ZC9 | Single-chain Fv (Fragment) | scFv | 29 | 1 | 0.991 | 1.025 | 1.143 | 0.899 | 1.126 | 0.935 | 1.067 | 0.481 |
| Q6GMX6 | IGH@ protein | IGH@ | 535 | 2 | 1.025 | 0.936 | 1.016 | 0.838 | 0.849 | 0.796 | 1.199 | 0.013 |
| Q6IN99 | IGL@ protein | IGL@ | 156 | 1 | 1.144 | 1.073 | 0.966 | 0.789 | 0.898 | 0.752 | 1.305 | 0.023 |
| Q6LBM9 | FHR-1 complement Factor H-related protein 1 | H | 109 | 1 | 0.931 | 0.722 | 0.724 | 1.022 | 1.004 | 1.053 | 0.772 | 0.073 |
| Q6MZL2 | Uncharacterized protein DKFZp686M0562 (Fragment) | DKFZp686M0562 | 22 | 6 | 0.965 | 1.053 | 0.814 | 1.053 | 1.087 | 1 | 0.902 | 0.275 |
| Q6MZQ6 | Uncharacterized protein DKFZp686G11190 | DKFZp686G11190 | 523 | 2 | 1.2 | 1.014 | 1.331 | 1.002 | 1.102 | 0.984 | 1.148 | 0.235 |
| Q6N091 | Uncharacterized protein DKFZp686C02220 (Fragment) | DKFZp686C02220 | 58 | 1 | 1.189 | 0.942 | 1.266 | 0.941 | 0.997 | 1.141 | 1.103 | 0.415 |
| Q6N093 | Uncharacterized protein DKFZp686I04196 (Fragment) | DKFZp686I04196 | 269 | 4 | 1.222 | 1.001 | 0.889 | 1.047 | 0.921 | 0.903 | 1.084 | 0.512 |
| Q6N094 | Uncharacterized protein DKFZp686O01196 | DKFZp686O01196 | 512 | 1 | 0.986 | 1.059 | 0.784 | 0.741 | 0.719 | 0.605 | 1.37 | 0.071 |
| Q6NS95 | IGL@ protein | IGL@ | 112 | 1 | 0.915 | 0.962 | 1.076 | 1.019 | 0.91 | 0.972 | 1.018 | 0.781 |
| Q6NUJ1 | Proactivator polypeptide-like 1 | PSAPL1 | 2 | 1 | 0.8 | 0.922 | 1.811 | 1.209 | 1.39 | 1.134 | 0.947 | 0.856 |
| Q6P089 | IGH@ protein | IGH@ | 102 | 1 | 1.312 | 1.315 | 1.46 | 0.83 | 0.723 | 0.782 | 1.75 | 0.001 |
| Q6P528 | Asporin | ASPN | 4 | 2 | 1.049 | 1.077 | 1.391 | 1.236 | 1.316 | 0.94 | 1.007 | 0.959 |
| Q6P988 | Palmitoleoyl-protein carboxylesterase NOTUM | NOTUM | 1 | 1 | 1.013 | 0.99 | 1.466 | 0.849 | 1.03 | 0.943 | 1.229 | 0.297 |
| Q6PIQ7 | IGL@ protein | IGL@ | 184 | 1 | 1.124 | 1.136 | 1.471 | 0.569 | 0.536 | 0.639 | 2.138 | 0.022 |
| Q6Q788 | Apolipoprotein A-V | APOA5 | 18 | 11 | 0.917 | 1.048 | 1.127 | 0.995 | 1.003 | 1.064 | 1.01 | 0.891 |
| Q6S4P3 | Ferritin | FTL | 1 | 1 | 0.907 | 0.98 | 1.103 | 1.087 | 0.889 | 1.078 | 0.979 | 0.814 |
| Q6UX71 | Plexin domain-containing protein 2 | PLXDC2 | 1 | 1 | 1.005 | 1.048 | 1.234 | 1.176 | 1.097 | 1.096 | 0.976 | 0.745 |
| Q6UXB8 | Peptidase inhibitor 16 | PI16 | 1 | 1 | 1.019 | 0.943 | 0.886 | 1.275 | 1.397 | 1.068 | 0.762 | 0.075 |
| Q6UXH0 | Angiopoietin-like protein 8 | ANGPTL8 | 2 | 2 | 0.935 | 0.851 | 1.194 | 0.823 | 0.908 | 0.76 | 1.197 | 0.25 |
| Q6UY14 | ADAMTS-like protein 4 | ADAMTSL4 | 17 | 12 | 1.057 | 0.997 | 1.055 | 0.907 | 0.919 | 0.921 | 1.132 | 0.021 |
| Q6ZMU0 | Delta-aminolevulinic acid dehydratase |  | 1 | 1 | 1.022 | 0.921 | 1.134 | 1.111 | 0.911 | 1.022 | 1.011 | 0.904 |
| Q6ZNX5 | cDNA FLJ26936 fis, clone RCT06808 |  | 3 | 2 | 0.665 | 0.981 | 0.622 | 0.952 | 0.811 | 0.832 | 0.874 | 0.447 |
| Q6ZUY8 | Lipase |  | 1 | 1 | 0.718 | 0.927 | 0.778 | 1.08 | 1.197 | 1.159 | 0.705 | 0.016 |
| Q6ZW64 | cDNA FLJ41552 fis, clone COLON2004478, highly similar to Protein Tro alpha1 H,myeloma |  | 175 | 2 | 1.126 | 0.911 | 0.998 | 0.757 | 0.554 | 0.836 | 1.413 | 0.052 |
| Q76LX8 | A disintegrin and metalloproteinase with thrombospondin motifs 13 | ADAMTS13 | 18 | 14 | 0.882 | 0.993 | 0.918 | 1.075 | 1.06 | 1.117 | 0.859 | 0.025 |
| Q7Z2U7 | Uncharacterized protein |  | 150 | 1 | 0.756 | 0.725 | 0.895 | 1.252 | 0.969 | 1.771 | 0.595 | 0.143 |
| Q7Z351 | Uncharacterized protein DKFZp686N02209 | DKFZp686N02209 | 512 | 1 | 0.522 | 1.181 | 0.46 | 0.726 | 0.939 | 0.763 | 0.891 | 0.744 |
| Q7Z3Y4 | Uncharacterized protein |  | 239 | 1 | 6.165 | 0.625 | 0.957 | 0.476 | 0.901 | 0.497 | 4.135 | 0.389 |
| Q7Z3Y6 | Rearranged VH4-34 V gene segment (Fragment) | VH4-34 | 18 | 0 | 0.831 | 1.042 | 0.866 | 0.838 | 0.809 | 0.932 | 1.062 | 0.526 |
| Q7Z7Q0 | APOB protein | APOB | 193 | 1 | 0.856 | 0.872 | 0.65 | 0.836 | 1.194 | 1.425 | 0.688 | 0.16 |
| Q86TT1 | Full-length cDNA clone CS0DD006YL02 of Neuroblastoma of Homo sapiens (human) |  | 1036 | 1 | 1.436 | 1.01 | 1.913 | 0.954 | 0.908 | 0.837 | 1.615 | 0.166 |
| Q86TV4 | Full-length cDNA clone CS0DI085YI08 of Placenta of Homo sapiens (human) (Fragment) |  | 6 | 4 | 1.011 | 1.054 | 1.686 | 0.448 | 0.419 | 0.445 | 2.858 | 0.065 |
| Q86U17 | Serpin A11 | SERPINA11 | 3 | 3 | 0.775 | 0.926 | 0.775 | 1.061 | 1.312 | 1.069 | 0.719 | 0.038 |
| Q86UD1 | Out at first protein homolog | OAF | 8 | 3 | 0.961 | 1.034 | 0.964 | 0.821 | 0.867 | 0.853 | 1.165 | 0.013 |
| Q86UX7 | Fermitin family homolog 3 | FERMT3 | 9 | 7 | 1.004 | 1.024 | 0.906 | 0.868 | 0.903 | 0.781 | 1.149 | 0.069 |
| Q86YW5 | Trem-like transcript 1 protein | TREML1 | 8 | 3 | 0.728 | 0.893 | 0.924 | 1.086 | 1.066 | 1.06 | 0.792 | 0.065 |
| Q86YZ3 | Hornerin | HRNR | 3 | 2 | 1.131 | 1.673 | 0.835 | 0.749 | 0.738 | 0.688 | 1.673 | 0.184 |
| Q8IUK5 | Plexin domain-containing protein 1 | PLXDC1 | 1 | 1 | 1.007 | 0.878 | 0.846 | 1.189 | 1.153 | 1.425 | 0.725 | 0.035 |
| Q8IUX7 | Adipocyte enhancer-binding protein 1 | AEBP1 | 1 | 1 | 1.002 | 1.078 | 0.881 | 1.037 | 0.939 | 0.971 | 1.005 | 0.949 |
| Q8IWM0 | Apolipoprotein J (Fragment) | CLU | 88 | 1 | 0.978 | 1.049 | 0.88 | 1.004 | 0.934 | 1.007 | 0.987 | 0.833 |
| Q8N1N4 | Keratin, type II cytoskeletal 78 | KRT78 | 17 | 9 | 0.82 | 0.978 | 1.044 | 1.012 | 0.989 | 1.185 | 0.892 | 0.275 |
| Q8N2S1 | Latent-transforming growth factor beta-binding protein 4 | LTBP4 | 1 | 1 | 0.938 | 0.934 | 0.907 | 1.002 | 1.099 | 1.049 | 0.882 | 0.036 |
| Q8N5F4 | IGL@ protein | IGL@ | 191 | 2 | 1.213 | 1.02 | 1.156 | 0.911 | 0.919 | 0.966 | 1.212 | 0.064 |
| Q8NBJ4 | Golgi membrane protein 1 | GOLM1 | 3 | 2 | 1.054 | 1.007 | 0.978 | 0.858 | 0.812 | 0.843 | 1.209 | 0.005 |
| Q8NBP7 | Proprotein convertase subtilisin/kexin type 9 | PCSK9 | 12 | 6 | 0.892 | 0.884 | 0.912 | 0.888 | 0.907 | 1.079 | 0.935 | 0.414 |
| Q8NCL6 | cDNA FLJ90170 fis, clone MAMMA1000370, highly similar to Ig alpha-1 chain C region |  | 177 | 1 | 1.12 | 1.283 | 1.577 | 1.103 | 0.926 | 0.958 | 1.332 | 0.118 |
| Q8NDZ4 | Deleted in autism protein 1 | C3orf58 | 1 | 1 | 0.941 | 1.12 | 1.197 | 0.898 | 0.938 | 0.971 | 1.161 | 0.179 |
| Q8NEJ1 | Uncharacterized protein |  | 162 | 2 | 0.794 | 1.009 | 0.831 | 0.956 | 0.922 | 0.97 | 0.925 | 0.396 |
| Q8NEZ3 | WD repeat-containing protein 19 | WDR19 | 16 | 1 | 0.818 | 0.782 | 0.785 | 0.989 | 0.77 | 1.234 | 0.797 | 0.269 |
| Q8NI99 | Angiopoietin-related protein 6 | ANGPTL6 | 10 | 6 | 1.067 | 0.981 | 1.051 | 0.99 | 0.887 | 0.957 | 1.093 | 0.096 |
| Q8TAQ9 | SUN domain-containing protein 3 | SUN3 | 5 | 2 | 0.913 | 1.005 | 0.791 | 1.259 | 1.085 | 1.075 | 0.793 | 0.051 |
| Q8TBD0 | Uncharacterized protein (Fragment) |  | 14 | 5 | 1.896 | 0.763 | 0.888 | 1.142 | 0.696 | 0.943 | 1.275 | 0.56 |
| Q8TCZ8 | Apolipoprotein E (Fragment) | APOE | 24 | 1 | 0.403 | 0.181 | 2.116 | 0.441 | 0.429 | 0.466 | 2.021 | 0.535 |
| Q8TER0 | Sushi, nidogen and EGF-like domain-containing protein 1 | SNED1 | 1 | 1 | 1.035 | 0.982 | 1.559 | 0.98 | 1.034 | 1.048 | 1.168 | 0.451 |
| Q8WTU2 | Scavenger receptor cysteine-rich domain-containing group B protein | SSC4D | 2 | 2 | 0.84 | 1.074 | 0.752 | 1.154 | 1.013 | 1.058 | 0.827 | 0.184 |
| Q8WVJ2 | NudC domain-containing protein 2 | NUDCD2 | 1 | 1 | 1.227 | 0.989 | 1.153 | 0.72 | 0.626 | 0.804 | 1.567 | 0.012 |
| Q8WW79 | L-selectin | SELL | 1 | 1 | 1.119 | 1.108 | 0.95 | 0.992 | 1.027 | 1.029 | 1.042 | 0.516 |
| Q8WWQ8 | Stabilin-2 | STAB2 | 9 | 7 | 1.066 | 0.981 | 1.026 | 1.075 | 0.973 | 1.054 | 0.991 | 0.82 |
| Q8WWZ8 | Oncoprotein-induced transcript 3 protein | OIT3 | 15 | 7 | 0.955 | 1.098 | 0.848 | 0.949 | 0.921 | 0.982 | 1.017 | 0.847 |
| Q8WXD2 | Secretogranin-3 | SCG3 | 2 | 1 | 1.087 | 0.953 | 0.941 | 1.119 | 1.114 | 1.155 | 0.88 | 0.092 |
| Q92496 | Complement factor H-related protein 4 | CFHR4 | 60 | 4 | 1.069 | 1.217 | 1.306 | 0.738 | 0.868 | 0.767 | 1.514 | 0.012 |
| Q92626 | Peroxidasin homolog | PXDN | 11 | 9 | 1.009 | 1.083 | 0.957 | 0.882 | 0.795 | 0.896 | 1.185 | 0.031 |
| Q92743 | Serine protease HTRA1 | HTRA1 | 1 | 1 | 0.953 | 1.029 | 1.11 | 0.911 | 0.901 | 0.796 | 1.185 | 0.053 |
| Q92896 | Golgi apparatus protein 1 | GLG1 | 3 | 3 | 0.941 | 0.86 | 0.855 | 1.108 | 1.029 | 1.977 | 0.645 | 0.25 |
| Q96IY4 | Carboxypeptidase B2 | CPB2 | 23 | 6 | 1.138 | 1.149 | 1.261 | 0.908 | 1.133 | 0.93 | 1.194 | 0.097 |
| Q96K68 | cDNA FLJ14473 fis, clone MAMMA1001080, highly similar to Homo sapiens SNC73 protein (SNC73) mRNA |  | 188 | 2 | 1.009 | 0.971 | 1.005 | 0.862 | 0.834 | 0.849 | 1.173 | 0.001 |
| Q96P63 | Serpin B12 | SERPINB12 | 3 | 2 | 0.855 | 0.764 | 1.662 | 1.086 | 0.951 | 1.192 | 1.017 | 0.956 |
| Q96PD5 | N-acetylmuramoyl-L-alanine amidase | PGLYRP2 | 29 | 9 | 0.802 | 1.101 | 0.897 | 1.107 | 1.149 | 1.042 | 0.849 | 0.193 |
| Q96QS0 | Putative matrix cell adhesion molecule-3 |  | 1 | 1 | 0.937 | 0.917 | 1.103 | 0.883 | 0.853 | 0.767 | 1.181 | 0.107 |
| Q96RZ2 | N-acetylglucosamine-1-phosphate transferase, gamma subunit, isoform CRA_b | RJD9 | 3 | 2 | 0.864 | 0.744 | 0.459 | 0.969 | 1 | 1.172 | 0.658 | 0.077 |
| Q99715 | Collagen alpha-1(XII) chain | COL12A1 | 4 | 4 | 0.837 | 0.855 | 1.358 | 1.067 | 1.147 | 1.145 | 0.908 | 0.609 |
| Q9BQB4 | Sclerostin | SOST | 1 | 1 | 1.503 | 0.9 | 1.468 | 1.044 | 0.999 | 0.955 | 1.291 | 0.275 |
| Q9BS26 | Endoplasmic reticulum resident protein 44 | ERP44 | 3 | 1 | 0.741 | 0.847 | 0.73 | 0.954 | 0.948 | 1.001 | 0.798 | 0.021 |
| Q9BTY2 | Plasma alpha-L-fucosidase | FUCA2 | 1 | 1 | 0.955 | 0.937 | 0.97 | 0.938 | 0.944 | 0.876 | 1.037 | 0.251 |
| Q9BUS0 | ZYX protein (Fragment) | ZYX | 1 | 1 | 0.956 | 1.14 | 1.242 | 0.834 | 0.857 | 0.813 | 1.334 | 0.076 |
| Q9BWP8 | Collectin-11 | COLEC11 | 6 | 4 | 0.921 | 0.944 | 0.898 | 1.045 | 0.959 | 0.944 | 0.937 | 0.179 |
| Q9BX93 | Group XIIB secretory phospholipase A2-like protein | PLA2G12B | 1 | 1 | 0.874 | 0.878 | 0.883 | 0.585 | 0.515 | 0.703 | 1.462 | 0.037 |
| Q9BXJ0 | Complement C1q tumor necrosis factor-related protein 5 | C1QTNF5 | 4 | 2 | 0.962 | 1.071 | 0.951 | 1.154 | 1.126 | 1.228 | 0.85 | 0.025 |
| Q9BY76 | Angiopoietin-related protein 4 | ANGPTL4 | 1 | 1 | 0.982 | 1.061 | 0.781 | 1.01 | 1.037 | 1.068 | 0.907 | 0.364 |
| Q9BYH1 | Seizure 6-like protein | SEZ6L | 1 | 1 | 1.02 | 0.883 | 0.805 | 1.145 | 1.108 | 1.274 | 0.768 | 0.03 |
| Q9GZP0 | Platelet-derived growth factor D | PDGFD | 3 | 2 | 0.878 | 1.065 | 0.97 | 0.946 | 0.901 | 0.943 | 1.044 | 0.532 |
| Q9H4B7 | Tubulin beta-1 chain | TUBB1 | 6 | 2 | 0.997 | 1.036 | 1.002 | 0.735 | 0.825 | 0.869 | 1.249 | 0.027 |
| Q9H6X2 | Anthrax toxin receptor 1 | ANTXR1 | 1 | 1 | 0.754 | 0.884 | 0.766 | 1.341 | 1.529 | 1.149 | 0.598 | 0.027 |
| Q9H8L6 | Multimerin-2 | MMRN2 | 3 | 2 | 0.888 | 0.915 | 1.032 | 1.164 | 0.996 | 0.95 | 0.911 | 0.316 |
| Q9HBI1 | Beta-parvin | PARVB | 7 | 5 | 0.875 | 0.948 | 0.934 | 0.904 | 0.828 | 0.837 | 1.073 | 0.129 |
| Q9HCC1 | Single chain Fv (Fragment) |  | 26 | 1 | 1.05 | 0.804 | 1.041 | 0.905 | 0.835 | 0.792 | 1.143 | 0.27 |
| Q9HCU0 | Endosialin | CD248 | 16 | 10 | 1.067 | 0.992 | 1.002 | 1.145 | 1.087 | 1.076 | 0.925 | 0.061 |
| Q9HD89 | Resistin | RETN | 4 | 3 | 1.321 | 1.181 | 1.432 | 1.082 | 0.881 | 1.153 | 1.263 | 0.068 |
| Q9HDC9 | Adipocyte plasma membrane-associated protein | APMAP | 8 | 6 | 0.871 | 1.027 | 1.605 | 0.656 | 0.691 | 0.608 | 1.792 | 0.146 |
| Q9NPP6 | Immunoglobulin heavy chain variant (Fragment) |  | 108 | 1 | 1.03 | 0.874 | 1.761 | 1.195 | 1.11 | 0.942 | 1.129 | 0.666 |
| Q9NQ79 | Cartilage acidic protein 1 | CRTAC1 | 6 | 4 | 1.067 | 1.498 | 1.024 | 0.993 | 1 | 0.948 | 1.22 | 0.289 |
| Q9NYU2 | UDP-glucose:glycoprotein glucosyltransferase 1 | UGGT1 | 1 | 1 | 0.916 | 0.943 | 1.12 | 0.706 | 0.863 | 0.706 | 1.31 | 0.049 |
| Q9NZP8 | Complement C1r subcomponent-like protein | C1RL | 13 | 3 | 0.925 | 1.043 | 0.993 | 1.045 | 1.018 | 1.113 | 0.932 | 0.184 |
| Q9NZZ3 | Charged multivesicular body protein 5 | CHMP5 | 1 | 1 | 1.213 | 1.03 | 1.057 | 0.945 | 0.868 | 0.976 | 1.183 | 0.077 |
| Q9UBS4 | DnaJ homolog subfamily B member 11 | DNAJB11 | 3 | 2 | 0.917 | 0.832 | 0.74 | 1.087 | 1.14 | 1.237 | 0.719 | 0.009 |
| Q9UBX1 | Cathepsin F | CTSF | 2 | 2 | 0.833 | 0.748 | 0.821 | 0.98 | 0.982 | 0.907 | 0.837 | 0.013 |
| Q9UGM5 | Fetuin-B | FETUB | 8 | 6 | 0.868 | 0.967 | 0.818 | 0.928 | 0.997 | 0.891 | 0.943 | 0.379 |
| Q9UHG3 | Prenylcysteine oxidase 1 | PCYOX1 | 15 | 6 | 0.895 | 0.941 | 0.832 | 1.199 | 1.17 | 1.045 | 0.781 | 0.016 |
| Q9UJU6 | Drebrin-like protein | DBNL | 1 | 1 | 0.979 | 0.953 | 1.158 | 1.11 | 0.824 | 1.332 | 0.946 | 0.74 |
| Q9UL72 | Myosin-reactive immunoglobulin heavy chain variable region (Fragment) |  | 45 | 1 | 2.665 | 0.959 | 0.981 | 0.895 | 0.852 | 0.711 | 1.874 | 0.332 |
| Q9UL82 | Myosin-reactive immunoglobulin light chain variable region (Fragment) |  | 9 | 2 | 1.133 | 0.865 | 0.801 | 0.643 | 0.653 | 0.797 | 1.337 | 0.133 |
| Q9UL83 | Myosin-reactive immunoglobulin light chain variable region (Fragment) |  | 8 | 1 | 1.022 | 0.832 | 1.167 | 0.905 | 0.857 | 0.941 | 1.117 | 0.39 |
| Q9UL84 | Myosin-reactive immunoglobulin heavy chain variable region (Fragment) |  | 32 | 2 | 1.171 | 0.954 | 1.263 | 1.116 | 0.943 | 0.947 | 1.127 | 0.315 |
| Q9UL86 | Myosin-reactive immunoglobulin kappa chain variable region (Fragment) |  | 10 | 2 | 1.031 | 0.825 | 1.002 | 0.944 | 0.87 | 0.878 | 1.062 | 0.489 |
| Q9UL88 | Myosin-reactive immunoglobulin heavy chain variable region (Fragment) |  | 30 | 3 | 0.797 | 1.02 | 0.931 | 0.875 | 0.84 | 0.84 | 1.076 | 0.426 |
| Q9UL89 | Myosin-reactive immunoglobulin heavy chain variable region (Fragment) |  | 4 | 2 | 1.125 | 0.857 | 1.596 | 0.426 | 0.418 | 0.357 | 2.98 | 0.066 |
| Q9UL92 | Myosin-reactive immunoglobulin heavy chain variable region (Fragment) |  | 6 | 1 | 1.126 | 0.763 | 2.003 | 0.558 | 0.762 | 0.607 | 2.02 | 0.214 |
| Q9UM07 | Protein-arginine deiminase type-4 | PADI4 | 1 | 1 | 0.783 | 0.726 | 0.555 | 1.382 | 1.247 | 1.372 | 0.516 | 0.003 |
| Q9UM47 | Neurogenic locus notch homolog protein 3 | NOTCH3 | 4 | 4 | 1.035 | 0.937 | 0.975 | 0.941 | 0.928 | 0.842 | 1.087 | 0.138 |
| Q9UNU2 | Complement protein C4B frameshift mutant (Fragment) | C4B | 142 | 2 | 0.898 | 0.773 | 0.908 | 1.109 | 1.273 | 1.071 | 0.747 | 0.022 |
| Q9Y279 | V-set and immunoglobulin domain-containing protein 4 | VSIG4 | 1 | 1 | 1.005 | 1.011 | 0.809 | 0.793 | 0.891 | 0.885 | 1.1 | 0.334 |
| Q9Y509 | VH3 protein (Fragment) | VH3 | 37 | 1 | 0.861 | 0.814 | 0.928 | 0.924 | 1.054 | 0.821 | 0.93 | 0.449 |
| Q9Y646 | Carboxypeptidase Q | CPQ | 1 | 1 | 0.804 | 0.777 | 0.756 | 0.954 | 0.99 | 1.053 | 0.78 | 0.007 |
| Q9Y6R7 | IgGFc-binding protein | FCGBP | 12 | 10 | 1.005 | 0.95 | 1.125 | 1.07 | 0.964 | 0.952 | 1.032 | 0.65 |
| Q9Y6Z7 | Collectin-10 | COLEC10 | 1 | 1 | 0.855 | 0.885 | 1.014 | 1.221 | 0.78 | 0.948 | 0.934 | 0.674 |
| S6AWD3 | IgG L chain |  | 194 | 1 | 1.054 | 1.008 | 1.19 | 0.898 | 0.731 | 1.236 | 1.135 | 0.486 |
| S6B294 | IgG L chain |  | 193 | 1 | 0.895 | 0.894 | 0.831 | 1.016 | 0.989 | 1.126 | 0.836 | 0.037 |
| S6B2A6 | IgG H chain |  | 122 | 1 | 0.975 | 1.111 | 1.062 | 0.902 | 0.898 | 0.819 | 1.202 | 0.027 |
| S6BAR0 | IgG L chain |  | 160 | 2 | 1.153 | 1.127 | 0.861 | 1.073 | 1.022 | 0.91 | 1.045 | 0.696 |
| S6BGD4 | IgG H chain |  | 79 | 1 | 0.969 | 1.067 | 0.993 | 0.815 | 1.143 | 0.9 | 1.06 | 0.629 |
| S6BGD6 | IgG L chain |  | 168 | 1 | 0.794 | 0.884 | 0.86 | 1.023 | 1.024 | 0.974 | 0.84 | 0.011 |
| S6C4S2 | IgG L chain |  | 169 | 1 | 1.142 | 0.827 | 0.734 | 0.757 | 1.233 | 0.801 | 0.969 | 0.89 |
| V9GYM3 | Apolipoprotein A-II | APOA2 | 152 | 6 | 0.907 | 0.983 | 0.853 | 1.143 | 1.044 | 1.224 | 0.804 | 0.03 |
| V9HVX6 | Epididymis luminal protein 9 | HEL-9 | 7 | 5 | 1.015 | 0.947 | 0.893 | 1.026 | 0.883 | 0.925 | 1.007 | 0.906 |
| V9HVX7 | Epididymis secretory protein Li 4 | HEL-S-4 | 2 | 1 | 0.81 | 0.846 | 0.842 | 0.861 | 0.952 | 0.73 | 0.983 | 0.843 |
| V9HVY3 | Protein disulfide-isomerase | HEL-S-269 | 3 | 3 | 0.908 | 1.004 | 0.942 | 0.919 | 1.18 | 0.928 | 0.943 | 0.58 |
| V9HW31 | ATP synthase subunit beta | HEL-S-271 | 3 | 3 | 0.678 | 0.813 | 0.626 | 1.153 | 1.361 | 1.026 | 0.598 | 0.022 |
| V9HW34 | Epididymis luminal protein 213 | HEL-213 | 241 | 1 | 0.948 | 0.954 | 1.045 | 1.034 | 1.065 | 0.976 | 0.958 | 0.358 |
| V9HW55 | Proteasome endopeptidase complex | HEL-S-275 | 2 | 2 | 0.857 | 0.899 | 0.771 | 1.283 | 1.184 | 1.361 | 0.66 | 0.003 |
| V9HW88 | Calreticulin, isoform CRA_b | HEL-S-99n | 16 | 8 | 0.856 | 0.935 | 0.853 | 0.882 | 0.901 | 0.773 | 1.034 | 0.583 |
| V9HW96 | Chaperonin containing TCP1, subunit 2 (Beta), isoform CRA_b | HEL-S-100n | 1 | 1 | 1.095 | 1.04 | 1.004 | 1.14 | 0.986 | 1.227 | 0.936 | 0.423 |
| V9HWB4 | Epididymis secretory sperm binding protein Li 89n | HEL-S-89n | 18 | 12 | 0.914 | 0.892 | 0.955 | 1.092 | 1.044 | 1.132 | 0.845 | 0.008 |
| V9HWE3 | Carbonic anhydrase I, isoform CRA_a | HEL-S-11 | 1 | 1 | 1.023 | 1.285 | 0.756 | 1.033 | 0.931 | 1.258 | 0.951 | 0.788 |
| V9HWF4 | Phosphoglycerate kinase | HEL-S-68p | 1 | 1 | 0.656 | 0.838 | 0.869 | 1.033 | 1.133 | 0.958 | 0.756 | 0.042 |
| V9HWF6 | Alpha-1-acid glycoprotein | HEL-S-153w | 8 | 2 | 1.155 | 0.856 | 0.729 | 0.803 | 0.666 | 0.634 | 1.303 | 0.229 |
| V9HWI6 | Epididymis secretory protein Li 51 | HEL-S-51 | 49 | 2 | 0.968 | 1.185 | 0.917 | 0.994 | 0.778 | 0.885 | 1.155 | 0.259 |
| V9HWP2 | Epididymis luminal protein 35 | HEL-S-125m | 41 | 17 | 0.875 | 0.749 | 0.784 | 1.078 | 1.034 | 1.003 | 0.773 | 0.01 |
| W0UV28 | Ribonuclease A A1 | RAA1 | 80 | 10 | 0.904 | 0.903 | 0.911 | 0.935 | 0.962 | 0.987 | 0.942 | 0.062 |
| W0UV93 | Ribonuclease A C1 | RAC1 | 5 | 1 | 1.108 | 0.955 | 0.963 | 0.946 | 0.96 | 1.04 | 1.028 | 0.67 |
| W8QEY1 | Lactoferrin |  | 153 | 2 | 0.962 | 1.096 | 1.062 | 1.242 | 1.125 | 1.22 | 0.87 | 0.046 |
| X6R8A1 | Carboxypeptidase | CTSA | 2 | 2 | 0.859 | 0.988 | 0.927 | 0.965 | 1.017 | 1.086 | 0.904 | 0.13 |

**Table S2** The significantly changed proteins from serum of moderate to high active rheumatoid arthritis (RA) patients *vs* healthy subjects by fold change > 1.2 and p value < 0.05 screening.

| Protein | Description | Gene | Unique_Peptides | Coverage [%] | PSMs | RA1 | RA2 | RA3 | Healthy subjects1 | Healthy subjects2 | Healthy subjects3 | FC (RA *vs* Healthy subjects) | *P* value (RA vs Healthy subjects) |
| --- | --- | --- | --- | --- | --- | --- | --- | --- | --- | --- | --- | --- | --- |
| P14555 | Phospholipase A2, membrane associated | PLA2G2A | 3 | 16 | 4 | 1.53 | 2.43 | 1.95 | 0.23 | 0.23 | 0.22 | 8.68 | 0.02 |
| P02741 | C-reactive protein | CRP | 7 | 25 | 80 | 1.19 | 1.58 | 1.39 | 0.42 | 0.22 | 0.3 | 4.41 | 0 |
| P0DJI9 | Serum amyloid A-2 protein | SAA2 | 2 | 43 | 7 | 1.98 | 1.37 | 1.39 | 0.49 | 0.42 | 0.5 | 3.34 | 0.03 |
| A2NYU7 | Heavy chain Fab (Fragment) | - | 1 | 7 | 2 | 1.07 | 0.94 | 1.23 | 0.37 | 0.37 | 0.4 | 2.85 | 0.01 |
| Q08830 | Fibrinogen-like protein 1 | FGL1 | 1 | 3 | 2 | 1.03 | 1.18 | 1.1 | 0.44 | 0.39 | 0.4 | 2.71 | 0 |
| F8W031 | Uncharacterized protein (Fragment) | - | 1 | 4 | 1 | 1.02 | 0.99 | 1 | 0.41 | 0.4 | 0.38 | 2.53 | 0 |
| P19652 | Alpha-1-acid glycoprotein 2 | ORM2 | 2 | 13 | 13 | 1.2 | 1 | 1.43 | 0.57 | 0.54 | 0.53 | 2.22 | 0.03 |
| A2N7P4 | Immunoglobulin mu-chain D-J4-region (Fragment) | IGHM | 1 | 22 | 6 | 0.97 | 1.08 | 0.88 | 0.44 | 0.46 | 0.45 | 2.18 | 0.01 |
| Q6PIQ7 | IGL@ protein | IGL@ | 1 | 40 | 184 | 1.12 | 1.14 | 1.47 | 0.57 | 0.54 | 0.64 | 2.14 | 0.02 |
| A0A0B4J1V6 | Immunoglobulin heavy variable 3-73 | IGHV3-73 | 1 | 34 | 15 | 0.89 | 1.19 | 1.03 | 0.5 | 0.53 | 0.47 | 2.06 | 0.02 |
| A0A024QZL1 | Proteoglycan 1, secretory granule, isoform CRA_a | PRG1 | 1 | 17 | 1 | 1.03 | 0.99 | 1.19 | 0.47 | 0.5 | 0.61 | 2.04 | 0 |
| D3DQX7 | Serum amyloid A protein | SAA1 | 2 | 48 | 15 | 1.21 | 1.13 | 1.1 | 0.61 | 0.53 | 0.55 | 2.03 | 0 |
| A0A0B4J1V2 | Immunoglobulin heavy variable 2-26 | IGHV2-26 | 2 | 12 | 2 | 1.52 | 1.16 | 1.22 | 0.6 | 0.74 | 0.67 | 1.95 | 0.02 |
| P55774 | C-C motif chemokine 18 | CCL18 | 2 | 35 | 5 | 1.59 | 1.38 | 1.23 | 0.68 | 0.73 | 0.91 | 1.81 | 0.01 |
| B4DT31 | cDNA FLJ53425, highly similar to Far upstream element-binding protein 1 | - | 1 | 2 | 1 | 1.07 | 1.04 | 1.05 | 0.66 | 0.55 | 0.54 | 1.8 | 0 |
| A2J1N5 | Rheumatoid factor RF-ET6 (Fragment) | - | 1 | 31 | 20 | 1.1 | 1 | 1.4 | 0.65 | 0.6 | 0.74 | 1.76 | 0.04 |
| Q6P089 | IGH@ protein | IGH@ | 1 | 28 | 102 | 1.31 | 1.32 | 1.46 | 0.83 | 0.72 | 0.78 | 1.75 | 0 |
| A0A024R0V4 | Vasodilator-stimulated phosphoprotein isoform 1 | VASP | 2 | 6 | 2 | 1.12 | 0.98 | 1.34 | 0.75 | 0.53 | 0.72 | 1.72 | 0.02 |
| Q06033 | Inter-alpha-trypsin inhibitor heavy chain H3 | ITIH3 | 17 | 22 | 109 | 0.99 | 0.98 | 1.25 | 0.64 | 0.62 | 0.62 | 1.7 | 0.04 |
| A0A096LPE2 | SAA2-SAA4 readthrough | SAA2-SAA4 | 5 | 26 | 36 | 0.93 | 1.12 | 1.22 | 0.67 | 0.61 | 0.66 | 1.68 | 0.03 |
| G1FM90 | Anti-Influenza A hemagglutinin heavy chain variable region (Fragment) | - | 2 | 22 | 9 | 1.52 | 1.09 | 1.38 | 0.85 | 0.7 | 0.91 | 1.62 | 0.04 |
| P03973 | Antileukoproteinase | SLPI | 11 | 48 | 29 | 1.1 | 1.06 | 1.09 | 0.66 | 0.69 | 0.7 | 1.58 | 0 |
| Q8WVJ2 | NudC domain-containing protein 2 | NUDCD2 | 1 | 7 | 1 | 1.23 | 0.99 | 1.15 | 0.72 | 0.63 | 0.8 | 1.57 | 0.01 |
| F5H423 | Uncharacterized protein | - | 2 | 9 | 3 | 1 | 0.93 | 1.24 | 0.67 | 0.64 | 0.72 | 1.56 | 0.05 |
| B7Z6Z4 | cDNA FLJ56329, highly similar to Myosin light polypeptide 6 | MYL6 | 2 | 12 | 2 | 1.22 | 1.24 | 1.18 | 0.9 | 0.75 | 0.75 | 1.52 | 0.01 |
| Q92496 | Complement factor H-related protein 4 | CFHR4 | 4 | 19 | 60 | 1.07 | 1.22 | 1.31 | 0.74 | 0.87 | 0.77 | 1.51 | 0.01 |
| P78386 | Keratin, type II cuticular Hb5 | KRT85 | 2 | 4 | 6 | 0.65 | 0.61 | 0.68 | 0.4 | 0.41 | 0.46 | 1.51 | 0 |
| P18428 | Lipopolysaccharide-binding protein | LBP | 10 | 15 | 117 | 1.01 | 1.13 | 1.23 | 0.79 | 0.71 | 0.74 | 1.5 | 0.02 |
| E7EMB3 | Calmodulin-2 | CALM2 | 5 | 20 | 20 | 1.28 | 1.12 | 1.12 | 0.88 | 0.76 | 0.74 | 1.48 | 0.01 |
| O15467 | C-C motif chemokine 16 | CCL16 | 2 | 23 | 2 | 1.04 | 0.99 | 0.8 | 0.6 | 0.57 | 0.77 | 1.46 | 0.04 |
| Q9BX93 | Group XIIB secretory phospholipase A2-like protein | PLA2G12B | 1 | 4 | 1 | 0.87 | 0.88 | 0.88 | 0.58 | 0.51 | 0.7 | 1.46 | 0.04 |
| P12838 | Neutrophil defensin 4 | DEFA4 | 1 | 11 | 1 | 1.07 | 1.17 | 0.96 | 0.71 | 0.67 | 0.81 | 1.46 | 0.02 |
| A0A0S2Z4F1 | EGF containing fibulin-like extracellular matrix protein 1 isoform 1 (Fragment) | EFEMP1 | 14 | 28 | 64 | 1.19 | 1.22 | 1.33 | 0.92 | 0.89 | 0.79 | 1.45 | 0 |
| H0YDW8 | Granulysin (Fragment) | GNLY | 1 | 4 | 1 | 1.13 | 1.32 | 1.16 | 0.86 | 0.79 | 0.9 | 1.42 | 0.01 |
| A2NW98 | Rheumatoid factor light chain variable region (Fragment) | - | 1 | 7 | 1 | 1.35 | 1.59 | 1.36 | 1.1 | 0.93 | 1.02 | 1.41 | 0.01 |
| A0A0C4DH36 | Immunoglobulin heavy variable 3-38 (non-functional) (Fragment) | IGHV3-38 | 2 | 19 | 2 | 1.14 | 1.27 | 1.12 | 0.82 | 0.82 | 0.86 | 1.41 | 0.01 |
| Q07507 | Dermatopontin | DPT | 3 | 10 | 6 | 1.31 | 1.3 | 1.32 | 1.01 | 0.88 | 0.92 | 1.4 | 0.01 |
| B2R5J8 | C-C motif chemokine | - | 2 | 21 | 11 | 1.21 | 1.17 | 1.09 | 0.85 | 0.86 | 0.8 | 1.38 | 0 |
| B4DPQ0 | cDNA FLJ54471, highly similar to Complement C1r subcomponent (EC 3.4.21.41) | C1R | 20 | 32 | 96 | 1.06 | 1.12 | 1.12 | 0.82 | 0.8 | 0.8 | 1.36 | 0 |
| A0A024RDE8 | PDZ and LIM domain 5, isoform CRA_c | PDLIM5 | 1 | 2 | 1 | 1.17 | 1.01 | 1.17 | 0.79 | 0.83 | 0.85 | 1.35 | 0.02 |
| P02649 | Apolipoprotein E | APOE | 29 | 70 | 296 | 0.99 | 1.11 | 1 | 0.81 | 0.78 | 0.77 | 1.31 | 0.02 |
| J3QRS3 | Myosin regulatory light chain 12A | MYL12A | 1 | 12 | 2 | 1.13 | 1.18 | 1.18 | 0.95 | 0.86 | 0.85 | 1.31 | 0 |
| Q59EN5 | Prosaposin variant (Fragment) | - | 7 | 13 | 10 | 1.08 | 0.94 | 1.03 | 0.81 | 0.78 | 0.74 | 1.31 | 0.02 |
| Q9NYU2 | UDP-glucose:glycoprotein glucosyltransferase 1 | UGGT1 | 1 | 1 | 1 | 0.92 | 0.94 | 1.12 | 0.71 | 0.86 | 0.71 | 1.31 | 0.05 |
| B2R950 | cDNA, FLJ94213, highly similar to Homo sapiens pregnancy-zone protein (PZP), mRNA | - | 18 | 19 | 94 | 1.15 | 1 | 1.04 | 0.8 | 0.87 | 0.76 | 1.31 | 0.01 |
| Q6IN99 | IGL@ protein | IGL@ | 1 | 40 | 156 | 1.14 | 1.07 | 0.97 | 0.79 | 0.9 | 0.75 | 1.3 | 0.02 |
| P51149 | Ras-related protein Rab-7a | RAB7A | 2 | 12 | 2 | 0.94 | 0.9 | 1.1 | 0.83 | 0.78 | 0.66 | 1.3 | 0.05 |
| B2RMS9 | Inter-alpha (Globulin) inhibitor H4 (Plasma Kallikrein-sensitive glycoprotein) | ITIH4 | 27 | 29 | 173 | 1.03 | 1.17 | 1.02 | 0.8 | 0.78 | 0.92 | 1.29 | 0.02 |
| Q5IWS5 | Intelectin 1 | ITLN1 | 8 | 31 | 28 | 1.13 | 1.24 | 1.14 | 0.95 | 0.93 | 0.85 | 1.29 | 0 |
| Q14314 | Fibroleukin | FGL2 | 1 | 2 | 2 | 1.27 | 1.23 | 1.14 | 0.99 | 0.89 | 0.98 | 1.27 | 0.01 |
| P61006 | Ras-related protein Rab-8A | RAB8A | 3 | 14 | 4 | 1.01 | 1.06 | 1.15 | 0.83 | 0.88 | 0.82 | 1.27 | 0.02 |
| P55056 | Apolipoprotein C-IV | APOC4 | 3 | 29 | 19 | 1.05 | 1.13 | 1.02 | 0.84 | 0.88 | 0.8 | 1.27 | 0.01 |
| O75558 | Syntaxin-11 | STX11 | 1 | 3 | 1 | 1.03 | 1.04 | 1.11 | 0.88 | 0.84 | 0.82 | 1.25 | 0 |
| B3KNX0 | cDNA FLJ30621 fis, clone CTONG2001681, highly similar to Complement C1s subcomponent (EC 3.4.21.42) | - | 19 | 34 | 83 | 1.03 | 1.05 | 1.12 | 0.9 | 0.82 | 0.85 | 1.25 | 0 |
| Q9H4B7 | Tubulin beta-1 chain | TUBB1 | 2 | 12 | 6 | 1 | 1.04 | 1 | 0.74 | 0.83 | 0.87 | 1.25 | 0.03 |
| A0A024R374 | Cathepsin B, isoform CRA_a | CTSB | 1 | 3 | 2 | 1.15 | 1.11 | 1.12 | 0.87 | 1 | 0.84 | 1.25 | 0.04 |
| P08246 | Neutrophil elastase | ELANE | 1 | 3 | 1 | 1.23 | 1.09 | 1.18 | 1.02 | 0.93 | 0.86 | 1.24 | 0.02 |
| P18065 | Insulin-like growth factor-binding protein 2 | IGFBP2 | 1 | 4 | 1 | 1 | 1 | 1.05 | 0.82 | 0.78 | 0.84 | 1.24 | 0 |
| P08493 | Matrix Gla protein | MGP | 1 | 11 | 3 | 1.09 | 1.14 | 1.07 | 0.78 | 0.93 | 0.95 | 1.24 | 0.04 |
| P61626 | Lysozyme C | LYZ | 8 | 49 | 51 | 1.09 | 0.95 | 1.05 | 0.8 | 0.87 | 0.85 | 1.23 | 0.03 |
| A0A2R8Y3M9 | Uncharacterized protein | - | 6 | 9 | 10 | 1.04 | 1.05 | 1.03 | 0.94 | 0.84 | 0.79 | 1.22 | 0.04 |
| A0A024RDE6 | Secreted phosphoprotein 1 (Osteopontin, bone sialoprotein I, early T-lymphocyte activation 1), isoform CRA_c | SPP1 | 3 | 12 | 5 | 1.01 | 0.97 | 0.9 | 0.83 | 0.72 | 0.81 | 1.22 | 0.02 |
| O75787 | Renin receptor | ATP6AP2 | 1 | 2 | 1 | 1.02 | 1.12 | 1.05 | 0.89 | 0.89 | 0.84 | 1.22 | 0.01 |
| Q53XB4 | Full-length cDNA clone CS0DF032YM23 of Fetal brain of Homo sapiens (human) | RAB1 | 6 | 41 | 31 | 1.17 | 1.03 | 1.16 | 0.92 | 0.93 | 0.93 | 1.21 | 0.05 |
| Q8NBJ4 | Golgi membrane protein 1 | GOLM1 | 2 | 7 | 3 | 1.05 | 1.01 | 0.98 | 0.86 | 0.81 | 0.84 | 1.21 | 0 |
| Q13201 | Multimerin-1 | MMRN1 | 21 | 19 | 39 | 1.03 | 1.15 | 1.12 | 0.91 | 0.89 | 0.93 | 1.21 | 0.03 |
| Q59H77 | T-complex protein 1 subunit gamma (Fragment) | - | 2 | 4 | 2 | 1.14 | 1.08 | 1.02 | 0.93 | 0.84 | 0.92 | 1.2 | 0.02 |
| S6B2A6 | IgG H chain | - | 1 | 27 | 122 | 0.98 | 1.11 | 1.06 | 0.9 | 0.9 | 0.82 | 1.2 | 0.03 |
| B3KUE5 | Phospholipid transfer protein, isoform CRA_c | PLTP | 3 | 6 | 9 | 0.88 | 0.92 | 0.92 | 1.12 | 1.09 | 1.07 | 0.83 | 0 |
| Q14515 | SPARC-like protein 1 | SPARCL1 | 10 | 21 | 25 | 0.96 | 0.88 | 0.82 | 1.11 | 1.08 | 1.02 | 0.83 | 0.03 |
| Q14112 | Nidogen-2 | NID2 | 9 | 8 | 17 | 0.97 | 0.91 | 0.87 | 1.09 | 1.07 | 1.18 | 0.83 | 0.01 |
| P02511 | Alpha-crystallin B chain | CRYAB | 1 | 6 | 4 | 0.84 | 0.83 | 0.79 | 1.01 | 0.95 | 1.04 | 0.82 | 0.01 |
| A5PL32 | APOL1 protein (Fragment) | APOL1 | 9 | 20 | 26 | 0.79 | 0.94 | 0.84 | 0.98 | 1.02 | 1.11 | 0.82 | 0.03 |
| A2N0T6 | VH6DJ protein (Fragment) | VH6DJ | 1 | 13 | 12 | 0.9 | 0.86 | 0.77 | 1.1 | 0.94 | 1.04 | 0.82 | 0.04 |
| M1LAK4 | Olfactomedin-like 3, isoform CRA_b | OLFML3 | 3 | 8 | 3 | 0.91 | 0.88 | 0.9 | 1.11 | 1.09 | 1.09 | 0.82 | 0 |
| P02545 | Prelamin-A/C | LMNA | 1 | 2 | 1 | 1.01 | 0.98 | 0.91 | 1.13 | 1.21 | 1.21 | 0.81 | 0.01 |
| B2R5M3 | cDNA, FLJ92530, highly similar to Homo sapiens chromogranin B (secretogranin 1) (CHGB), mRNA | - | 1 | 4 | 2 | 1.04 | 0.87 | 0.99 | 1.19 | 1.24 | 1.14 | 0.81 | 0.03 |
| P08567 | Pleckstrin | PLEK | 1 | 4 | 2 | 0.92 | 1 | 0.84 | 1.21 | 1.05 | 1.15 | 0.81 | 0.03 |
| A8K9M5 | cDNA FLJ77947, highly similar to Human complement protein C8 beta subunit mRNA | - | 11 | 17 | 28 | 0.88 | 0.99 | 0.9 | 1.07 | 1.18 | 1.2 | 0.8 | 0.01 |
| V9GYM3 | Apolipoprotein A-II | APOA2 | 6 | 17 | 152 | 0.91 | 0.98 | 0.85 | 1.14 | 1.04 | 1.22 | 0.8 | 0.03 |
| P05546 | Heparin cofactor 2 | SERPIND1 | 15 | 27 | 140 | 0.8 | 0.96 | 0.85 | 1 | 1.15 | 1.11 | 0.8 | 0.03 |
| B2R815 | cDNA, FLJ93695, highly similar to Homo sapiens serpin peptidase inhibitor, clade A (alpha-1 antiproteinase, antitrypsin), member 4 (SERPINA4), mRNA | - | 6 | 15 | 9 | 0.89 | 0.84 | 0.9 | 1.08 | 1.1 | 1.11 | 0.8 | 0 |
| Q9BS26 | Endoplasmic reticulum resident protein 44 | ERP44 | 1 | 3 | 3 | 0.74 | 0.85 | 0.73 | 0.95 | 0.95 | 1 | 0.8 | 0.02 |
| Q12841 | Follistatin-related protein 1 | FSTL1 | 2 | 5 | 3 | 0.92 | 0.99 | 0.78 | 1.09 | 1.18 | 1.11 | 0.8 | 0.05 |
| P58546 | Myotrophin | MTPN | 1 | 14 | 1 | 0.87 | 0.87 | 0.78 | 1.04 | 0.99 | 1.14 | 0.8 | 0.02 |
| A0A087WXB8 | ST3 beta-galactoside alpha-2,3-sialyltransferase 6, isoform CRA_b | ST3GAL6 | 2 | 4 | 2 | 0.8 | 0.91 | 0.95 | 1.04 | 1.17 | 1.13 | 0.8 | 0.02 |
| B3KQF5 | cDNA FLJ90381 fis, clone NT2RP2005035, highly similar to Calumenin | - | 1 | 14 | 18 | 0.9 | 0.93 | 0.77 | 1.11 | 1.07 | 1.09 | 0.8 | 0.04 |
| B2R7N9 | cDNA, FLJ93532, highly similar to Homo sapiens osteomodulin (OMD), mRNA | - | 2 | 4 | 7 | 1 | 0.98 | 0.83 | 1.29 | 1.16 | 1.09 | 0.79 | 0.04 |
| D3DNN4 | Carboxylic ester hydrolase | BCHE | 5 | 7 | 8 | 0.87 | 0.9 | 0.82 | 1.16 | 1.07 | 1.02 | 0.79 | 0.02 |
| Q59G70 | Mannosyl (Alpha-1,3-)-glycoprotein beta-1,2-N-acetylglucosaminyltransferase variant (Fragment) |  | 1 | 2 | 1 | 0.69 | 0.85 | 0.82 | 1 | 0.92 | 1.06 | 0.79 | 0.03 |
| B0AZL7 | cDNA, FLJ79457, highly similar to Insulin-like growth factor-binding proteincomplex acid labile chain | - | 11 | 18 | 16 | 0.82 | 0.84 | 0.87 | 1.06 | 1.05 | 1.1 | 0.78 | 0 |
| A0A0X9USK2 | MS-A6 heavy chain variable region (Fragment) | - | 1 | 10 | 1 | 1.05 | 0.88 | 0.91 | 1.12 | 1.32 | 1.18 | 0.78 | 0.03 |
| Q9UHG3 | Prenylcysteine oxidase 1 | PCYOX1 | 6 | 13 | 15 | 0.9 | 0.94 | 0.83 | 1.2 | 1.17 | 1.04 | 0.78 | 0.02 |
| Q9Y646 | Carboxypeptidase Q | CPQ | 1 | 2 | 1 | 0.8 | 0.78 | 0.76 | 0.95 | 0.99 | 1.05 | 0.78 | 0.01 |
| V9HWP2 | Epididymis luminal protein 35 | HEL-S-125m | 17 | 24 | 41 | 0.88 | 0.75 | 0.78 | 1.08 | 1.03 | 1 | 0.77 | 0.01 |
| Q9BYH1 | Seizure 6-like protein | SEZ6L | 1 | 1 | 1 | 1.02 | 0.88 | 0.8 | 1.14 | 1.11 | 1.27 | 0.77 | 0.03 |
| C9JD84 | Latent-transforming growth factor beta-binding protein 1 | LTBP1 | 2 | 21 | 97 | 0.87 | 0.9 | 0.92 | 1.22 | 1.11 | 1.18 | 0.77 | 0.01 |
| A0A024R5Z7 | Annexin | ANXA2 | 2 | 6 | 2 | 0.81 | 0.82 | 1 | 1.08 | 1.18 | 1.19 | 0.76 | 0.02 |
| B2R8I2 | cDNA, FLJ93914, highly similar to Homo sapiens histidine-rich glycoprotein (HRG), mRNA | - | 2 | 36 | 101 | 0.95 | 0.93 | 0.83 | 1.14 | 1.22 | 1.21 | 0.76 | 0 |
| P56202 | Cathepsin W | CTSW | 2 | 7 | 2 | 0.79 | 0.86 | 0.69 | 0.94 | 1.08 | 1.06 | 0.76 | 0.02 |
| V9HWF4 | Phosphoglycerate kinase | HEL-S-68p | 1 | 2 | 1 | 0.66 | 0.84 | 0.87 | 1.03 | 1.13 | 0.96 | 0.76 | 0.04 |
| A0A0C4DH25 | Immunoglobulin kappa variable 3D-20 | IGKV3D-20 | 1 | 28 | 10 | 0.81 | 0.85 | 0.84 | 1.18 | 1.14 | 0.98 | 0.76 | 0.04 |
| B7Z8Q2 | cDNA FLJ55606, highly similar to Alpha-2-HS-glycoprotein | - | 9 | 21 | 112 | 0.88 | 1 | 0.78 | 1.22 | 1.16 | 1.16 | 0.75 | 0.04 |
| B7Z6C2 | cDNA FLJ50663, highly similar to Phosphoglucomutase-1 (EC 5.4.2.2) | - | 1 | 1 | 1 | 0.76 | 0.82 | 0.87 | 1.13 | 0.99 | 1.14 | 0.75 | 0.01 |
| Q9UNU2 | Complement protein C4B frameshift mutant (Fragment) | C4B | 2 | 42 | 142 | 0.9 | 0.77 | 0.91 | 1.11 | 1.27 | 1.07 | 0.75 | 0.02 |
| A0A125U0U7 | MS-C1 heavy chain variable region (Fragment) | - | 1 | 25 | 8 | 0.75 | 0.75 | 0.83 | 0.96 | 1.09 | 1.09 | 0.74 | 0.01 |
| A0A090N8Y2 | Protein disulfide-isomerase A4 | ERP70 | 10 | 18 | 14 | 0.87 | 0.86 | 0.74 | 1.15 | 1.11 | 1.09 | 0.74 | 0.01 |
| A8K8U1 | cDNA FLJ77762, highly similar to Homo sapiens cullin-associated and neddylation-dissociated 1 (CAND1), mRNA | - | 1 | 1 | 1 | 0.91 | 0.85 | 0.79 | 1.23 | 1.07 | 1.16 | 0.74 | 0.01 |
| P23471 | Receptor-type tyrosine-protein phosphatase zeta | PTPRZ1 | 1 | 0 | 1 | 1.06 | 0.98 | 0.9 | 1.39 | 1.34 | 1.26 | 0.74 | 0 |
| A0A1L2BU38 | Anti-staphylococcal enterotoxin E heavy chain variable region (Fragment) | - | 1 | 7 | 2 | 0.8 | 1.03 | 0.84 | 1.1 | 1.2 | 1.37 | 0.73 | 0.03 |
| Q8IUK5 | Plexin domain-containing protein 1 | PLXDC1 | 1 | 2 | 1 | 1.01 | 0.88 | 0.85 | 1.19 | 1.15 | 1.42 | 0.72 | 0.04 |
| A0A140TA33 | Tenascin-X | TNXB | 13 | 7 | 14 | 0.89 | 0.8 | 0.78 | 1.16 | 1.16 | 1.1 | 0.72 | 0 |
| P05019 | Insulin-like growth factor I | IGF1 | 1 | 7 | 3 | 1.02 | 0.94 | 0.91 | 1.18 | 1.31 | 1.48 | 0.72 | 0.04 |
| Q15166 | Serum paraoxonase/lactonase 3 | PON3 | 8 | 32 | 60 | 0.89 | 0.87 | 0.75 | 1.04 | 1.28 | 1.15 | 0.72 | 0.02 |
| Q5HYE3 | Uncharacterized protein DKFZp686H1812 | DKFZp686H1812 | 1 | 2 | 1 | 0.87 | 0.96 | 0.76 | 1.2 | 1.16 | 1.23 | 0.72 | 0.02 |
| Q86U17 | Serpin A11 | SERPINA11 | 3 | 8 | 3 | 0.77 | 0.93 | 0.77 | 1.06 | 1.31 | 1.07 | 0.72 | 0.04 |
| Q9UBS4 | DnaJ homolog subfamily B member 11 | DNAJB11 | 2 | 5 | 3 | 0.92 | 0.83 | 0.74 | 1.09 | 1.14 | 1.24 | 0.72 | 0.01 |
| P01133 | Pro-epidermal growth factor | EGF | 1 | 1 | 1 | 0.81 | 0.83 | 0.87 | 1.11 | 1.15 | 1.25 | 0.72 | 0.01 |
| P36980 | Complement factor H-related protein 2 | CFHR2 | 5 | 32 | 227 | 0.86 | 0.89 | 0.81 | 1.22 | 1.3 | 1.09 | 0.71 | 0.02 |
| Q6ZUY8 | Lipase |  | 1 | 3 | 1 | 0.72 | 0.93 | 0.78 | 1.08 | 1.2 | 1.16 | 0.71 | 0.02 |
| B7Z1F8 | cDNA FLJ53025, highly similar to Complement C4-B | - | 2 | 62 | 538 | 0.77 | 0.9 | 0.68 | 1.09 | 1.16 | 1.09 | 0.7 | 0.02 |
| O94985 | Calsyntenin-1 | CLSTN1 | 3 | 4 | 3 | 0.86 | 0.83 | 0.8 | 1.12 | 1.19 | 1.24 | 0.7 | 0 |
| A0A1W6IYJ0 | N90-VRC38.03 heavy chain variable region (Fragment) | - | 1 | 15 | 1 | 0.75 | 0.81 | 0.92 | 1.31 | 1.18 | 1.08 | 0.69 | 0.01 |
| A2IPI6 | HRV Fab 027-VL (Fragment) | - | 1 | 20 | 6 | 0.77 | 1.01 | 0.71 | 1.14 | 1.11 | 1.35 | 0.69 | 0.04 |
| P49641 | Alpha-mannosidase 2x | MAN2A2 | 1 | 1 | 1 | 0.85 | 0.96 | 1.12 | 1.39 | 1.36 | 1.56 | 0.68 | 0.01 |
| P08833 | Insulin-like growth factor-binding protein 1 | IGFBP1 | 4 | 20 | 8 | 0.83 | 1.03 | 0.69 | 1.22 | 1.44 | 1.09 | 0.68 | 0.05 |
| A0A075B6R9 | Immunoglobulin kappa variable 2D-24 (non-functional) (Fragment) | IGKV2D-24 | 1 | 11 | 5 | 0.66 | 0.8 | 0.72 | 1.1 | 1.21 | 0.95 | 0.67 | 0.02 |
| Q04756 | Hepatocyte growth factor activator | HGFAC | 11 | 20 | 22 | 0.84 | 0.69 | 0.8 | 1.07 | 1.36 | 1.05 | 0.67 | 0.04 |
| V9HW55 | Proteasome endopeptidase complex | HEL-S-275 | 2 | 7 | 2 | 0.86 | 0.9 | 0.77 | 1.28 | 1.18 | 1.36 | 0.66 | 0 |
| A0A1L2BU40 | Anti-staphylococcal enterotoxin E variable region lambda chain (Fragment) | - | 1 | 36 | 18 | 0.6 | 0.78 | 0.59 | 0.91 | 1.07 | 1 | 0.66 | 0.01 |
| A0A024RC29 | Desmocollin 3, isoform CRA_b | DSC3 | 1 | 1 | 1 | 0.7 | 0.82 | 0.97 | 1.16 | 1.26 | 1.38 | 0.66 | 0.01 |
| A0N5G7 | Rheumatoid factor D5 heavy chain (Fragment) | VH3 | 1 | 9 | 2 | 0.9 | 0.76 | 0.91 | 1.37 | 1.29 | 1.3 | 0.65 | 0 |
| E2RVJ0 | Anion exchange protein | SLC4A1 | 1 | 1 | 1 | 0.69 | 0.73 | 0.66 | 1.15 | 1.04 | 1.09 | 0.63 | 0 |
| A0A024QZI2 | FCGRT | hCG_1998059 | 1 | 2 | 2 | 0.61 | 0.9 | 0.67 | 1.03 | 1.04 | 1.38 | 0.63 | 0.05 |
| A0A024R9Q1 | Thrombospondin 1, isoform CRA_a | THBS1 | 2 | 36 | 257 | 0.67 | 0.64 | 0.91 | 1.13 | 1.15 | 1.35 | 0.61 | 0.01 |
| A0A193CHR8 | 10E8 light chain variable region (Fragment) | - | 1 | 8 | 1 | 0.92 | 0.71 | 0.61 | 1.22 | 1.36 | 1.13 | 0.6 | 0.02 |
| Q9H6X2 | Anthrax toxin receptor 1 | ANTXR1 | 1 | 2 | 1 | 0.75 | 0.88 | 0.77 | 1.34 | 1.53 | 1.15 | 0.6 | 0.03 |
| V9HW31 | ATP synthase subunit beta | HEL-S-271 | 3 | 6 | 3 | 0.68 | 0.81 | 0.63 | 1.15 | 1.36 | 1.03 | 0.6 | 0.02 |
| A0A087WV75 | Neural cell adhesion molecule 1 | NCAM1 | 1 | 11 | 20 | 0.82 | 0.76 | 0.74 | 1.25 | 1.28 | 1.35 | 0.6 | 0 |
| B1N7B8 | Cryocrystalglobulin CC1 kappa light chain variable region (Fragment) | - | 1 | 35 | 14 | 0.61 | 0.74 | 0.57 | 1.16 | 0.95 | 1.13 | 0.59 | 0.01 |
| O75594 | Peptidoglycan recognition protein 1 | PGLYRP1 | 1 | 8 | 2 | 0.55 | 0.74 | 0.39 | 1.03 | 1.02 | 1.12 | 0.53 | 0.03 |
| Q9UM07 | Protein-arginine deiminase type-4 | PADI4 | 1 | 1 | 1 | 0.78 | 0.73 | 0.55 | 1.38 | 1.25 | 1.37 | 0.52 | 0 |

**Figures**


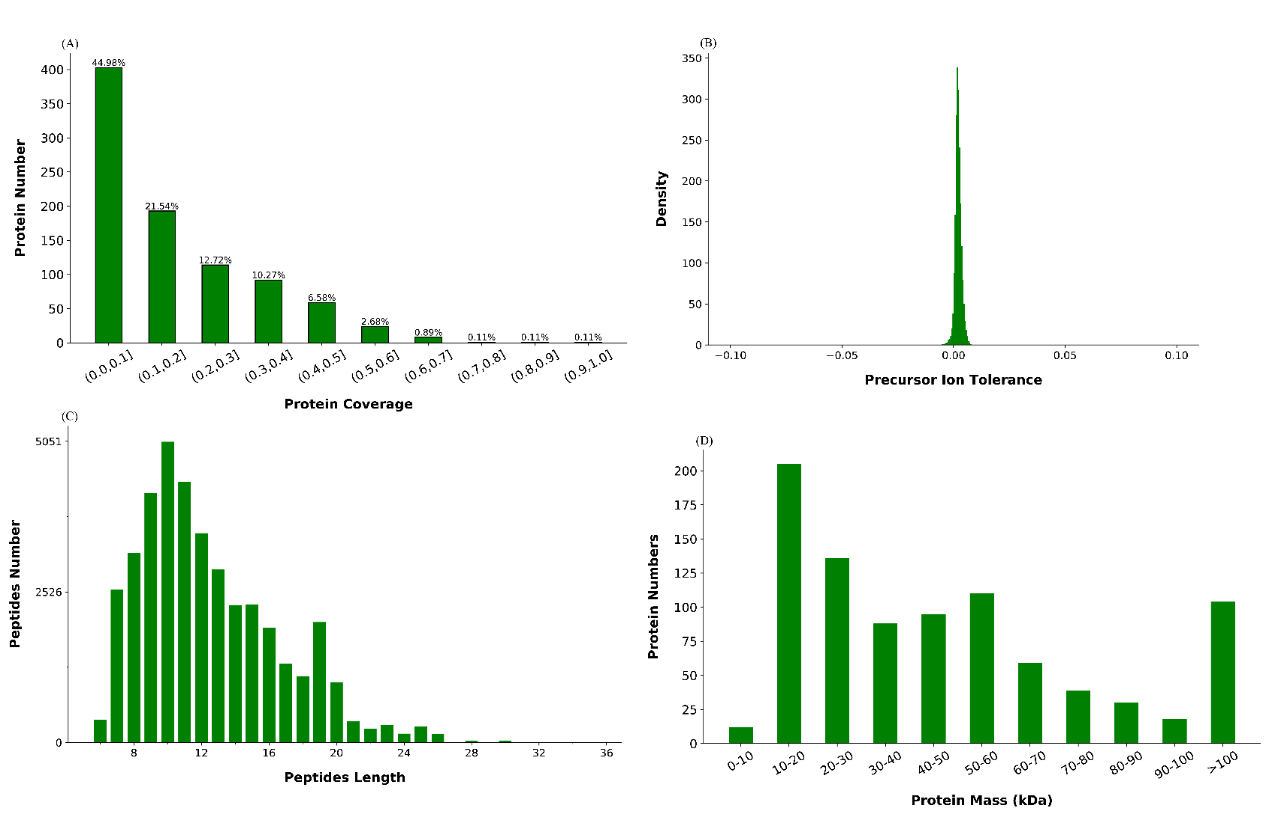


**Figure S1** Evaluation of the proteomic data from following parameters. A: protein coverage; B: precursor ion tolerance; C: peptides length; D: protein mass.


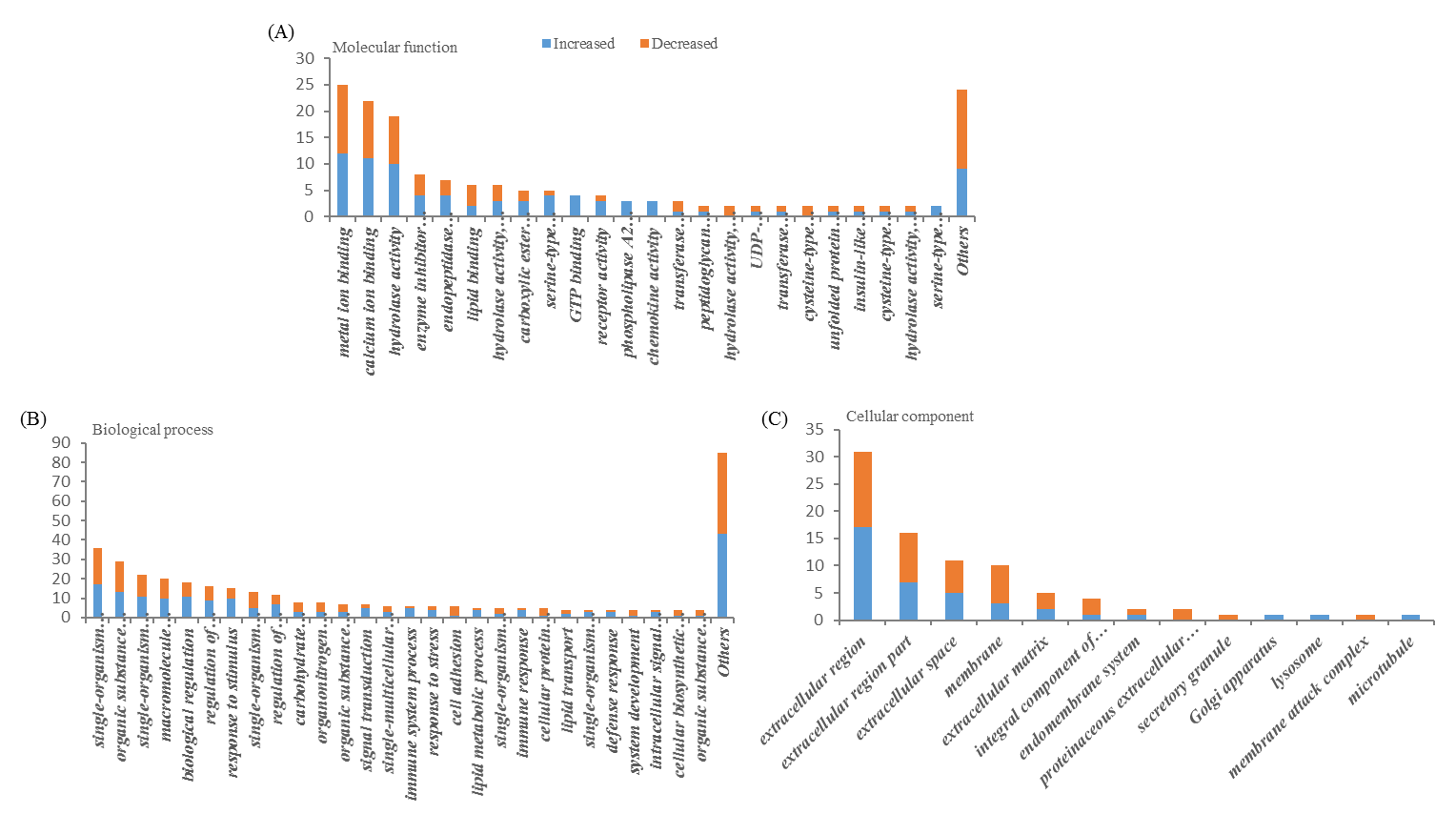


**Figure S2** GO functions for differential proteins (68 increased and 74 decreased proteins) from molecular function (A), biological process (B) and cellular component (C) angles. The differential proteins from serum of moderate to high active rheumatoid arthritis (RA) vs healthy subjects were analyzed based on fold change > 1.2 and *p* value < 0.05.


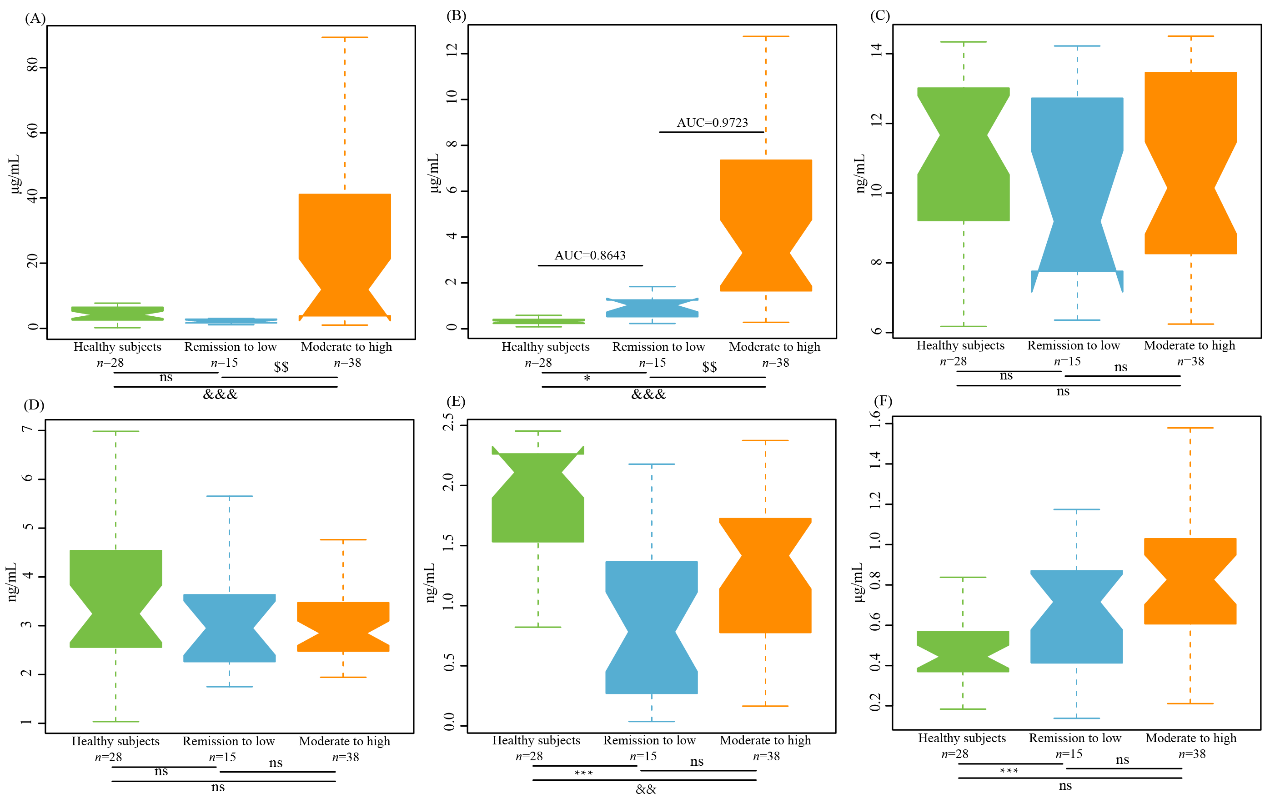


**Figure S3** The serum concentrations of C-reactive protein (CRP) (A), fibrinogen-like protein 1 (FGL1) (B), alpha-1-acid glycoprotein 2 (ORM2) (C), phospholipase A2 (PLA2) (D), serum amyloid A2 (SAA2) (E) and protein-arginine deiminase type-4 (PADI4) (F) in the cohort 2. AUC means area under receiver operating characteristic curve. (^*^) means significance with healthy subjects *vs* remission to low active rheumatoid arthritis (RA); (^&^) means significance with healthy subjects *vs* moderate to high active RA; (^$^) means significance with remission to low active RA *vs* moderate to high active RA; (^ns^) means non-significance. The significance of difference was analyzed by one-way ANOVA with Kruskal-Wallis nonparametric test.


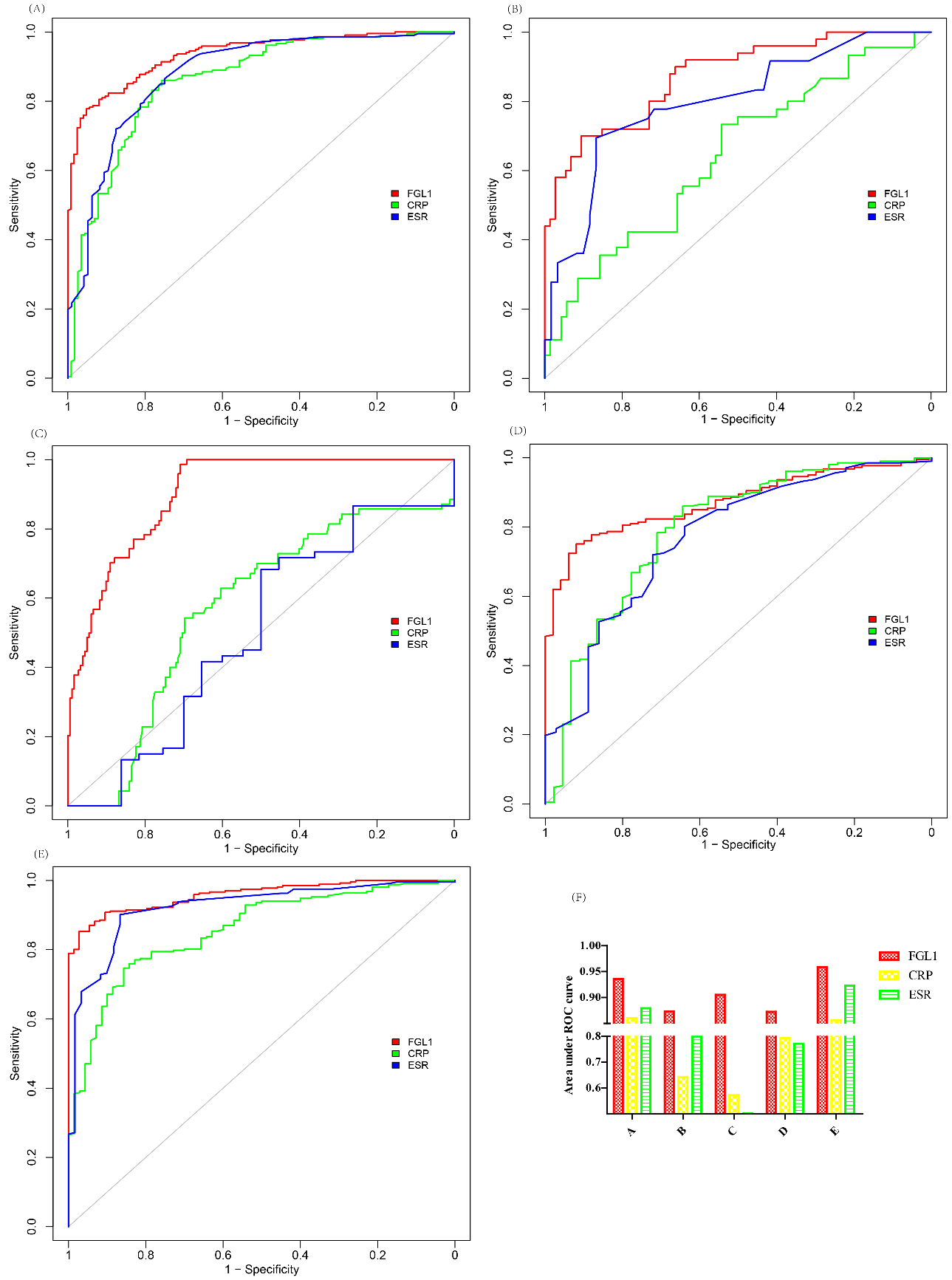


**Figure S4** Receiver operating characteristic (ROC) curve of fibrinogen-like protein 1 (FGL1), C-reactive protein (CRP) and erythrocyte sedimentation rate (ESR) in cross-comparison among moderate to high active RA, low active RA, RA in remission and healthy subjects. A: Moderate to high active RA *vs* remission to low active RA; B: Low active RA *vs* RA in remission; C: RA in remission *vs* healthy persons; D: Moderate to high active RA *vs* Low active RA; E: Low to high active RA *vs* RA in remission; F: The area under ROC curve (AUC) of FGL1, CRP and ESR in cross-comparison among moderate to high active RA, low active RA, RA in remission and healthy subjects.


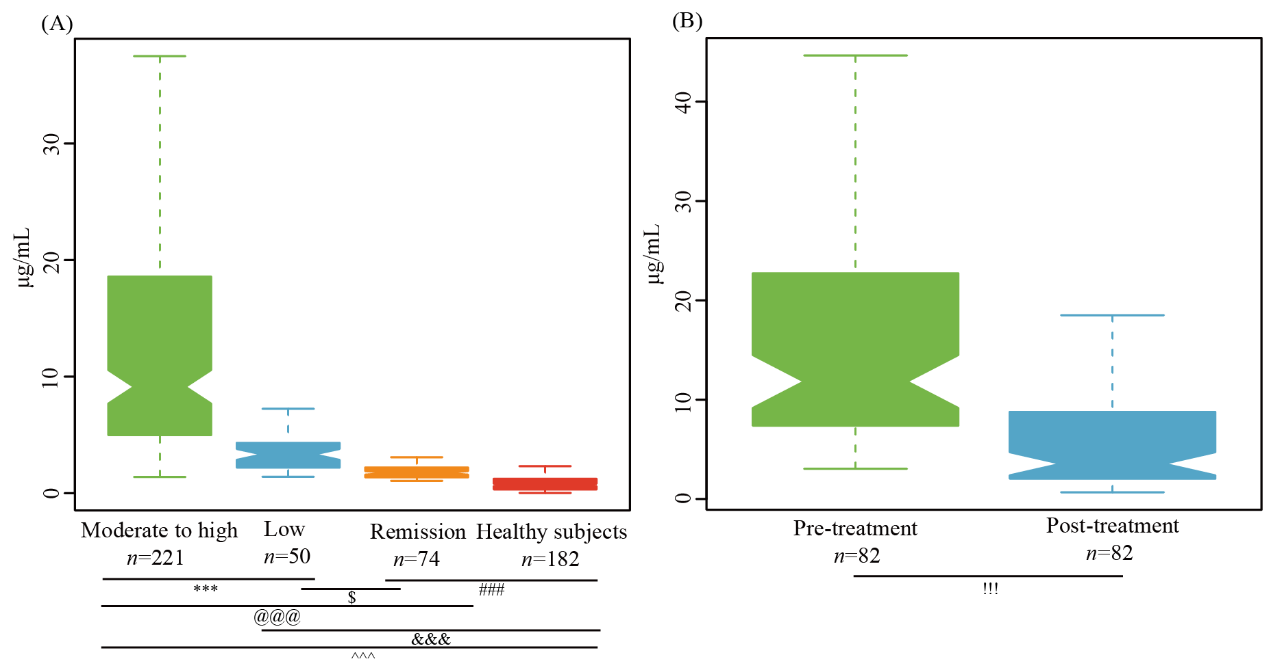


**Figure S5** The serum concentrations of fibrinogen-like protein 1 (FGL1) in the cohort 3 (discovery set) (A) and cohort 4 (B). (^*^) means significance with moderate to high active RA *vs* RA with low disease activity; (^$^) means significance with low active RA *vs* RA in remission; (^#^) means significance with RA in remission *vs* healthy subjects; (^@^) means significance with moderate to high active RA *vs* RA in remission; (^^^) means significance with moderate to high active RA *vs* healthy subjects; (^&^) means significance with low active RA *vs* healthy subjects. (^!^) means significance with pre-treated RA vs post-treated RA. The significant difference was analyzed by one-way ANOVA with Kruskal-Wallis nonparametric test (A) and Mann−Whitney U test (B).
